# A cross-sectional study of physical activity and chronic diseases among middle-aged and elderly in China

**DOI:** 10.1038/s41598-024-78360-z

**Published:** 2024-12-28

**Authors:** Yongyu Huang, Zuosheng Lu

**Affiliations:** School of Physical Education and Sport Science, South China Normal University, Guangzhou, China

**Keywords:** Public health, Physical activity, Chronic diseases, Middle-aged and elderly, Diseases, Risk factors

## Abstract

Regular physical activity is beneficial for both physical and mental health. However, studies on physical activity levels among middle-aged and elderly individuals in China are relatively scarce, and the association between different activity levels and certain chronic diseases remains unclear. Using cross-sectional data from the 2020 China Health and Retirement Longitudinal Study (CHARLS), this study describes the prevalence of chronic diseases and examines their associations with physical activity (PA). Participants self-reported the presence of fifteen chronic health conditions: hypertension, dyslipidemia, diabetes, cancer, chronic lung diseases, liver disease, heart attack, stroke, kidney disease, stomach diseases, emotional and mental issues, memory-related diseases, Parkinson’s disease, arthritis or rheumatism, and asthma. Total physical activity was assessed through self-reports using instruments similar to the International Physical Activity Questionnaire (IPAQ). Multivariate logistic regression was used to estimate the association between chronic diseases and PA. In China, more than one-sixth of middle-aged and elderly individuals do not meet physical activity guidelines. Compared to those with low PA, individuals with moderate PA were associated with a lower prevalence of four chronic diseases (heart disease, stroke, emotional and mental issues, asthma), while those with high PA were associated with a lower prevalence of nine chronic diseases (hypertension, dyslipidemia, diabetes, cancer, heart attack, stroke, emotional and mental issues, memory-related diseases, asthma). Higher physical activity levels are associated with lower odds of chronic diseases. These associations between physical activity and various chronic diseases are inconsistent. Future research should employ cohort or experimental studies to determine the causal relationships between physical activity and the prevalence of different chronic diseases.

**Supplementary Information:**

The online version contains supplementary material available at 10.1038/s41598-024-78360-z.

## Introduction

As the global aging trend accelerates, the health of the elderly has become a focal point of global attention^1,2^. This is particularly evident in China^3^, where the rapid growth of the elderly population poses significant public health challenges^4^. Multiple studies have shown that older adults are more likely to suffer from chronic diseases, which severely impact their quality of life^5^.

Chronic diseases, including cardiovascular diseases, diabetes, and cancer, represent major public health challenges worldwide. According to reports by the World Health Organization (WHO), chronic diseases account for 68% of global deaths^6^. Furthermore, 7.2% of all-cause deaths and 7.6% of cardiovascular deaths are attributable to physical inactivity^7^. While chronic diseases can be caused by various factors such as environmental and genetic factors, physical inactivity is recognized as one of the primary preventable factors^8–10^. Numerous studies have demonstrated that higher physical activity can reduce the prevalence of many chronic diseases^10,11^.

In 2020, the WHO developed and released international guidelines for physical activity. These guidelines recommend that adults engage in at least 150 min of moderate-intensity aerobic physical activity, or at least 75 min of vigorous-intensity aerobic physical activity per week, or an equivalent combination of both^9^. A representative cross-sectional survey indicated that 36.2% of individuals across the European Union are insufficiently active, with Portugal having the highest proportion at 63.7% and Sweden the lowest at 19.2%^12^. According to the Behavioral Risk Factor Surveillance System, the proportion of insufficiently active Americans decreased from 24.5% in 2018 to 23.8% in 2020^13^. In China, this proportion increased from 17.9% in 2010 to 22.3% in 2018^14^.

Research has shown that physical activity helps reduce systemic inflammation levels, improve metabolic health (for example, by increasing insulin sensitivity and improving lipid profiles), and enhance cardiovascular system function^15–17^. These changes collectively reduce the risk of chronic diseases. Additionally, regular physical activity has been proven to significantly reduce symptoms of depression and anxiety, improve individuals’ quality of life and sense of self-efficacy, and enhance mental health by strengthening social connections and reducing feelings of loneliness^15^. Therefore, reducing the proportion of the population that is insufficiently active is crucial in the field of public health in the twenty-first century^7,9^.

Despite the widely recognized benefits of physical activity in preventing chronic diseases, existing research often focuses on specific diseases, lacks comprehensive studies on middle-aged and elderly populations, and is limited in the context of Chinese individuals^18–20^. This study addresses these gaps by examining the association between varying physical activity levels and a broad range of chronic conditions among middle-aged and elderly individuals in China.

## Methods

### Study sample

The participants of this study were derived from the China Health and Retirement Longitudinal Study (CHARLS), which aims to collect high-quality microdata on households and individuals aged 45 and above in China. This data are used to analyze the aging population and promote interdisciplinary research on aging. The national baseline survey was conducted in 2011 using the multi-stage probability proportional to size (PPS) sampling method, ensuring comprehensive representativeness of the research data. Since 2011, the CHARLS survey has been conducted nationwide, covering 28 provinces, 150 county-level units, and 450 village-level units, initially encompassing approximately 10,000 households and 17,708 middle-aged and elderly individuals^21^. Utilizing systematic sampling methods, the fifth round (2020) of the survey included 19,395 participants. This study included 18,503 samples aged 45 to 91 years. The exclusion criteria were as follows:

1. 237 individuals under the age of 45.
2. 488 individuals with missing variables (including 28 missing health status and function information, 400 missing sleep information, 34 missing per capita household consumption expenditure (PCE) information, 4 missing disease information, 12 missing physical activity information, and 10 missing residential area information).
3. 167 outliers in age and sleep data, defined as values beyond the mean ± 3 standard deviations.

### Measures

#### Chronic diseases

The collection of chronic disease conditions included the prevalence of 15 common chronic diseases: hypertension, dyslipidemia, diabetes, cancer, chronic lung diseases, liver disease, heart attack, stroke, kidney disease, stomach diseases, emotional and mental issues, memory-related diseases, Parkinson’s disease, arthritis or rheumatism, and asthma. To gather this information, every participant was asked the following question: "Has a doctor ever told [XRName] that they have [XChroDisType[i]]?" Additionally, since CHARLS is a national longitudinal survey, a portion of the 18,503 participants had already been diagnosed with diseases in the 2018 survey; hence, these specific questions were not revisited in the 2020 survey. The final sample of diagnosed individuals consisted of those confirmed to have diseases in 2020 combined with those diagnosed in 2018.

#### Physical activity

The physical activity questionnaire is part of the CHARLS survey, specifically found on pages 70–71 of the CHARLS questionnaire. Participants were asked to report the number of days they engaged in light, moderate, or vigorous physical activity in a usual week, as well as the specific time spent on each type of physical activity per day (only activities lasting more than 10 min at a time were included). This section is similar to the short version of the International Physical Activity Questionnaire (IPAQ), but there are three key differences between CHARLS and IPAQ:

1. CHARLS collects physical activity information for “a usual week” instead of “the last 7 days.” Previous studies have shown similar performance between these two reference periods.
2. CHARLS does not collect information on sedentary behavior.
3. CHARLS uses four discrete time durations instead of continuous values.

Based on the methods used in previous studies^22,23^, the median value of each time duration category was used (the “more than 4 h” category was assigned a value of 4 h). Following established guidelines for IPAQ data processing and analysis, total activity time (including work, leisure, and exercise) was calculated by multiplying the reported duration (in minutes) of different types of physical activity by the number of days that activity was performed. The metabolic equivalents (METs) of each activity (light = 3.3, moderate = 4.0, and vigorous = 8.0) were multiplied by the number of minutes per week to provide an estimate of total MET-minutes per week. Total physical activity was then categorized as low (< 600 MET × minutes per week), moderate (600–3000 MET × minutes per week), and high (> 3000 MET × minutes per week).

### Covariates

The sociodemographic characteristics of participants (gender, age, residential area, education level, sleep, drinking status, marital status, smoking status, and per capita household consumption expenditure (PCE)) were collected using standardized questionnaires as part of the CHARLS survey. Gender, residential area, education level, drinking status, marital status, and smoking status as categorical variables, while age, sleep, and PCE were treated as continuous variables. In this study, we adjusted for potential confounding factors in the multivariate logistic regression analysis to assess the associations between physical activity levels and the prevalence of chronic diseases. Model 1 was the unadjusted multivariate logistic regression model. Model 2 was adjusted for age and gender. Model 3 was additionally adjusted for marital status, education level, residential area, and PCE. Model 4 was further adjusted for sleep, drinking status, and smoking status.

### Statistical analysis

Sample characteristics were presented as means ± standard deviations for continuous variables and as frequencies and percentages for categorical variables, stratified by low, moderate, and high overall physical activity levels. To examine the associations between physical activity levels and demographic variables, lifestyle factors, and chronic diseases, Pearson chi-square tests and ANOVA were employed, depending on the type of variables. A p-value of less than 0.05 was considered statistically significant. Multiple logistic regression was used to explore the associations between different physical activity levels and the prevalence of chronic diseases. First, the low physical activity population was used as a reference, and after adjusting for all covariates, the odds ratios (OR) and 95% confidence intervals (CI) for moderate and high physical activity levels were calculated. Secondly, using moderate physical activity as a reference, after adjusting for all covariates, the OR and 95% CI for high physical activity were plotted using a forest plot. All data analyses were performed using R software version 4.3.3.

## Results

This study analyzed data from 18,503 participants aged 45 to 91 to explore the association between physical activity levels and the prevalence of chronic diseases. Detailed demographic characteristics and lifestyle features are summarized in Table 1. In the overall sample, the average age was 61 ± 9.6 years. Females (52.3%) slightly outnumbered males (47.7%), and rural residents (63.2%) were more prevalent than urban–rural (12.3%), urban (24.3%), and special regions (1.2%) residents. Among the participants, 3,071 (16.6%) reported low physical activity, 4,947 (26.7%) reported moderate physical activity, and 10,485 (56.7%) reported high physical activity, with 15,432 (83.4%) meeting the guidelines for physical activity (≥ 600 MET × min per week). Among the 15 chronic diseases, the highest prevalence was found in Hypertension (41.4%) and arthritis or rheumatism (40.9%), while the lowest was found in Parkinson’s disease (2.6%) and cancer (2.8%). Younger individuals, those with higher education levels, and urban populations were more likely to meet the physical activity guidelines. However, people from rural areas and those with lower education levels are more likely to achieve higher levels of physical activity. Using chi-square tests and ANOVA, we found significant associations between all sociodemographic and lifestyle variables and physical activity levels (*P* < 0.05).

**Table 1:** Participant characteristics stratified by total physical activity.

	Overall	LPA^d^ (0–600 MET × min per week)	MPA^d^ (600–3000 MET × min per week)	HPA^d^ (> 3000 MET × min per week)	P-value
	18,503	3071 (16.6)	4947 (26.7)	10,485 (56.7)	
Age (years)X ± SD	61.6 (9.6)	65.4 (10.8)	62.4 (9.7)	60.1 (8.8)	0.001
Sleep (hours)X ± SD	6.1 (1.8)	6.0 (2.1)	6.1 (1.7)	6.1 (1.8)	0.045
PCE (yuan)X ± SD	2378.2 (3084.5)	2064.3 (2451.5)	2670.6 (3408.2)	2332.1 (3078.9)	0.001
Sex					0.029
Male	8822 (47.7)	1397 (15.8)	2382 (27.0)	5043 (57.2)	0.001
Female	9681 (52.3)	1674 (17.3)	2565 (26.5)	5442 (56.2)	
Education					0.001
None	3963 (21.4)	913 (23.0)	815 (20.6)	2235 (56.4)	
Primary	7963 (43.0)	1355 (17.0)	1976 (24.8)	4632 (58.2)	
Secondary	6158 (33.3)	766 (12.4)	1970 (32.0)	3422 (55.6)	
College or university	419 (2.3)	37 (8.8)	186 (44.4)	196 (46.8)	
Residential area					0.001
Urban	4499 (24.3)	636 (14.1)	1657 (36.8)	2206 (49.0)	
Urban and rural	2280 (12.3)	372 (16.3)	706 (31.0)	1202 (52.7)	
Rural	11,711 (63.2)	2060 (17.6)	2579 (22.0)	7062 (60.4)	
Special regions	23 (1.2)	3 (13.0)	5 (21.7)	15 (65.2)	
Smoke status^b^					0.001
Current smoker	4777 (25.8)	761 (15.9)	1198 (25.1)	2818 (59.0)	
Quit smoking	2483 (13.4)	471 (19.0)	752 (30.3)	1260 (50.7)	
Never smoker	11,243 (60.8)	1839 (16.4)	2997 (26.7)	6407 (57.0)	
Drinking status^c^					0.001
> Once/per week	4969 (26.9)	663 (13.3)	1272 (25.6)	3034 (61.1)	
< Once/per week	1752 (9.5)	214 (12.2)	475 (27.1)	1063 (60.7)	
Never drink	11,782 (63.7)	2194 (18.6)	3200 (27.2)	6388 (54.2)	
Marital status					0.001
Married	15,608 (84.4)	2333 (14.9)	4096 (26.2)	9179 (58.8)	
Separated	88 (0.5)	16 (18.2)	20 (22.7)	52 (59.1)	
Divorced	252 (1.4)	33 (13.1)	97 (38.5)	122 (48.4)	
Widowed	2448 (13.2)	654 (26.7)	710 (29.0)	1084 (44.3)	
Never married	107 (0.6)	35 (32.7)	24 (22.4)	48 (44.9)	
Chronic					
Hypertension	7670 (41.4)	1509 (49.1)	2214 (44.8)	3947 (37.6)	0.001
Dyslipidemia	5189 (28.0)	944 (30.7)	1588 (32.1)	2657 (25.3)	0.001
Diabetes	2937 (15.9)	538 (17.5)	919 (18.6)	1480 (14.1)	0.001
Cancer	524 (2.8)	107 (3.5)	177 (3.6)	240 (2.3)	0.001
Chronic lung disease	2911 (15.7)	572 (18.6)	781 (15.8)	1558 (14.9)	0.001
Liver disease	1518 (8.2)	251 (8.2)	432 (8.7)	835 (8.0)	0.267
Heart disease	4103 (22.2)	885 (28.8)	1203 (24.3)	2015 (19.2)	0.001
Stroke	1525 (8.2)	406 (13.2)	469 (9.5)	650 (6.2)	0.001
Kidney disease	2144 (11.6)	361 (11.8)	613 (12.4)	1170 (11.2)	0.079
Stomach disease	6145 (33.2)	998 (32.5)	1658 (33.5)	3489 (33.3)	0.628
Emotional and mental issues	573 (3.1)	165 (5.4)	162 (3.3)	246 (2.3)	0.001
Alzheimer’s disease	757 (4.1)	201 (6.5)	225 (4.5)	331 (3.2)	0.001
Parkinson’s disease	485 (2.6)	111 (3.6)	137 (2.8)	237 (2.3)	
Arthritis or rheumatism	7571 (40.9)	1310 (42.7)	1908 (38.6)	4353 (41.5)	0.001
Asthma	1196 (6.5)	323 (10.5)	312 (6.3)	561 (5.4)	

^a^Data are mean (SD) or n (%). MET = metabolic equivalents.

^b^In the context of smoking status, current smokers are individuals who smoke and continue to do so. Quit smoking are those who used to smoke but are currently in the process of quitting. Non-smokers are individuals who have never smoked.

^c^The terms > Once/per week and < Once/per week describe the frequency of alcohol consumption among drinkers, while Never drink refers to individuals who never consume alcohol.

^d^LPA = low physical activity, MPA = moderate physical activity, HPA = high physical activity.

**Fig. 1:**
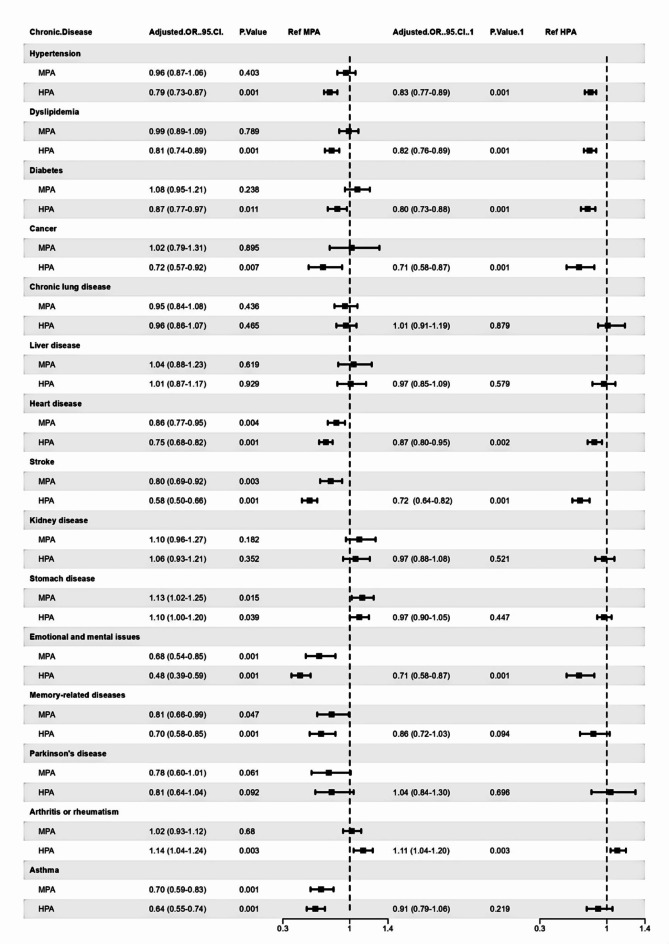
Forest Plot of Odds ratios and 95% CIs for 15 chronic diseases. ^a^Data adjusted for age, sex, education, sleep, residential area, smoking status, drinking status, marital status, and PCE. ^b^Low physical activity =  < 600 MET × min per week. Moderate physical activity = 600–3000 MET × min per week. High physical activity =  > 3000 MET × min per week.

In this study, we adjusted for all covariates in the multivariate logistic regression analysis to assess the associations between physical activity levels and the prevalence of chronic diseases. Compared with low physical activity, moderate physical activity showed significantly lower disease prevalence in four out of the 15 chronic diseases (heart disease, stroke, emotional and mental issues, asthma), but an increased prevalence of stomach disease (OR = 1.13, 95% CI:1.02–1.25). For the moderate physical activity group, the largest reduction in disease prevalence was observed for emotional and mental issues (OR = 0.68, 95% CI: 0.54–0.86). Those with high physical activity had a lower prevalence for nine diseases (hypertension, dyslipidemia, diabetes, cancer, heart attack, stroke, emotional, memory-related diseases, asthma). However, the prevalence of stomach disease and arthritis or rheumatism increased (OR = 1.10, 95% CI: 1.00–1.20), (OR = 1.13, 95% CI: 1.04–1.23). For the high physical activity group, the greatest risk prevalence reductions were observed for emotional and mental issues (OR = 0.48, 95% CI: 0.36–0.59) and stroke (OR = 0.58, 95% CI: 0.50–0.66), as shown in Table 2 and Fig. 1.

**Table 2:** Odds ratios for 15 chronic diseases by physical activity level.

	N	Model 1	Model 2	Model 3	Model 4
Hypertension					
Low physical activity	1509	1.00 (Ref)	1.00 (Ref)	1.00 (Ref)	1.00 (Ref)
Moderate physical activity	2214	0.84 (0.77–0.92)	0.95 (0.87–1.05)	0.96 (0.87–1.05)	0.96 (0.87–1.06)
High physical activity	3947	0.62 (0.58–0.68)	0.78 (0.72–0.85)	0.79 (0.72–0.86)	0.79 (0.73–0.87)
Dyslipidemia					
Low physical activity	944	1.00 (Ref)	1.00 (Ref)	1.00 (Ref)	1.00 (Ref)
Moderate physical activity	1588	1.07 (0.97–1.17)	1.11 (1.01–1.22)	0.98 (0.89–1.09)	0.99 (0.89–1.09)
High physical activity	2657	0.76 (0.70–0.84)	0.82 (0.75–0.89)	0.80 (0.73–0.88)	0.81 (0.74–0.89)
Diabetes					
Low physical activity	538	1.00 (Ref)	1.00 (Ref)	1.00 (Ref)	1.00 (Ref)
Moderate physical activity	919	1.07 (0.96–1.21)	1.14 (1.01–1.29)	1.07 (0.95–1.21)	1.08 (0.95–1.21)
High physical activity	1480	0.77 (0.69–0.86)	0.86 (0.77–0.96)	0.85 (0.76–0.95)	0.87 (0.77–0.97)
Cancer					
Low physical activity	107	1.00 (Ref)	1.00 (Ref)	1.00 (Ref)	1.00 (Ref)
Moderate physical activity	177	1.03 (0.81–1.32)	1.07 (0.84–1.37)	0.99 (0.78–1.28)	1.02 (0.79–1.31)
High physical activity	240	0.65 (0.52–0.82)	0.69 (0.54–0.88)	0.68 (0.54–0.87)	0.72 (0.57–0.92)
Chronic lung disease					
Low physical activity	572	1.00 (Ref)	1.00 (Ref)	1.00 (Ref)	1.00 (Ref)
Moderate physical activity	781	0.82 (0.73–0.92)	0.91 (0.80–1.02)	0.94 (0.83–1.06)	0.95 (0.84–1.08)
High physical activity	1558	0.76 (0.69–0.85)	0.92 (0.83–1.03)	0.93 (0.83–1.03)	0.96 (0.86–1.07)
Liver disease					
Low physical activity	251	1.00 (Ref)	1.00 (Ref)	1.00 (Ref)	1.00 (Ref)
Moderate physical activity	432	1.07 (0.91–1.27)	1.09 (0.93–1.28)	1.03 (0.88–1.22)	1.04 (0.88–1.23)
High physical activity	835	0.97 (0.84–1.13)	1.00 (0.86–1.16)	0.99 (0.86–1.16)	1.01 (0.87–1.17)
Heart disease					
Low physical activity	885	1.00 (Ref)	1.00 (Ref)	1.00 (Ref)	1.00 (Ref)
Moderate physical activity	1203	0.79 (0.72–0.88)	0.90 (0.81–1.00)	0.84 (0.76–0.94)	0.86 (0.77–0.95)
High physical activity	2015	0.59 (0.54–0.64)	0.73 (0.66–0.80)	0.72 (0.66–0.80)	0.75 (0.68–0.82)
Stroke					
Low physical activity	406	1.00(Ref)	1.00(Ref)	1.00(Ref)	1.00(Ref)
Moderate physical activity	469	0.69 (0.60–0.79)	0.79 (0.68–0.91)	0.78 (0.68–0.91)	0.80 (0.69–0.92)
High physical activity	650	0.43 (0.38–0.49)	0.55 (0.48–0.63)	0.55 (0.48–0.63)	0.58 (0.50–0.66)
Kidney disease					
Low physical activity	361	1.00 (Ref)	1.00 (Ref)	1.00 (Ref)	1.00 (Ref)
Moderate physical activity	613	1.06 (0.92–1.22)	1.12 (0.97–1.29)	1.08 (0.94–1.25)	1.10 (0.96–1.27)
High physical activity	1170	0.94 (0.83–1.07)	1.04 (0.92–1.18)	1.03 (0.90–1.17)	1.06 (0.93–1.21)
Stomach disease					
Low physical activity	998	1.00 (Ref)	1.00 (Ref)	1.00 (Ref)	1.00 (Ref)
Moderate physical activity	1658	1.05 (0.95–1.15)	1.07 (0.97–1.18)	1.12 (1.01–1.23)	1.13 (1.02–1.25)
High physical activity	3489	1.04 (0.95–1.13)	1.07 (0.98–1.17)	1.08 (0.99–1.18)	1.10 (1.00–1.20)
Emotional and mental issues					
Low physical activity	165	1.00 (Ref)	1.00 (Ref)	1.00 (Ref)	1.00 (Ref)
Moderate physical activity	162	0.60 (0.48–0.74)	0.62 (0.50–0.78)	0.66 (0.53–0.83)	0.68 (0.54–0.85)
High physical activity	246	0.42 (0.35–0.52)	0.45 (0.36–0.55)	0.46 (0.37–0.57)	0.48 (0.39–0.59)
Memory-related diseases					
Low physical activity	201	1.00 (Ref)	1.00 (Ref)	1.00 (Ref)	1.00 (Ref)
Moderate physical activity	225	0.68 (0.56–0.83)	0.84 (0.68–1.02)	0.79 (0.65–0.97)	0.81 (0.66–0.99)
High physical activity	331	0.47 (0.39–0.56)	0.67 (0.56–0.81)	0.67 (0.55–0.81)	0.70 (0.58–0.85)
Parkinson’s disease					
Low physical activity	111	1.00 (Ref)	1.00 (Ref)	1.00 (Ref)	1.00 (Ref)
Moderate physical activity	137	0.76 (0.59–0.98)	0.87 (0.67–1.13)	0.76 (0.59–0.99)	0.78 (0.60–1.01)
High physical activity	237	0.62 (0.49–0.78)	0.78 (0.62–1.00)	0.78 (0.62–1.00)	0.81 (0.64–1.04)
Arthritis or rheumatism					
Low physical activity	1310	1.00 (Ref)	1.00 (Ref)	1.00 (Ref)	1.00 (Ref)
Moderate physical activity	1908	0.84 (0.77–0.92)	0.92 (0.84–1.02)	1.02 (0.92–1.12)	1.02 (0.93–1.12)
High physical activity	4353	0.95 (0.88–1.04)	1.12 (1.03–1.22)	1.13 (1.04–1.23)	1.14 (1.04–1.24)
Asthma					
Low physical activity	323	1.00 (Ref)	1.00 (Ref)	1.00 (Ref)	1.00 (Ref)
Moderate physical activity	312	0.57 (0.49–0.67)	0.65 (0.55–0.76)	0.68 (0.58–0.81)	0.70 (0.59–0.83)
High physical activity	561	0.48 (0.42–0.56)	0.60 (0.52–0.70)	0.61 (0.52–0.70)	0.64 (0.55–0.74)

^a^Low physical activity =  < 600 MET × min per week. Moderate physical activity = 600–3000 MET × min per week.

High physical activity =  > 3000 MET × min per week.

^b^Model 1 is the unadjusted association. Model 2 is adjusted for age and gender. Model 3 is additionally adjusted for marital status, PCE, education level, and residential area. Model 4 is additionally adjusted sleep, drinking status, and smoking status.

To understand how higher levels of physical activity are associated with the prevalence of chronic diseases, we used the moderate physical activity group as a reference in a multivariate logistic regression analysis to compare the differences in 15 chronic diseases with the high physical activity group. The fully adjusted model results showed that the high physical activity group had a further reduced prevalence for seven diseases, with the largest decrease observed for cancer and memory-related disease (OR = 0.71, 95% CI: 0.58–0.87). However, the prevalence of arthritis and rheumatism also increased (OR = 1.11, 95% CI: 1.04–1.20), as shown in Fig. 1.

## Discussion

This study conducted a cross-sectional analysis using a nationally representative sample to explore the associations between physical activity levels and the prevalence of chronic diseases among middle-aged and elderly populations in urban and rural China. We found that over one-sixth of the middle-aged and elderly did not meet the recommended levels of physical activity(≥ 600 MET × min per week), consistent with the World Health Organization’s physical activity guidelines report and similar to previous domestic studies in China^9,14^. Our study also found that the proportion of those engaging in physical activity and meeting the guidelines decreased with age, confirming that this phenomenon also exists in the middle-aged and elderly population^24^. Additionally, as education levels increased, so did the proportion of people meeting the physical activity guidelines, with urban populations more likely to meet the guidelines than rural and suburban populations. Current smokers had a higher proportion than Never smokers and Quit smoking, and married individuals had a higher proportion than other groups, except for divorced individuals^25,26^. These findings are consistent with previous research, indicating a general trend. In our study, man were more likely than women to achieve higher physical activity equivalents. A study using the UK Biobank database found that men spent more time than women in low-level or sedentary physical activity, while women spent more time in moderate-level activity^27^. Another study from the UK came to the exact opposite conclusion^28^. Therefore, gender differences in physical activity may be affected by age, region and other conditions. Interestingly, we found that drinkers had higher levels of physical activity than non-drinkers, which may be related to the greater number of drinkers engaged in manual labor. Previous research has shown that people working in manual skills occupations and in the construction industry are more likely to be dependent on alcohol^29,30^.

In recent years, research on physical activity has mainly focused on the associations between guideline-compliant physical activity and the prevalence of certain chronic diseases, as well as the optimal time periods for physical activity and its health-promoting effects^25,31^. We noted that few studies have examined the impact of different levels of physical activity on the prevalence of multiple chronic diseases, especially in the middle-aged and elderly population. Our study explored the associations between different levels of physical activity and 15 common chronic diseases. Initially, using the low physical activity group as a reference, we found that the odds ratio (OR) and 95% confidence interval (CI) for four chronic diseases were less than 1 for the moderate physical activity group. This suggests that moderate physical activity may be associated with a lower prevalence of these four chronic diseases. Similarly, high physical activity may be associated with a lower prevalence of ten chronic diseases. Further, using the moderate physical activity group as a reference, we found that the high physical activity group still had a lower prevalence of nine chronic diseases. The results of the PURE study, which included 130,000 people from 17 countries, showed that the more physical activity, the lower the prevalence of hypertension and diabetes. Compared to moderate physical activity level (600–3000 MET-min/per week), the high physical activity group (> 3000 MET-min/per week) had a lower all-cause mortality rate and a lower prevalence of major cardiovascular diseases^32^. In our study, we also found an association between higher levels of physical activity and a lower prevalence of certain diseases, which may be related to the protective effects of physical activity against chronic diseases. In a systematic review, the authors considered physical activity as an exercise therapy for 26 chronic diseases and summarized the possible biological mechanisms. The mechanisms by which physical activity prevents cancer may include reducing tumor growth through various mechanisms, including (a) vascularization and blood perfusion, (b) immune function, (c) tumor metabolism, and (d) interactions between muscles and cancer. The blood pressure-lowering mechanisms of physical activity include neurohormonal, vascular, and structural adaptations. Anti-allergic effects include reduced vasoconstriction induced by the sympathetic nervous system and lower catecholamine levels in a healthy state. The risk of type 2 diabetes is reduced by increasing insulin sensitivity in trained muscles and muscle glucose uptake induced by muscle contractions. At the cellular level, aerobic exercise has been shown to have various anti-atherosclerotic benefits, including lowering serum triglycerides, increasing high-density lipoprotein, and lowering low-density lipoprotein, thereby reducing the risk of chronic diseases^10^.

In past research, physical activity has been identified as a protective factor for most chronic diseases, but the association between some chronic diseases and physical activity has not been consistent. A study using data from the Spanish National Health Survey (2017) showed that weekly physical activity levels below 600 MET-min were significantly associated with the prevalence of 19 chronic diseases, while the lowest prevalence of chronic diseases was observed in people who exercised more than 1200 MET-minutes per week^33^. However, this trend was not observed in chronic allergies and prostate issues. In our study, we found that after adjusting for relevant covariates, the high physical activity group did not show the expected decrease in the prevalence of chronic lung diseases, liver diseases, kidney diseases, stomach diseases, Parkinson’s disease, and arthritis or rheumatism. Moreover, in model 4, which fully adjusted for confounding factors, we found that compared to the low physical activity group, the moderate physical activity group had an increased prevalence of stomach diseases, and the high physical activity group had an increased prevalence of stomach diseases and arthritis or rheumatism. In studies on physical activity and stomach diseases, previous research has shown that regular physical activity can reduce the prevalence of Gastroesophageal reflux disease (GERD) and has recommended increasing physical activity levels as an effective measure to prevent GERD^34^. Recently published Mendelian randomization studies have similarly indicated that moderate-to-vigorous physical activity (MVPA) can reduce the risk of eight gastrointestinal diseases, suggesting a potential causal relationship between MVPA and certain gastrointestinal conditions^35^. However, in our study, higher levels of physical activity were significantly associated with a higher prevalence of stomach diseases. This may be due to the higher prevalence of stomach diseases in our sample, with many participants coming from rural areas and engaging in high-intensity physical labor. Previous research has shown that fatigue is associated with a higher prevalence of Ulcerative colitis (UC)^36^, and a prospective cohort study has indicated that heavy physical labor increases the risk of Barrett’s esophagus^37^. In research concerning physical activity and arthritis, a recent cross-sectional study showed that the prevalence of arthritis in middle-aged and elderly patients was related to metabolic syndrome but not to physical activity^38^. Research indicates that individuals with chronic diseases find it more challenging to achieve higher levels of physical activity. This is due to various limitations they encounter while engaging in physical activities, such as concerns about exacerbating inflammation and clinical recommendations to avoid strenuous exercise^39^. Another prospective cohort study found that the risk of rheumatoid arthritis continued to decrease with increasing weekly physical activity time, with the highest risk reduction of up to 33% when weekly physical activity time was seven hours or more^40^. The discrepancy with previous studies may be due to different types of physical activity. Studies have shown that mechanical heavy physical activity may increase the prevalence of arthritis^41^, and many people in our study were from rural areas, inevitably engaging in heavy physical labor such as farming. Additionally, related physical activity research has shown that exercise can alleviate pain and improve physical function in patients with different types of arthritis^42^, reducing cartilage damage^43^. Therefore, we still recommend that middle-aged and elderly people with arthritis engage in physical activity under scientific guidance, participating more in swimming and other joint-friendly exercises, as swimming puts less compression on the joints^44^.

The key advantage of this study is the use of nationally representative data to analyze the associations between different levels of physical activity and the prevalence of 15 common chronic diseases, whereas previous studies focused more on a single chronic disease. However, our study has some limitations worth noting. First, as it relies on self-reported questionnaire data rather than objective measurement tools (such as accelerometers or pedometers), our results may be subject to memory bias and social desirability bias, which could lead to overestimation or underestimation of physical activity levels^45^. However, in the field of public health, standardized questionnaires are often the preferred method for measuring physical activity due to their low cost and certain reliability and validity^46^. Second, due to the cross-sectional study design, we cannot determine the causal relationship between physical activity and chronic diseases. Although we observed a significant association between the two, this design limits our interpretation of whether physical activity directly leads to a reduced prevalence of chronic diseases. Therefore, future longitudinal or experimental studies are needed to further explore this relationship.

## Conclusion

Research indicates that individuals with moderate (600–3000 MET-minutes per week) and high levels (> 3000 MET-minutes per week) of physical activity have lower prevalence rates of certain diseases compared to those with low levels (< 600 MET-minutes per week) of physical activity. Additionally, individuals with high levels of physical activity exhibit a further reduction in disease prevalence compared to those with moderate levels of physical activity. Currently, there is limited causal evidence regarding this association, and further research is needed to elucidate this relationship.

## Electronic supplementary material

Below is the link to the electronic supplementary material.

Supplementary Material 1

Supplementary Material 2

## Data Availability

The datasets used and analysed during the current study available from the corresponding author on reasonable request. The data used in the study can be obtained from https://charls.pku.edu.cn/.
