# Supplementary material for: A cross-sectional study of physical activity and chronic diseases among middle-aged and elderly in China
Source: Sci Rep. 2024 Dec 28;14:30701. doi: 10.1038/s41598-024-78360-z (PMC11680886; doi:10.1038/s41598-024-78360-z)
Supplement: Supplementary file 1 — Supplementary Material 1 [file 41598_2024_78360_MOESM1_ESM.pdf]

Machine Translated by Google

China Health and Retirement Longitudinal Study (CHARLS)

Fifth round (2020) follow-up questionnaire

Version Number: 20231106

November 2023

National School of Development, Peking University

Center for Chinese Social Science Survey, Peking University

Machine Translated by Google

This page intentionally left blank

Machine Translated by Google

Table of contents

CV Filter Questionnaire 1

CV1. Two or one person has visited the household 2

CV2. Marriage and separation of two-person households 2

CV3. Marital status of the main respondent 2

Auxiliary variable definition 3

B Basic information 7

B. Proxy mode confirmation 8

BA. Basic Information 8

Auxiliary Variable Definitions 10

C. Family information 13

C. Agency mode confirmation 14

CA. Children's Information 14

CB. Household member information 17

CC. Family contact during the Spring Festival 18

Auxiliary variable definition 20

D Health status and function 25

D. Proxy mode confirmation 26

DA. Health status (I) 26

DA. Health status (II) 28

DA. Health status (III) 29

DB. Physical impairment and its helpers (I) 32

DB. Physical dysfunction and its helpers (II) 35

DB. Physical impairment and its helpers (III) 36

DC. Cognition and depression (I) 38

DC. Cognition and Depression (II) 40

Auxiliary Variable Definitions 45

F Work and retirement 51

F. Agency model confirmation 52

Machine Translated by Google

FA. Job Overview (I) 52
FA. Job Overview (II) 53
FB. Self-employed agricultural work 54
FC. Employment (I) 55
FC. Employment (II) 57
FD. Nonfarm self-employment 59
FE. Non-main work 60
FF. Job search and employment 60
FG. Working during a pandemic 61
FH. Retirement Procedures 62
Auxiliary Variable Definitions 63

G. Income and Expenditure **67**

G1 Household income and expenditure 68
G1. Proxy mode confirmation 68
GB. Income of other household members 68
GC. Household agricultural income 69
GD. Income from self-employment and private enterprise 71
GE. Household public transfer income 72
GF. Household living expenses 74
I. Housing conditions 77
G2 Personal income 81
G2. Proxy mode confirmation 81
GA. Personal income 81
Auxiliary variable definitions 83

V Epidemic **87**

V. Agency model confirmed 88
VA. Disease Awareness 88
VB. Personal illness and isolation 89
VC. Personal activities during the epidemic 91
VD. Control of residence during epidemic 94
period . Definition of auxiliary variables 95

EX Exit **97**

Questionnaire EXB. Basic Information 98
EXC. Family 100
EXD. Health status and function (I) 101
EXD. Health status and function (II) 104

Machine Translated by Google

EXD. Health status and function (III) 107
EXE. Health Care and Insurance (I) 109
EXE. Health Care and Insurance (II) 111
EXF. Work and retirement 113
EXFN. Pension 114
EXG. Income, Expenses and Assets 114
EXK. Funeral 118
EXV. Epidemic related 119
VA. Cause of death analysis 121
Auxiliary variable definitions 122

Appendix Function **131**

Description A. Function in the question 132
B. Functions in auxiliary variables 132

Machine Translated by Google

This page intentionally left blank

Machine Translated by Google

CV Filter Questionnaire

Machine Translated by Google

CV1. Two or one person has visited the household

CV001Who answered the filter questionnaire in the following list?

1. [ZRName1]
2. [ZRName2]
3. Agent, name **(CV001_1) ,** who is [ZRName1] ? ________ **(CV001_2)**

CV002 Is [ZRName1] still alive?

[Interviewer's note: If the person who answered the filter questionnaire is [ZRName1] **,** there is no need to ask this question. Just select "Alive". Use 4 digits to indicate the year.]

1. Still alive
2. Died, the time of death is [hc([2011, 2020], , ÿ1)] **(CV002_2)** , ÿ), sc([ZIWYear, 2020], , ÿ)] **(CV002_1)**

Year ________ months [hc([1, 12], ________ [hc([1, 31], , ÿ1)] **(CV002_3)** day

CV003 Is [ZRName2] still alive?

[Interviewer's note: If the person who answered the filter questionnaire is [ZRName2] **,** there is no need to ask this question, just select "Alive". Use 4 digits to indicate the year.]

1. Still alive
2. Died, the time of death is [hc([2011, 2020], , ÿ), sc([ZIWYear, 2020], , ÿ)] **(CV003_1)**

Year ________ [hc([1, 12], , ÿ1)] **(CV003_2)** month ________ [hc([1, 31], , ÿ1)] **(CV003_3)** day

CV2. Marriage and separation of two-person households

CV004 Are [ZRName1] and [ZRName2] still married or living together?

1. [ZRName1] and [ZRName2] are still married or living together
2. [ZRName1] and [ZRName2] are divorced
3. [ZRName1] and [ZRName2] have been separated for a long time and do not expect to live together as a couple in the future

CV005Whether [ZRName1] or [ZRName2] , whose situation are you more familiar with?

1. [ZRName1]
2. [ZRName2]

CV3. Marital status of the main interviewee

CV006 [XMainR] What is your current marital status?

1. Married and living with spouse
2. Married, but not living with spouse temporarily due to work or other reasons
3. Separation and no longer living together as a spouse
4. Divorce
5. Widowed
6. Never Married

CV007 [XMainR] is currently without a spouse, or is separated from a married spouse and no longer living together as a spouse, then [XMainR]

Did you live with your partner as a spouse before?

1. Yes, name **(CV007_1)** ________
2. No

Machine Translated by Google

CV008 What is the name of [XMainR] 's spouse? _______

Auxiliary variable definition

XRLive1Interviewee 1 Living or dead

```
if (equal("CV002", "1"))
      { add("XRLive1", "1")

} if (equal("CV002", "2"))
      { add("XRLive1", "2")
}
```

XRLive2 Interviewee 2 Living or deceased

```
if (equal("CV003", "1"))
      { add("XRLive2", "1")

} if (equal("CV003", "2"))
      { add("XRLive2", "2")
}
```

XBothAlive respondents 1 and 2 alive or dead

```
if (equal("CV002","1") && (equal("CV003","1")) {
      add("XBothAlive", "1")

} if (equal("CV002","2") && (equal("CV003","2")) {
      add("XBothAlive", "2")

} if (equal("CV002","1") && (equal("CV003","2")) {
      add("XBothAlive", "3")

} if (equal("CV002","2") && (equal("CV003","1")) {
      add("XBothAlive", "4")
}
```

XMaritalStatusDual Household Marriage

```
if (equal("CV004", "1")) {
      add("XMaritalStatus", "1")

} if (equal("CV004", "2") || equal("CV004", "3"))
      { add("XMaritalStatus", "2")
}
```

XMainR Main interviewee name

```
if (equal("CV_HType", "1") && equal("XBothAlive", "3"))
      { add("XMainR", value("ZRName1"))
      add("XRType1", "1")

} if (equal("CV_HType", "1") && equal("XBothAlive", "4"))
      { add("XMainR", value("ZRName2"))
      add("XRType2", "1")

} if (equal("CV_HType", "2") && equal(" XRLive1", "1"))
      { add("XMainR", "ZRName1")
      add("XRType1", "1")
}

if (equal("XMaritalStatus", "1"))
      { add("XMainR", "ZRName1")
      add("XRType1", "1")
      add("XMainRS", "ZRName2")
      add("XRType2", "1")
}

if (((equal("CV004", "2") || equal("CV004", "3")) && equal("CV001", "1")) || ((equal("CV004",
      " 2") || equal("CV004", "3")) && equal("CV001", "3") && equal("CV005", "1"))) {
```

Machine Translated by Google


```
        add("XMainR", "ZRName1")
        add("XRType1", "1")
        add("XRSplited2", "1") }
    else
        { add("XRSplited2", "0")
    }

    if (((equal("CV004", "2") || equal("CV004", "3")) && equal("CV001", "2")) || ((equal("CV004",
        " 2") || equal("CV004", "3")) && equal("CV001", "3") && equal("CV005", "2"))) { add("XMainR", "ZRName2" )
        add("XRType2", "1")
        add("XRSplited1",
        "1") } else

    { add("XRSplited1", "0")
    }
```

XMainRS Spouse Name of the Main Respondent

see above

XRType1Interviewee 1 Type

see above

XRType2Interviewee 2 Type

see above

XRSplited1Respondent 1's new households

```
    if (((equal("CV004", "2") || equal("CV004", "3")) && equal("CV001", "2")) || ((equal("CV004", " 2") || ÿ equal("CV004",
     "3")) && equal("CV001", "3") && equal("CV005", "2"))) {
        add("XMainR", "ZRName2")
        add("XRType2", "1")
        add("XRSplited1", "1") }
    else
        { add("XRSplited1", "0")
    }
```

XRSplited2 New households of respondent 2

```
    if (((equal("CV004", "2") || equal("CV004", "3")) && equal("CV001", "1")) || ((equal("CV004", " 2") || ÿ equal("CV004",
     "3")) && equal("CV001", "3") && equal("CV005", "1"))) {
        add("XMainR", "ZRName1")
        add("XRType1", "1")
        add("XRSplited2", "1") }
    else
        { add("XRSplited2", "0")
    }
```

XRDeathYear1Interviewee 1 Death Year

add("XRDeathYear1", value("CV002_1"))

XRDeathMonth1Interviewee 1 Death month

add("XRDeathMonth1", value("CV002_2"))

XRDeathDate1Interviewee 1 Death date

add("XRDeathDate1", value("CV002_3"))

XRDeathYear2Interviewee 2 Death Year

add("XRDeathYear2", value("CV003_1"))

XRDeathMonth2Interviewee 2 Death Month

add("XRDeathMonth2", value("CV003_2"))

XRDeathDate2Interviewee 2 Death date

Machine Translated by Google

add("XRDeathDate2", value("CV003_3"))

ZRName1Interviewee 1 Name

ZRName2Interviewee 2 Name

ZIWTime Last visit time

Machine Translated by Google

This page intentionally left blank

Machine Translated by Google

B. Basic Information

Machine Translated by Google

B. Proxy Mode Confirmation

proxy_2 Interviewer record: For the basic information module, is the proxy questionnaire mode used?

1. Yes 2.

No

BA. Basic Information

BA001 Interviewer records the gender of [XRName] 1.

Male 2.

Female

BA002 The gender of [XRName] we recorded last time was [XR18Gender] , this time your gender is [XR20Gender]

Please confirm the gender of [XRName] again. 1.

Male 2.

Female

BA003 The real Gregorian calendar date of birth of [XRName] is [hc([1900, 2000], , ÿ)] **(BA003_1)** year [hc([, ÿ1)] **(BA003_2)** month , ÿ1)] **(BA003_3)** day1 , 12],

unknown [Interviewer's note: The year is mandatory an [hc([1, 31], expressed in 4 digits. If the month and day are

or forgotten, "-1" can be entered.]

BA004 No need to ask questions. Please record the visit address directly:

________ **(BA004_1)** Province/City/District/County

________ **(BA004_2)** Township/town/street/village/community

________ **(BA004_3)** Residential area/building number/unit/house number

BA005 No need to ask questions. Please directly record the type of place visited.

1. Family residence

2. Workplace 3.

Others, please specify: ________ **(BA005_1)**

BA006 Where does [XRName] live now? Excluding temporary business trips, tourism, and temporary one or two-day visits to relatives

and friends. 1. Visited place: [XRSurveyAdd]

2. Non-visited place: Mainland China **(BA006_1)** Province/City/District/County ________ **(BA006_2)** Township/town/street/village/community

________ **(BA006_3)** Residential area/building number/unit/house

number 3. Hong

Kong, China 4.

Macau, China 5.

Taiwan, China 6. Overseas: **(BA006_4)**

BA007 What is the type of residence of [XRName] at residential address [XRResidenceFull] ? 1. Family

home 2. Nursing

home or other nursing home 3. Hospital 4.

Other, please

specify **(BA007_1)** ________

BA008 [XRResidenceQuestion] ?

Machine Translated by Google

1. City or town center 2.

Urban-rural or town-village

combined

area 3. Rural area 4. Special area

BA009 What is [XRName]' s current household registration type? 1.

Agricultural 2.

Non-agricultural 3.

Unified resident household

registration 4. No household registration

BA010 What is the highest level of education [XRName] has currently obtained (excluding adult education)?

1. Uneducated (illiterate) 2. Did not finish primary school

3. Graduated from private school 4.

Graduated from primary school

5. Graduated from junior high

school 6. Graduated from high

school 7. Graduated from

technical secondary school (including secondary normal school, vocational high school) 8.

Graduated from junior college

9. Graduated from

undergraduate school 10.

Graduated from master's degree 11. Graduated from doctorate

BA010_1 Is [XRName] literate? 1. Yes 2. No

BA011 What is [XRName]'s current marital status? 1.

Married and living with spouse 2.

Married, but not living with spouse temporarily due to work or other reasons 3. Separated

(no longer living together as spouse) 4. Divorced 5.

Widowed 6.

Never married

BA012 Does [XRName] currently live with a partner as a spouse (cohabiting)?

1. Yes 2.

No

BA013 In the past year, how long did [XRName] live with his/her spouse or partner? [hc([0, 12], ______ , ÿ)]

Month

BA014 How often is [XRName] 's social pension insurance paid out?

[Interviewer's note: If you do not know the distribution frequency, but only know the collection frequency, then record the shortest time for collection and note the collection frequency.]

1. One month

2. One quarter 3.

Half a year

Machine Translated by Google

4. One year

5. More than one year, which is [hc((1, 50), , ÿ)] **(BA014_1)** years 6. Participated in pension insurance, but not yet reached the age of receiving benefits 7. Did not participate in any pension insurance

BA015 Which social pension insurance does [XRName] participate in?

[Interviewer's note: If the interviewee does not know the type of insurance he/she is participating in, he/she can ask his/her children, village cadres, etc. who may know.] 1. Government agency/institution pension insurance 2. Enterprise employee pension insurance 3. Urban resident pension insurance 4. New rural resident social pension insurance (New Rural Pension Insurance) 5. Urban and rural resident pension insurance 6. Others, please specify **(BA015_1)**

BA016 How much does [XRName] 's social medical insurance cost per year?

[Interviewer's note: After the follow-up question, if the respondent does not know the amount of social medical insurance payment, he/she can fill in "-1". If the payment amount varies from year to year, record the most recent payment amount.]

1. Annual payment [hc((0, 100000], 2. Have medical , ÿ1)] **(BA016_1)** insurance, but no payment required 3. Not enrolled in any medical insurance

BA017 Which social medical insurance does [XRName] participate in?

[Interviewer's note: If the interviewee does not know the type of insurance he/she is participating in, he/she can ask his/her children, village cadres, etc. who may know.]

1. Urban employee medical insurance (medical insurance) 2. Urban and rural residents medical insurance (combined urban residents and new rural cooperative medical insurance) 3. Urban residents medical insurance 4. New rural cooperative medical insurance (cooperative medical insurance) 5. Public medical insurance 6. Others, please specify **(BA017_1)**

BA018 In the first half of this year, how many days did [XRName] live alone? [hc([0, 182], , ÿ)]

BA019 In the first half of this year, how many days did [XRName] live with only his spouse/partner (i.e., no one else except his spouse)? [hc([0, 182], live)? , ÿ)]

BA020In the first half of this year, [XRName] did not live with [XRLiveCovid] for [DayNumber] days . To what extent was this caused by the COVID-19 pandemic? (Express as a percentage) [hc([0, 100], , ÿ)] % [Interviewer Note: This question does not ask to what extent the pandemic has affected the number of days the respondent lives alone or with his/her spouse, but asks to what extent the number of days the respondent lives alone or with his/her spouse is caused by the pandemic.]

Auxiliary variable definition

XR20GenderInterviewer records gender this time

```
if (equal("BA001", "1"))
    { add("XR20Gender", "Male")

} if (equal("BA001", "2"))
    { add("XR20Gender", "female")
}
```

Machine Translated by Google

XR18Gender 18-year survey records gender

```
if (equal("ZRGender", "1"))
    { add("XR18Gender", "Male")

} if (equal("ZRGender", "2"))
    { add("XR18Gender", "Female")
}
```

XRGender gender (after confirmation)

```
if (!empty("ZRGender") && equal("BA001", value("ZRGender"))) {
    add("XRGender", value("BA001"))

} if (!empty("ZRGender") && !equal("BA001", value("ZRGender"))) {
    add("XRGender", value("BA002"))

} if (empty("ZRGender"))
    { add("XRGender", value("BA001"))
}
```

XRAge age

```
if (empty("ZRBirthYear") || equal("XRType", "2"))
    { add("XRAge", 2020 - value("BA003_1"))

} if (equal("XRType", "1") && !empty("ZRBirthYear"))
    { add("XRAge", 2020 - value("ZRBirthYear"))
}
```

XRResidenceFull five-level address: provinces, cities, counties, villages, Hong Kong, Macau, Taiwan, and abroad

```
if (equal("BA006", "1"))
    { add("XRResidenceFull",value("BA004_1")+value("BA004_2"))

} if (equal("BA006", "2"))
    { add("XRResidenceFull",value("BA006_1")+value("BA006_2"))

} if (equal("BA006", "3"))
    { add("XRResidenceFull", "Hong Kong")

} if (equal("BA006", "4"))
    { add("XRResidenceFull", "Macao, China")

} if (equal("BA006", "5"))
    { add("XRResidenceFull", "Taiwan")

} if (equal("BA006", "6")) {
    add("XRResidenceFull", "Overseas:"+value("BA006_4"))
}
```

XRResidenceCounty first three levels of address: province, city, county

```
if (equal("BA006", "1"))
    { add("XRResidenceCounty",value("BA004_1"))

} if (equal("BA006", "2"))
    { add("XRResidenceCounty",value("BA006_1"))
}
```

XRResidenceCommunity 's third-level address: County and Village

```
if (equal("BA006", "1"))
    { add("XRResidenceCommunity",value("BA004_2"))

} if (equal("BA006", "2"))
    { add("XRResidenceCommunity",value("BA006_2"))
}
```

XRSurveyAdd access address (including house number)

```
add("XRSurveyAdd", value("BA004_1")+value("BA004_2")+value("BA004_3"))
```

Machine Translated by Google

XRResidenceSurveyHomeWhether the current residence is the place to be visited and is it a family residence

```
if (equal("BA006", "1") && equal("BA005", "1"))
    { add("XRResidenceSurveyHome", "1")

} if ((equal("BA006", "1") && !equal("BA005", "1")) || equal("BA006", "2") || equal("BA006", "3" ) || ÿ equal("BA006", "4") ||
 equal("BA006", "5") || equal("BA006", "6") ) { add("XRResidenceSurveyHome", "2")

}
```

XRResidenceQuestion da008 (General Residence Urban-Rural Category) Question stem different questions

```
if (equal("BA006", "1"))
    { add("XRResidenceQuestion", "Visitors can fill in the form by themselves"+value("XRSurveyAddFull")+"Is it rural or urban")

} if (equal("BA006", "2")) {
    add("XRResidenceQuestion", ÿ
     value("XRName")+"In residence"+value("XROtherInlandFull")+"Do you mainly live in rural areas or cities when you live")
}
```

XRSurveyAddFull survey address: fifth-level address (excluding house number)

```
add("XRSurveyAddFull", value("BA004_1")+value("BA004_2"))
```

XROtherInlandFull Current address: Non-visited place, other addresses in mainland China Level 5 address (excluding house number)

```
if (equal("BA006", "2"))
    { add("XROtherInlandFull", value("BA006_1")+value("BA006_2"))
}
```

Does **XRPartner** have a spouse/partner?

```
if ((equal("BA011", "1") || equal("BA011", "2")) || ((equal("BA011", "3") || equal("BA011", "4 ") || ÿ equal("BA011", "5") ||

    equal("BA011", "6")) && equal("BA012", "1")))
    { add("XRPartner", "1")

} if ((equal("BA011", "3") || equal("BA011", " 4") || equal( "BA011", " 5") || equal("BA011", "6") ) && ÿ equal("BA012", "2"))
 { add("XRPartner", "2")

}
```

XRLiveCovid does not live with other people. To what extent is it caused by the epidemic? Question

```
if (equal("XRPartner", "1"))
    { add("XRLiveCovid", "Anyone other than spouse/partner")

} if (equal("XRPartner", "2")) {
    add("XRLiveCovid", "Anyone else")
}
```

DayNumberNumber of days not living with anyone else (other than spouse/partner)

```
if (equal("XRPartner", "1"))
    { add("DayNumber", value("BA018")+value("BA019"))

} if (equal("XRPartner", "2")) {
    add("DayNumber", value("BA018"))
}
```

XRNameInterviewee Name

Machine Translated by Google

C. Family Information

Machine Translated by Google

C. Proxy mode confirmation

Proxy_3 Interviewer record: For the work module, do you use the proxy questionnaire mode?

1. Yes 2. No

CA. Children's Information

CA001 Next we will ask some questions about [XMainR] and [XMainRS] family members, including children, household members, Wait, who knows more about this, [XMainR] or [XMainRS] ?

1. [XMainR]
2. [XMainRS]

[Quote: Now I would like to ask some questions about [XFamilyR] 's children]

CA002[ ] Is [ZChildName[ ]] (gender: [XChildGenderDis[ ]]) still alive?

1. Yes
2. No

When did **CA003[ ]** [ZChildName[ ]] die? [hc([2011, 2020], , ÿ), sc([ZIWYear, 2020], , ÿ)] **(CA003_2[i])** months , ÿ1)] **(CA0**

, ÿ)] **(CA003_1[i])ÿ03_3[i])ÿ** ________ [hc([1, 12], ________ [hc([1, 31],

[Interviewer's

note: The year and month of death are mandatory and are used to generate the logic of the questionnaire. For the specific day of death, if the respondent refuses to answer or forgets, please fill in "-1"]

CA004[ ] What was the main cause of [ZChildName[ ]]'s death? ________

[Interviewer's note: If the death is due to disease, please specify the cause in detail. For example, if the death is due to cancer, please indicate the type of cancer (stomach cancer, lung cancer, etc.); if the death is due to infectious disease, please indicate the type of infectious disease (tuberculosis, dysentery); if the death is due to an accident, please indicate the type of accident, such as Car accidents, fires, accidental poisoning, etc.]

CA005[ ] [XChildPanAliveName[ ]] When was he born? [hc([1910,ZIWYear], , ÿ), sc([1940, ZIWYear], , ÿ)] Years **[Interviewer's note: Use** 4 digits to represent the year. If the respondent cannot remember the year of birth, the year of birth can be estimated based on the current age of the child, or the year of death, the age of the respondent when the child was born, etc.]

CA006[ ] [XChildPanAliveName[ ]]'s gender? 1. Male 2. Female

CA007[ ] Excluding adult education, what is the highest level of education of [XChildPanAliveName[ ]]? 1. No formal education 2. Did not finish primary school 3. Private school 4. Primary school graduate 5. Junior high school graduate 6. Senior high school graduate 7. Technical secondary school (including secondary normal school, vocational high school) graduate

Machine Translated by Google

8. College graduates

9. Bachelor's degrees

10. Master's degrees

11. Doctoral degrees

997. Don't know999.

Refuse to answer

CA008[ ] [XChildAliveName[ ]] Is he currently working or attending school? Jobs include farming, wage work, working as a helper in a family business or in a private enterprise without pay

1. Working 2.

Going to

school 3. Working and going

to school 4. Neither working nor going to school, please indicate what you are doing now **(CA008_1[i])** 997. Don't

know 999. Refuse to

answer

CA009[ ] [XChildAliveName[ ]] What kind of job is this? In other words, what does [XChildAliveName[ ]] do now?

________ (Example 1: Restaurant pastry chef; Example 2: Production line winding worker)

CA010[ ] [XChildPanAliveName[ ]] What is your current marital status? 1. Married and living

with your spouse 2. Married, but not living

with your spouse for work or other reasons 3. Separated (no longer living together as a couple)

4. Divorced 5. Widowed 6. Never married 997. Don't know

999. Refuse

to answer

CA012[ ] In the past year, [XChildCoupleDis[ ]]'s total income last year belongs to which of the following categories? 1. 0 No

income 2. Less than

2,000 yuan 3. Between

2,000 and 5,000 yuan 4. Between

5,000 and 10,000 yuan 5.

Between 10,000 and 20,000

yuan 6. Between 20,000 and

30,000 yuan 7. Between 30,000

and 50,000 yuan 8. Between

50,000 and 100,000 yuan 9. Between

100,000 and 150,000 yuan 10. Between

150,000 and 200,000 yuan 11. Between

200,000 and 300,000

yuan 12. More than

300,000 yuan 997. Don't know 999. Refuse to answer

CA013[ ] How is [XChildPanAliveName[ ]]'s health now? Very good, good, fair, poor, or very poor

not good?

Machine Translated by Google

1. Very
good 2.
Good 3.
Average 4.
Not good 5.
Very bad 997.
Don't know 999. Refuse to answer

[Quote: Next we will ask about [XFamilyR]' s interactions with his children and their financial support to each other.]

CA014[ ] In the past year, how long did [XChildPanAliveName[ ]] live with [XFamilyRAndS] ? [hc , ÿ)] months ________

([0, 12],

[Interviewer's note: Short visits to relatives do not count as living together; if you do not live together, please fill in 0; if you always live together, please fill in 12]

CA015[ ] When [XFamilyRAndS] and [XChildPanAliveName[ ]] do not live together, how long does [XFamilyRAndS]

How often do you see [XChildPanAliveName[ ]]? 1. Almost
every day 2. 2-3
times a week 3. Once
a week 4. Once
every two weeks 5. Once
a month 6. Once
every three months 7.
Once every six
months 8. Once a
year 9. Almost never 10.
Other

CA016[ ] When [XFamilyRAndS] and [XChildPanAliveName[ ]] are not living together, how often does [XFamilyRAndS] contact [XChildPanAliveName[ ]] by phone, text message, WeChat, letter, or email?

1. Almost every day
2. 2-3 times a week
3. Once a week
4. Once every half month
5. Once a month
6. Once every three
months 7. Once
every six months
8. Once a year 9. Almost
never 10. Other

[Quote: Sometimes, families help each other in many ways, and each form of help is important. So, next we want to know whether [XFamilyRAndS] has received or given any financial help from their children.]

CA017[ ] In the past year, while [XChildPanAliveName[ ]] was not living with [XFamilyR] , [XFamilyRAndS]

How much financial support did [XChildPanAliveName[ ]]

receive? The total amount of money given is [hc([0, ÿ), , ÿ1), sc([0, 500000], , ÿ1), ub([ÿ1], [100, 500, 1500, 5000, 20000])] **(CA017_1[i])** yuan, of which the regular amount of money given is [hc([0, ÿ), , ÿ1), sc([0, 500000], , ÿ1), ub([ÿ1], [100, 200, 1000, 3000, 10000])] **(CA017_2[i])** yuan (such as regular living expenses, monthly water and electricity bills, etc.)

Machine Translated by Google

telephone bills, mortgage payments, rent or other regular expenses); Total in-kind support of [hc([0, ÿ), , ÿ1), sc([0, 500000], , ÿ1), ub([ÿ1], [100, 500, 1500, 5000, 20000])] **(CA017_3[i])** yuan, of which regular in-kind support of [hc([0, ÿ), , ÿ1), sc([0, 500000], , ÿ1), ub([ÿ1], [100, 200, 1000, 3000, 10000])] **(CA017_4[i])** yuan (e.g. regular provision of food, grocery shopping, clothing or other items). **[Interviewer's note:** This includes financial support received from [XChildPanAliveName[ ]]'s children. Regular means giving money or things on a monthly, quarterly or semi-annual basis, with a roughly fixed

time. The amount given regularly should not exceed the total amount given. If no money or things were given, please fill in "0"; if the respondent refuses to answer or forgets, please fill in "-1"]

CA018[ ] In the past year, when [XChildPanAliveName[ ]] was not living with [XFamilyR] , [XFamilyRAndS]

How much financial support does [XChildPanAliveName[ ]] give? The total amount of money given is [hc([0, ÿ), , ÿ1), sc([0, 500000], , ÿ1), ub([ÿ1], [100, 500, 1500, 5000, 20000])] **(CA018_1[i])** yuan, of which the regular amount of money given is [hc([0, ÿ), , ÿ1), sc([0, 500000], , ÿ1), ub([ÿ1], [100, 200, 1000, 3000, 10000])] **(CA018_2[i])** yuan (such as regular living expenses, payment of monthly water, electricity and telephone bills, payment of mortgage, rent or other regular expenses). The total amount of goods given is [hc([0, ÿ), , ÿ1), sc([0, 500000], , ÿ1), ub([ÿ1], [100, 500, 1500, 5000, 20000])] **(CA018_3[i])** yuan, of which regular in-kind gifts total [hc([0, ÿ), , ÿ1), sc([0, 500000], , ÿ1), ub([ÿ1], [100, 200, 1000, 3000, 10000])] **(CA018_4[i])** yuan (such as regular provision of snacks, grocery shopping, clothing or other items). **[Interviewer's note: This includes** financial support for the child of [XChildPanAliveName[ ]]. Regular means giving money or things on a monthly, quarterly or semi-annual basis, with a roughly fixed time frame; the amount given regularly should not exceed the total amount given. If no money or things were given, please fill in "0";

if the respondent refuses to answer or forgets, please fill in "-1"]

CA019[ ] Has the COVID-19 outbreak this year affected the relationship between [XFamilyRAndS] and [XChildPanAliveName[ ]]?

1. Better
2. Worse
3. No effect

CA020[ ] Did the epidemic after the Spring Festival affect the income of [XChildCoupleDis[ ]]? Increase, decrease, or no effect?

[Interviewer Note: If the respondent refuses to answer or forgets, please fill in "-1". If conditions permit, you can ask your children about the impact of the epidemic on their income.]

1. Increase, estimated increase by percent [hc((0, ÿ), , ÿ1), sc((0, 100], , ÿ1)] **(CA020_1[i])**
2. Decrease, estimated decrease by percent [hc((0, 100], , ÿ1)] **(CA020_2[i])**
3. No effect
997. Don't know
999. Refuse to answer

CB. Household member information

[Quote: We would like to know about the situation of other household members besides [XFamilyRAndS] . Household members refer to people who live with [XFamilyRAndS] and share the income and expenditure of life.]

Machine Translated by Google

CB001 Which of the following people are members of the household of [XFamilyRAndS] ? (Multiple choices are allowed)

[Interviewer's note: Household members refer to people who live with the interviewee and share the same income and expenditure. It is difficult to determine whether children are household members of the interviewee in the field. Generally, this can be determined by asking the interviewee whether he or she has separated from a certain child.] 1-25. [XChildAliveName[ ]]

99. None of the above [conflict(99, [99])]

ÿQuote: [XIntroCB002] ÿ

CB002 [XHHOtherDis] Who are the household members of [XFamilyRAndS] ? Please check and fill in the name **(CB002_1[i])**

no other ________ 1-10. 99. There are household members [conflict(99, [99])]

CB003[ ] What is the gender of household member [XHHOtherMemberName[ ]]? 1.

Male 2.

Female

CB004[ ] How old is household member [XHHOtherMemberName[ ]] this year? ÿ1)] ________ [hc([0, 120], , ÿ1), sc([0, 100], ,

years old

[Interviewer's note: If the respondent refuses to answer or forgets, please fill in "-1"]

CB005[ ] [XHHOtherMemberName[ ]] Who is [XFamilyR] ? 1. Daughter-in-law or son-in-law

2. Grandson or granddaughter 3.

Brother-in-law or brother-in-law 4.

Father 5.

Mother 6.

Mother-in-law 7.

Father-in-law 8.

Children 9.

Brothers and sisters

10. Other relatives, please specify: **(CB005_1[i])**

CB006[ ] [XHHOtherMemberName[ ]] is the spouse of which child of [XFamilyR] ?

[Interviewer Note: Load the list of surviving children here. If the household member is the spouse of a deceased child, please add the name of the deceased child in the text box] 1-25.

[XChildAliveName[ ]] 99. Other, name: **(CB006_1[i])**

CB007[ ] [XHHOtherMemberName[ ]] is the child of which child of [XFamilyR] ?

[Interviewer Note: Load the list of surviving children here. If the household member is the child of a deceased child, please add the name of the deceased child in the text box] 1-25.

[XChildAliveName[ ]] 99. Other, name: **(CB007_1[i])**

CC. Family contact during the Spring Festival

Machine Translated by Google

[Quote: The following questions are about the impact of the epidemic on the [XFamilyR] family reunion during the Spring Festival.]

CC001During this year's Spring Festival, were [XFamilyR] 's children or grandchildren unable to return home for the New Year due to the epidemic?

1. Yes, there are [hc([1, 15], , ÿ), sc([1, 8], year , ÿ)] **(CC001_1)** children and grandchildren cannot come home
2. No

CC002During this year's Spring Festival, are there any relatives or friends who are unable to visit [XFamilyR] due to the epidemic ?

1. Yes
2. No

CC003 Compared with previous Spring Festivals, have younger generations and relatives and friends given [XFamilyRAndS] less money?

1. Yes, about [hc((0, 100000], , ÿ), sc((0, 50000], , ÿ)] **(CC003_1)** yuan
2. No

CC004During the Spring Festival, elders usually give red envelopes to younger generations. So did [XFamilyRAndS] save a lot of money by giving out red envelopes this Spring Festival?

some money?

1. Yes, about [hc((0, 100000], , ÿ), sc((0, 50000], , ÿ)] **(CC004_1)** yuan
2. No

CC005During the first month of this year, did [XFamilyRAndS] live together most of the time?

1. Yes
2. No

CC006[ ] [XCouLiveList[ ]] Who did you live with during the first month of this year? (Multiple choices are allowed)

[Interviewer's note: including short-term residence]

1-25. [XChildPanAliveName[ ]]
26-35. [XHHOtherMemPreload[ ]]
99. None of the above
[conflict(99, [99])]

CC007[ ] In addition to the people just mentioned, who else lived with [XCouLiveList[ ]] during the first month of this year? (Multiple selections are allowed)

[Interviewer's note: including short-term residence]

1. ______ **(CC007_1[i])**
2. ______ **(CC007_2[i])**
3. ______ **(CC007_3[i])**
4. ______ **(CC007_4[i])**
5. ______ **(CC007_5[i])**
6. ______ **(CC007_6[i])**
7. ______ **(CC007_7[i])**
8. ______ **(CC007_8[i])**
9. ______ **(CC007_9[i])**
10. ______ **(CC007_10[i])**
99. No one else
[conflict(99, [99])]

CC008[ ] Compared with previous years, has the number of people living with [XCouLiveList[ ]] increased or decreased during the first month of this year?

1. Added

Machine Translated by Google

2. Decreased 3.

No change

Auxiliary variable definition

XFamilyR family respondent nameif (!

```
empty("XMainRS")) { if
    (equal("CA001", "1"))
        { add("XFamilyR", value("XMainR"))
        add("XFamilyS", value("XMainRS"))
    } else if (equal("CA001", "2")) {
        add("XFamilyR", value("XMainRS"))
          add("XFamilyS", value("XMainR"))

} } else
    { add("XFamilyR", value("XMainR"))
    add("XFamilyS", "")
}
```

XFamilyS Family Respondent's Spouse Name

see above

The **XFamilyRAndS** question stem loads the family respondent and spouse's name together. If a spouse exists, it will be displayed as "[family respondent's name]

and [spouse name]", otherwise only the household respondent name is displayed.

```
if (!empty("XFamilyS"))
    { add("XFamilyRANdS",
    value("XFamilyR")+"and"+value("XFamilyS")) }
else
    { add("XFamilyRANdS", value("XFamilyR") )
}
```

XChildNumThe number of children whose names are not empty

```
add("XChildNum", "0")
for (var i = 1; i <= value("ZChildNum"); i++) { if (!
    empty("ZChildName[i]"))
        { add("XChildNum", value("XChildNum")+1)
    }
}
```

XChildGenderDis Displays the loaded gender in the title

```
if (equal("ZChildGender[i]", "1"))
    { add("XChildGenderDis[i]", "Male") }
else if (equal("ZChildGender[i]", "2")) { add
    ("XChildGenderDis[i]", "female") } else

{ add("XChildGenderDis[i]", "missing")
}
```

XChildAlive Whether the child is alive when accessed

```
if (equal("CA002[i]", "1"))
    { add("XChildAlive[i]", "1") }
else if (equal("CA002[i]", "2")) { add
    ("XChildAlive[i]", "0")
}
```

XChildAliveName: The name of the child who is alive at the time of access. If the child has passed away, it is empty.

```
if (equal("XChildAlive[i]", "1"))
    { add("XChildAliveName[i]", value("ZChildName[i]"))
}
```

XChildAliveNum Number of living children

Machine Translated by Google


```
add("XChildAliveNum", "0") for
(var i = 1; i <= value("ZChildNum"); i++) { if (!
        empty("ZChildName[i]") && equal("XChildAlive[i] ", "1"))
                { add("XChildAliveNum", value("XChildAliveNum")+1)
        }
}
```

XChildPanAliveNameName of the children alive at the beginning of the pandemic, blank for those who have passed away

```
if (equal("XChildPanAlive[i]", "1"))
        { add("XChildPanAliveName[i]", value("ZChildName[i]"))
        add("XChildPanAliveNum", value("XChildPanAliveNum")+1 )
}
```

XChildPanAliveNumNum number of children alive at the beginning of the epidemic

see above

XChildPanAlive Whether the child was alive when the epidemic started

```
if (equal("XChildAlive[i]", "1"))
        { add("XChildPanAlive[i]", "1") }
else if (greater("2019", "CA003_1[i]", true)) || (equal("CA003_1[i]", "2020") && equal("CA003_2[i]", ÿ "1"))

 { add("XChildPanAlive[i]", "0") } else if
( equal("CA003_1[i]", "2020") && greater("CA003_2[i]", "1", false)) { add("XChildPanAlive[i]",
        "1")
}
```

XChildGenderChild Gender

```
if (!empty("ZChildGender[i]"))
        { add("XChildGender[i]", value("ZChildGender[i]")) } else

{ add("XChildGender[i]", value("CA006[ i]"))
}
```

XChildBirthChildBirth year of birth

```
if (!empty("ZChildBirth[i]"))
        { add("XChildBirth[i]", value("ZChildBirth[i]")) } else

{ add("XChildBirth[i]", value("CA005[ i]"))
}
```

XChildEdu Highest educational level of children

```
if (!empty("ZChildEdu[i]"))
        { add("XChildEdu[i]", value("ZChildEdu[i]")) } else

{ add("XChildEdu[i]", value("CA007[ i]"))
}
```

XChildCoupleDis If a spouse exists, then display "[child's name] and his/her spouse"

```
if (equal("CA010[i]", "1") || equal("CA010[i]", "2"))
        { add("XChildCoupleDis[i]", value("XChildPanAliveName[i]")+"and his/her spouse")
} else if (equal("CA010[i]", "3") || equal("CA010[i]", "4") || equal("CA010[i]", "5") || equal ("CA010[i]", ÿ "6") || equal("CA010[i]",
 "997") || equal("CA010[i]", "999")) { add("XChildCoupleDis[ i]",
        value("XChildPanAliveName[i]"))
}
```

XIntroCB002 If the number of living children is 0 when accessed, then display the quote in CB002

```
if (greater("XChildAliveNum", "0"))
        { add("XIntroCB002", "") } else

{ add("XIntroCB002", "We want to know about the situation of other household members besides "+pre("XFamilyRAndS")+",
    ÿHousehold members refer to those who live with "+pre("XFamilyRAndS")+" and share the same income and expenditure")
}
```

Machine Translated by Google

If **XHHOtherDis** needs to ask whether the child is a household member, it will display "In addition to the household member just selected, there are also"

```
if (equal("XChildAliveNum", "0"))
    { add("XHHOtherDis", "")
} else if (greater("XChildAliveNum", "0"))
    { add("XHHOtherDis", "In addition to the household members just selected, there are also")
}
```

XHHMemberNum is the number of household members, the minimum is 0 and the maximum is 25

```
for (var i1 = 1; i1 <=25 ; i1++) { if
    (selected("CB001", i1))
        { add("XHHMemberNum", value("XHHMemberNum")
        +1) add("XHHMemberName["+value ("XHHMemberNum")+"]", value("XChildAliveName["+i1+"]"))
        add("XHHMemberAge["+value("XHHMemberNum")+"]", 2020-value("XChildBirth["+ i1+"]"))
    }

} for (var i1 = 1; i1 <=10 ; i1++) { if
    (selected("CB002", i1))
            { add("XHHMemberNum", value("XHHMemberNum")
            +1) add("XHHMemberName["+ value("XHHMemberNum")+"]", value("CB002_1[i1]"))
    }
}
```

XHHMemberNameHousehold member name

see above

XHHMemberAgeHousehold member age

```
for (var i1 = 1; i1 <=25 ; i1++) { if
    (selected("CB001", i1)) {
        add("XHHMemberAge["+value("XHHMemberNum")+"]", 2020-value("XChildBirth["+i1+"]"))
    }

} if (!equal("CB004[i]", "-1"))
    { add("XHHOtherAgeIter", value("XHHOtherAgeIter")+1)
    add("XHHMemberAge["+value("XHHOtherAgeIter")+" ]", value("CB004[i]")) } else

{ add("XHHOtherAgeIter", value("XHHOtherAgeIter")+1)
    add("XHHMemberAge["+value("XHHOtherAgeIter")+"]", " ")
}
```

XHHOtherMemberNumNum number of other household members, maximum is 10

```
add("XHHOtherMemberNum", "0")
for (var i1 = 1; i1 <=10 ; i1++) { if
    (selected("CB002", i1))
            { add("XHHOtherMemberNum", value("XHHOtherMemberNum")
            + 1) add("XHHOtherMemberName["+value("XHHOtherMemberNum")+"]", value("CB002_1[i1]"))
    }
}
```

XHHOtherMemberNameOther household member's name

see above

XHHOtherAgeIter calculates the intermediate variable of household member age

```
if (equal("XChildAliveNum", "0"))
    { add("XHHOtherAgeIter", "0") }
else if (selected("CB001", 99))
    { add("XHHOtherAgeIter", "0") }
else
    { add("XHHOtherAgeIter", count("CB001"))
}
```

XAffectReunion: Is the Spring Festival family reunion affected by the epidemic?

```
if (equal("CC001", "1") || equal("CC002", "1"))
    { add("XAffectReunion", "1") }
else {
```

Machine Translated by Google


```
        add("XAffectReunion", "0")
    }
```

XCouLiveList generates a list of names to be asked together or separately, depending on whether the household respondent lives with their spouse.

```
    if (empty("XFamilyS"))
        { add("XCouLiveList[1]", value("XFamilyR")) }
    else if (!empty("XFamilyS") && equal("CC005", "2"))
        { add("XCouLiveList[1]", value("XFamilyR"))
        add("XCouLiveList[2]", value("XFamilyS")) }
    else
        { add("XCouLiveList[1]", value("XFamilyRAndS") )
    }
```

XHHOtherMemPreload generates a list of other household members starting from i=26

```
    for (var k = 1; k <= 10; k++) {
        add("XHHOtherMemPreload["+(k+25)+"]", value("XHHOtherMemberName[k]"))
    }
```

ZChildNameChild Name

Machine Translated by Google

This page intentionally left blank

Machine Translated by Google

D Health status and function

Machine Translated by Google

D. Proxy mode confirmation

proxy_5 Interviewer record: For the health module, do you use the proxy questionnaire mode?

1. Yes 2.

No

DA. Health status (I)

[Quote: Now I will ask some questions about [XRName] 's health.]

DA001 How do you think your health is? Very good, good, fair, poor, or very poor?

[Interviewer's note: All options must be read

out] 1. Very

good 2.

Good 3.

Average 4.

Not good 5.

Very bad 997. Don't know

DA002[ ] Compared to [ZIWTime] , [XRName] 's [XChroDisType[ ]] is better. Is it about the same as before or worse?

1. Better 2.

Worse 3.

About the same as before

99. Did not have this disease during the last visit

DA002_1[ ] At [ZIWTime] , [XRName] knew he had [XChroDisType[ ]]. Compared to [ZIWTime] , is [XRName] 's [XChroDisType[ ]] better, about the same as

before, or worse?

1. Better 2.

Worse 3.

About the same as before

99. Did not have this disease during the last visit

DA003[ ] Has a doctor ever told [XRName] that he has [XChroDisType[ ]]?

1. Yes 2.

No

DA004[ ] Does [XRName] know that he has [XChroDisType[ ]]?

1. Know that you have it 2.

Know that you don't have

it 3. Don't know if you have it

DA005 In the past month, has [XRName] visited a medical institution for outpatient treatment or received home medical services? (excluding visits to

Hospital for physical

examination)

1. Yes 2. No

Machine Translated by Google

DA006 In the past month, how many times did [XRName] visit a medical institution for outpatient treatment (including home medical services)? [hc((0, 99) , ÿ), sc([1, 20),

, , ÿ)] times

DA007Has [XRName] been hospitalized in the past year ?

[Interviewer's note: The past year refers to the year before today]

1. Yes 2.

No

DA008 In the past year, how many times has [XRName] been hospitalized? [hc((0, 99), ________ , ÿ), sc([1, 20), , ÿ)] times

[Interviewer note: Hospitalizations that did not occur in the past year are not counted. The past year refers to the year before today.]

DA009During the epidemic, did [XRName] ever need to see a doctor, including a dentist, but was forced to postpone or was unable to do so due to the epidemic?

Can I go and

see it? 1.

Yes 2. No

DA010 Could you please specify what kind of illness [XRName] wanted to see or what kind of medical service he wanted to get during the epidemic, and whether it was

postponed or cancelled? (Multiple choices

are allowed) [Interviewer's note: Please read out each option and check all that apply]

1. Major surgery that requires hospitalization

2. Minor surgery that can be done in an outpatient clinic or day ward 3.

Going to a doctor's office 4.

Going to get prescription

drugs 5. Going to the dentist, oral

treatment 6. Others, please briefly explain **(DA010_1)**

DA011 [XRName] wanted to see an outpatient during the epidemic. Was it because of new symptoms or diseases, treatment of existing diseases, or routine physical

examination and screening? (Multiple choices)

1. New symptoms or diseases 2. Treatment

of existing diseases 3. Routine physical

examination screening

DA012 Why was [XRName] 's visit to the doctor delayed or why was he not able to go? (Multiple choices are allowed, optional) 1. Unable

to make an appointment, or the hospital's regular appointments were

cancelled 2. The hospital rescheduled all regular treatment

arrangements 3. I decided to wait 4. I am afraid of going

to the hospital 5. Others, please

specify **(DA012_1)** ________

DA013 Since [ZIWTime] , when was [XRName]' s last routine physical examination? (Note: This does not include CHARLS physical examinations.

[Interviewer **Note: Use** 4 digits to

represent the year and fill in the month according to the actual month. For example, January is written as "1" instead of "01"

and December is written as "12". If

1. ________ you can't remember the month, please fill in "-1"] [hc([1900, 2020], , ÿ), sc([1920, 2020], , ÿ)] **(DA013_1)**ÿ[hc([1, 12],

, ÿ1)] **(DA013_2)** month

2. Have not had a routine physical examination since the last visit

DA014 When was [XRName] 's last routine physical examination (note: this does not include examinations done at medical institutions)?

Machine Translated by Google

ÿ**Interviewer's note: Use** 4 digits to represent the year and fill in the month according to the actual month. For example: January is written as "1" instead of "01", and December is written as

1. ________ "12". If you cannot remember the month, please fill in "-1"ÿ [hc([1900, 2020], , ÿ), sc([1920, 2020], , ÿ)] **(DA014_1)**ÿ[hc([1, 12], , ÿ1)] **(DA014_2)** 2 . Never had a regular physical examination in my life

DA. Health status (II)

DA019Since [ZIWTime] , has [XRName] been involved in a traffic accident or any major accidental injury and received medical treatment?

1. Yes
2. No

DA020 Has [XRName] ever been involved in a traffic accident or any major accidental injury and received medical treatment?

1. Yes
2. No

DA021 Do the injuries caused by the accident affect [XRName]' s current daily

activities? 1. Yes 2. No

DA022Since [ZIWTime] , has [XRName] fallen ? 1. Yes 2. No

DA023 Has [XRName] ever fallen? 1. Yes 2.

No

DA024How many times did you fall and get injured seriously enough to need medical treatment? [hc([0, 99], , ÿ), sc([0, 20), , ÿ)] times

DA025 Has [XRName] had any hip fractures since [ZIWTime] ?

[Interviewer's note: The "hip bone" refers to the bones in the human waist, which are divided into two parts, left and right. In childhood, the hip bone is divided into the ilium, ischium and pubis, and connected by cartilage. In adulthood, the cartilage between them will ossify and become a whole, namely the hip bone.]

1. Yes
2. No

DA026 Has [XRName] ever had a broken hip?

[Interviewer's note: The "hip bone" refers to the bones in the human waist, which are divided into two parts, left and right. In childhood, the hip bone is divided into the ilium, ischium and pubis, and connected by cartilage. In adulthood, the cartilage between them will ossify and become a whole, namely the hip bone.]

1. Yes
2. No

DA027 Does [XRName] often suffer from pain? Not at all, a little, some, quite a lot, or a lot?

[Interviewer's note: This question is about pain in all parts of the body] 1. Not at all 2. A little

3. Some 4. Quite a lot

Machine Translated by Google

5. A lot

DA028Which parts of the body do you feel pain in? Please list all parts.

1. Head

2. Shoulders

3. Arms 4.

Wrists 5.

Fingers 6.

Chest

7.

Stomach

8. Back

9. Waist 10.

Buttocks

11. Legs 12.

Knees 13.

Ankles 14.

Toes 15.

Neck 16. Other parts, please specify **(DA028_1)**

DA029 Next, I would like to ask you a question about your subjective life expectancy, which reflects your expectations of your own health status. Assuming there are five levels, the lowest level represents the least possibility and the highest level represents the most likely, how likely do you think it is that you will live to the age of [XFAge-Possibility] ? Is it almost impossible, unlikely, possible, very likely, or

absolutely certain? 1.

Almost impossible

2. Unlikely 3.

Possible 4.

Very likely 5.

Absolutely certain 997. Don't know

DA. Health status (III)

DA030 In the past month, how many hours did [XRName] actually sleep every night on average? (May be shorter than the time [XRName] spent lying in bed) , -1)] hours [Interviewer's note [hc([0, 24], : If you don't know, please fill in -1]

DA031 In the past month, how long did [XRName] usually take naps? [hc([0, 300], , ÿ1)] minutes

[Interviewer's note: If the respondent does not take a nap, please record it as 0; if you do not know, please fill in -1]

[Quote: Below are some questions about how much time [XRName] spends doing these activities on a typical week?]

DA032[ ] Please recall the [XPsyActType[ ]] that [XRName] usually does every week . Just recall the [XRName] every time he exercises. At least ten minutes of activity. Does [XRName] typically do this type of activity for at least ten minutes per week?

1. Yes

2. No

Machine Translated by Google

DA033[ ] [XRName] How many days per week do you usually do [XPsyActType[ ]] for at least ten minutes? ______ [hc([1, 7], , ÿ)] day

DA034[ ] During the days that you were doing [XPsyActType[ ]] , how much time did [XRName] spend doing [XPsyActType[ ]]?

1. <2 hours 2.

>=2 hours

DA035[ ] During the days that [XPsyActType[ ]] was performed, how much time per day did [XRName] spend performing [XPsyActType[ ]]?

1. <30 minutes 2.

>=30 minutes

DA036[ ] During the days that [XPsyActType[ ]] was performed, how much time per day did [XRName] spend performing [XPsyActType[ ]]?

1. <4 hours 2.

>=4 hours

DA037[ ] Do [XPsyActType[ ]] because of work needs, entertainment activities, physical exercise, or other reasons? 1. Work

needs 2.

Entertainment

3. Physical exercise

4. Others **(DA037_1[i])**

DA038 [XRName], have you participated in the following social activities in the past month? (Multiple choices are allowed)

1. Visiting relatives, socializing

with friends 2. Playing mahjong, chess, cards, and going to

community activity rooms 3. Providing help to relatives, friends, or neighbors who do

not live with you 4. Dancing, fitness,

practicing qigong, etc. 5.

Participating in community activities 6. Volunteer activities, or charity activities, or taking care of patients or disabled

people who do not live with you 7. Going

to school or participating in training courses 8. Other social activities,

please specify **(DA038_1)** 9. None of the above

[conflict(9, [9])]

DA039[ ] In the past month, how often did [XRName] do the activities [XSocType[ ]] I just mentioned ? About every day,

Almost every week or not often?

1. Almost every day 2.

Almost every week 3.

Not often

DA040Did you go online in the past month? Including chatting on your mobile phone, reading news, watching videos, playing games, managing finances, etc.

1. Yes

2. No

DA041 Which of the following tools does [XRName] use to access the Internet? (Multiple

choices are allowed)

1. Desktop computer 2.

Laptop computer 3. Tablet computer

(such as

IPAD) 4. Mobile phone 5. Other devices, please specify **(DA041_1)**

Machine Translated by Google

DA042 What does [XRName] usually do when he/ she is online? (Multiple

choices are

allowed) 1. Chat

2. Read news

3. Watch videos

4. Play

games 5. Manage finances 6. Others, please specify **(DA042_1)**

DA043 Will [XRName] use mobile payment, such as Alipay, WeChat Wallet, etc.? 1. Yes 2. No

DA044 Does [XRName] use WeChat? 1. Yes 2. No

DA045 [XRName] Should I post on WeChat Moments? 1.

Post 2.

Don't post

DA046 Has [XRName] ever smoked? (Cigarettes, dry tobacco, pipe smoking or chewing tobacco)

1. Yes

2. No

DA047 [XRName] is still smoking or has he quit smoking? 1. Still

smoking 2. Quit

smoking 3.

Never smoked

DA048 [XRName] When smoking, what do you usually smoke?

1. Smoking with a pipe (pouch, dry tobacco)

2. Rolling your own

cigarettes 3. Cigarettes

with filters 4. Cigarettes

without

filters 5. Cigars 6. Hookahs

DA049 [XRName] At what age or year did you successfully quit smoking this time?

[Interviewer Note: If you don't know,

please fill in -1] 1. Age [hc([0, 120], , ÿ1), sc((0, 100], , ÿ1)] **(DA049_1)** years old 2. Year [hc([1900, 2020], ,

ÿ1), sc([1920, 2020], , ÿ1)] **(DA049_2)** years old

DA050_1 How many cigarettes does [XRName] smoke on average per day? [hc([0, 200], , ÿ)]

DA050_2 How many cigarettes did [XRName] smoke on average per day before quitting smoking? [hc([0, 200], , ÿ1)]

ÿInterviewer's note: If you don't know, please fill in -1ÿ

DA051Did [XRName] drink alcohol in the past year , including beer, wine, rice wine, yellow wine or white wine, medicinal wine, etc.?

What is the rate?

Machine Translated by Google

1. Drinking more than once a month 2.

Drinking less than once a month 3. Not

drinking at all

DA052 In the past year, how many times did [XRName] drink alcohol on average

per month? 1. Once

a month 2. 2-3 times a

month 3. Once a

week 4. 2-3 times a

week 5. 4-6 times a

week 6. Once a day

7. Twice a day 8.

More than twice a day

DB. Physical impairment and helpers (I)

[Quote: Now we would like to know about [XRName]'s daily life. Is [XRName] currently experiencing any physical, mental, emotional or memory problems?

The reasons below make it difficult to complete some of the daily activities we mentioned below. The "difficulties" we refer to do not include those that can be resolved within three months.

Disaster.ÿ

DB001 May I ask if [XRName] has difficulty dressing himself due to health or memory problems? Dressing involves taking clothes out of the closet.

Clothes, put on clothes, button up, and tie a belt.

1. No difficulty 2.

Difficult but can still be completed 3.

Difficult and need help 4. Unable to

complete

DB002Did anyone help [XRName] when he was getting dressed ? 1. Yes

2. No

DB003 Does [XRName] have difficulty bathing due to health or memory issues? 1. No difficulty 2. Difficulty

but still able to do it

3. Difficulty and need help 4. Unable to

do it

DB004 Was there anyone who helped [XRName] when he was taking a

shower ?

1. Yes 2. No

DB005 Does [XRName] have difficulty eating by himself, such as picking up food by himself, due to health and memory problems? (Definition: Eating by oneself

is defined as eating after the food is prepared) 1. No difficulty

2. Difficulty but can

still complete 3. Difficulty, need help 4.

Unable to complete

Machine Translated by Google

DB006 Was there anyone who helped [XRName] when he was eating ? 1.

Yes 2. No

DB007 Does [XRName] have any difficulty getting up or out of bed?

1. No difficulty 2.

Difficulty but still able to do it 3. Difficulty,

need help 4. Unable to do it

DB008 Is there anyone to help [XRName] get up or get out of bed ? 1.

Yes 2. No

DB009 Does [XRName] have difficulty using the toilet, including squatting and standing, due to health and memory reasons? 1. No difficulty 2.

Difficulty but can still

complete 3. Difficulty, need help 4.

Unable to complete

DB010 Is there anyone who helps [XRName] when he goes to

the

toilet ? 1. Yes 2. No

DB011 Does [XRName] have difficulty controlling his bowels and bladder due to health or memory reasons? (Can he use a catheter or

Urine bag is considered to be able

to control self-care)

1. No difficulty 2. Difficulty but can still

complete 3. Difficulty, need help 4.

Unable to complete

DB012 Does [XRName] have difficulty doing housework due to health or memory problems? (Definition: Doing housework, we

Refers to house cleaning, washing dishes, arranging bedding and room furnishings)

[Interviewer Note: If the respondent cannot mop the floor but can scrub the table, or the respondent cannot arrange heavy bedding but can arrange some

light bedding, please select

(3)] 1. No difficulty 2.

Difficulty but can still complete 3.

Difficulty, need help 4. Unable to

complete

DB013 Does anyone help [XRName] with housework ? 1. Yes 2. No

DB014 Does [XRName] have difficulty cooking due to health and memory problems? (Definition: We define cooking as preparing raw materials, cooking dishes, and

serving them on the table) [Interviewer's

note: If the respondent needs help with washing and cutting vegetables due to health reasons, or the respondent can only cook rice but not dishes, that is,

the respondent can only complete some simple cooking actions due to health reasons, then select (3)]

Machine Translated by Google

1. No difficulty 2.

Difficult but can still be completed 3.

Difficult and need help 4. Unable

to complete

DB015 Did anyone help [XRName] when he was cooking ? 1. Yes 2.

No

DB016 Does [XRName] have difficulty going to the store to buy groceries due to health and memory issues?

Buying something means deciding what to buy

and paying for it. 1.

No difficulty 2. Difficult but still can be

done 3. Difficult and need help 4.

Unable to complete

DB017 Does anyone help [XRName] go to the store to buy groceries, etc.? 1. Yes

2. No

DB018 Does [XRName] have difficulty making phone calls due to health or memory issues? 1. No difficulty

2. Difficulty but can

still make the call 3. Difficulty, need

help 4. Unable to make the call

DB019 Was there anyone to help [XRName] when I called ? 1. Yes 2.

No

DB020Is [XRName] having trouble taking his own medicine due to health and memory issues? Does taking medicine mean being able to remember when to take it ?

1. No difficulty

2. Difficulty but can

still finish 3. Difficulty, need help 4.

Unable to finish

DB021 Did anyone help [XRName] when he took the medicine ? 1.

Yes 2.

No

DB022Does [XRName] have trouble managing money, such as paying bills, keeping track of spending, or managing

Financial

management? 1.

No difficulty 2. Difficult but can be

completed 3. Difficult, need help

4. Unable to complete

DB023 Is there anyone helping [XRName] manage his money?

Machine Translated by Google

1. Yes

2. No

DB. Physical impairment and helpers (II)

DB024 Please tell me how to deal with the above (dressing, bathing, eating, getting up, going to the toilet, housework, cooking, shopping, making phone calls, taking medicine, managing money, etc.)

Who helped [XRName] in his or her difficulties ? (Multiple

choices are

allowed) 1. Spouse 2. Parents, parents-in-law,

father-in-law, mother-in-law 3. Children, daughters-in-law/

sons-in-law, grandchildren 4. Brothers and sisters and their spouses and children, and brothers and sisters of

[XRName]'s

spouse and their spouses and children 5. Other relatives 6. Hired personnel (such as nanny), a total of [hc((0, 99), , ÿ1), sc([1,

10), , ÿ1)] **(DB024_1)** 7. Volunteers or

voluntary agency staff

8. Nursing home staff 9. Home-based

elderly care service agency

staff 10. Help provided by the community 11. Other personnel, please specify **(DB024_2)**

DB025 Among parents, parents-in-law, father-in-law, and mother-in-law, which ones help [XRName] ? (Multiple choices are allowed)

1. Father 2.

Mother 3.

Father-in-law 4.

Mother-in-law

DB026 Help [XRName] 's children, daughters-in-law/sons-in-law, grandchildren/grandchildren. Which of the following children's families are they from? (Multiple choices are allowed)

1-25. [XChildPanAliveName[ ]] 26-35. Other

children, named **(DB026_1[i])** ________

DB027[ ] Who in [XHelperChild[ ]]'s family personally helped [XRName] ? (Multiple selections allowed) 1.

[XHelperChild[ ]] himself

2. Spouse of [XHelperChild[ ]] 3. Children

of [XHelperChild[ ]], i.e. grandchildren of [XRName], how many children of [XHelper-Child[ ]] personally help [XRName] [hc((0, 99), ,

ÿ1), sc([1, 10), , ÿ1)] **(DB027_1[i])** ________

DB028 Help [XRName] 's brothers and sisters and their spouses and children. [XRName]' s spouse's brothers and sisters and their spouses and children. Which of

the following brothers and sisters' families are they

from? (Multiple choices are

allowed) 1-30. [XSibName[ ]] 31-40. Other brothers and sisters, whose names are **(DB028_1[i])**

DB029[ ] Who in [XHelperSib[ ]]'s family personally helped [XRName] ? (Multiple selections allowed)

1. [XHelperSib[ ]] himself

2. Spouse of [XHelperSib[ ]] 3. Children

of [XHelperSib[ ]], i.e. nephews and nieces of [XRName] , who personally help [XRName] , ÿ1)] **(DB029_1[i**

[XHelperSib[ ]] has several children [hc((0, 99),]) ________ , ÿ1), sc([1, 10),

Machine Translated by Google

DB030 How many other relatives have personally provided assistance to [XRName] ? [hc((0, 99), ________ , ÿ1), sc([1, 10), , ÿ1)]

[Interviewer note: If you don't know, please fill in -1]

DB030_1Who are these people from [XRName] ? ________

DB031How many other people have personally helped [XRName] ? [hc((0, 99), ________ , ÿ1), sc([1, 10), , ÿ1)]

[Interviewer note: If you don't know, please fill in -1]

DB031_1Who are these people from [XRName] ? ________

DB032Among all the helpers listed below, please select the 7 types of people who helped [XRName] the most.

1-99. [XHelper[ ]]

DB033[ ] In the past month, how many days did [XHelpList[ ]] help [XRName] ? ________ [hc([0, 31], , ÿ1)] day

ÿInterviewer's note: If you don't know, please fill in -1ÿ

DB034[ ] On the days when [XHelpList[ ]] helped [XRName] , how many hours per day did he/she spend helping [XRName] ? [hc([0, 24], , ÿ1)]

________ hours [Interviewer's note: if it is less than one hour, please write 1; if you don't know, please write -1]

DB035[ ] [XHelpList[ ]] Do you live with [XRName] while caring for [XRName] ?

1. Yes
2. No

DB048Has the care [XRName] received changed because of the pandemic ? Has he received more care, or less?

1. More 2. Less 3. No change

DB. Physical impairment and helpers (III)

DB036 If [XRName] needs care in daily life, such as eating and dressing, will there be relatives or friends who can take care of him/her for a long time?

[XRName] ? 1. Yes 2. No

DB037 Who is he/she to [XRName] ? (Multiple choices are allowed) 1. Spouse 2. Parents, parents-in-law, father-in-law, mother-in-law 3. Children, daughter-in-law/son-in-law, grandchildren 4. Brothers and sisters and their spouses and children, brothers and sisters of [XRName]'s spouse and their spouses and children 5. Other relatives 6. Hired personnel (such as nanny), [hc((0, ________ , ÿ1), sc([1, 10), , ÿ1)] **(DB037_1)** bit 99), 7. Volunteers or personnel of voluntary organizations

Machine Translated by Google

8. Nursing home staff 9.

Home-based elderly care service agency

staff 10. Community assistance 11.

Other staff, please specify **(DB037_2)** ________

DB038 Among parents, parents-in-law, father-in-law, and mother-in-law, which ones will help [XRName] in the future? (Multiple choices are allowed)

1. Father 2.

Mother 3.

Father-in-law 4. Mother-

in-law

DB039 In the future , which of the following children, daughters-in-law/sons-in-law, grandchildren/grandchildren will help [XRName] ? (You can choose multiple children)

select)

1-25. [XChildPanAliveName[ ]] 26-35. Other children,

named **(DB039_1[i])** ________

DB040 will help [XRName] 's brothers, sisters, spouses, and children, [XRName] 's spouse's brothers, sisters, spouses, and children in the future.

Female, which of the following brothers and sisters' family do you belong to? (Multiple choice question)

1-30. [XSibName[ ]] 31-40. Other

siblings, named **(DB040_1[i])** ________

DB041 How many other relatives will personally help [XRName] in the future? [hc((0, 99), ________ , ÿ1), sc([1, 10)

, , ÿ1)]

[Interviewer note: If you don't know, please fill in -1]

DB042How many other people will personally help [XRName] in the future? [hc((0, 99), , ÿ1), sc([1, 10), ________

, ÿ1)]

[Interviewer note: If you don't know, please fill in -1]

[Quote: Now we would like to know if [XRName] has any physical problems that affect [XRName] 's ability to work.]

DB043 Do you think this statement is true for [XRName] : I cannot work or labor normally because of disability or health reasons. 1. I cannot work or labor normally at

all 2. I cannot work or labor for long periods of time

3. I have no problem doing it

DB044 Do you think this statement is true for [XRName] : I cannot do housework normally because of disability or health reasons. 1. I cannot do housework

normally at all 2. I cannot do housework for

a long time 3. I have no problem doing it

DB045 Interviewer observation: Did [XRName] seek help when filling out this part of the questionnaire?

[Interviewer Note: If you are assisting in answering, please record the respondent's response]

1. Never 2.

Sometimes 3. Most of

the time 4. The respondent

is not present and someone else fills it out for him/her

DB046 Interviewer observation: What is the relationship between the proxy filler and [XRName] ?

Machine Translated by Google

[Interviewer's note: What is the relationship between the person filling out the questionnaire on behalf of the interviewee? If you are not sure, please ask the person filling out the questionnaire on behalf of the interviewee.]

1. Spouse

2. Mother

3. Father

4. Mother- in-law

5. Father-in-law

6. Brothers and

sisters 7. Brother-in-law 8.

Children 9.

Spouse of children

10. Grandchildren

11. Other relatives

12. Helpers or other non-relatives

DB047 Interviewer observation: What is the main reason why [XRName] was not present and someone else filled in the form for him?

[Interviewer's note: record the reason for answering on behalf of others]

1. The respondent has a serious physical

disability 2. The respondent has a serious

mental disability 3. The

respondent refuses to be interviewed 4. Others, please specify **(DB047_1)**

DC. Cognition and Depression (I)

ÿQuote: First I will ask you some questions to check your memory and attention. Some of the questions are easy and some are difficult. ÿ

DC001 What year is this year?

1. Correct

2. Incorrect

997. Don't know

999. Refuse to answer

DC002 What season is it now? 1.

True 2.

False 997.

Don't know 999.

Refuse to answer

DC003What day of the month is today?

[Interviewer's note: The lunar calendar date is also correct. Interviewees are not allowed to check their phones or calendars.]

1. True 2.

False 997.

Don't know 999.

Refuse to answer

DC004 What day is today? 1.

True 2.

False

Machine Translated by Google

997. Don't

know999. Refuse to answer

DC005 What month is it now? [Interviewer's

note: The lunar month is also correct. Interviewees are not allowed to check their phones or calendars.]

1. True 2.

False 997.

Don't know 999.

Refuse to answer

DC006 How do you think your memory is now? Excellent, very good, good, fair or poor?

1. Excellent

2. Very

good 3.

Good 4.

Average 5.

Not good 997. Don't know

ÿQuote: Now I need you to listen carefully to what I say and then do what I say. Can you listen carefully to me now? Are you ready? Let's begin. Please calculate 100 minus 7, then subtract 7 from the number you get, and keep calculating. Please tell me the answer after each subtraction of 7 until you get the number you want. Until I say "stop".

DC007_1 Record the answer:

ÿInterviewer's note: After reading the instructions, do not give any instructions to the interviewee during the calculation process, and do not remind the interviewee what to do.

Please remember not to give any additional prompts, just say "continue"; If the interviewee

does not give a positive response, after the interviewer reminds "continue" three times, if the interviewee still does not know what to do, he or she can choose the "don't know" option;

The test here allows respondents to use paper and pen

to complete it. 1. Record the answer [] **(DC007_1_1)**

997. Don't know

999. Refuse to answer

DC007_2 Record Answer: 1.

Record Answer [] **(DC007_2_1)** 997. Don't know

999. Refuse to

answer

DC007_3 Record the answer:

1. Record the answer [] **(DC007_3_1)** 997. Don't

know 999. Refuse

to answer

DC007_4 Record the answer:

1. Record the answer [] **(DC007_4_1)** 997. Don't

know 999. Refuse

to answer

DC007_5 Record the answer:

Machine Translated by Google

1. Record the answer [] **(DC007_5_1)** 997. Don't know

999. Refuse to

answer

DC008 Interviewer observation: Did the interviewee use paper, pen or other auxiliary tools when answering these arithmetic

questions? 1. Used auxiliary

tools 2. Did not use auxiliary tools

DC009 Here is a picture. Please draw it here according to the picture. transferPic("DC009")

Please click on the blank box below to take a photo of the figure drawn by the interviewee and save it in the interview system **(DC009_photo)** [Interviewer

Note: If the figure copied by the interviewee meets the requirements

A) Two pentagons with four sides

intersecting, B) All angles within the pentagon

are complete, then

it is considered

correct] 1.

Correct 2. Incorrect 3. Not evaluated (the respondent was unable to complete due to

physical reasons) 997.

Don't know 999. Refuse to answer

DC. Cognition and Depression (Part 2)

DC010_1 Next I will read you ten words. Please follow me and read these ten words out loud one by one. Then I will ask you to

You recall these ten words. Do you understand? transferPic("XWordlist","9 3 1 2 10 4 5 6 8 7") [Interviewer's note: Please check whether the

respondent understands how to do this test. Read the words to the respondent at a

slow and steady pace and ask the respondent to repeat after you finish. Keep the frequency to about one word every two seconds. Only if the

respondent has hearing problems, you can show the respondent the word list and read the words while showing it. You should read the words out loud and ask the

respondent to repeat the word before switching to the next group of words.]

1. Yes

2. No

DC010_2 Next I will read you ten words. Please follow me and read these ten words out loud one by one. Then I will ask you to

You recall these ten words. Do you understand? transferPic("XWordlist","9 3 1 2 10 4 5 6 8 7") [Interviewer's note: Please check whether the

respondent understands how to do this test. Read the words to the respondent at a

slow and steady pace and ask the respondent to repeat after you finish reading. Keep the frequency to about one word every two seconds. Only

if the respondent has hearing impairment, you can show the respondent the word list and read the words while showing it. Before switching to the next group of

words, you should read it out loud and ask the respondent to

repeat the

word.] 1. Yes 2. No

DC010_3 Next I will read you ten words. Please follow me and read these ten words out loud one by one. Then I will ask you to

You recall these ten words. Do you understand? transferPic("XWordlist","9 3 1 2 10 4 5 6 8 7") [Interviewer's note: Please check whether the

respondent understands how to do this test. Read the words to the respondent at a

slow and steady pace and ask the respondent to repeat after you finish. Keep the frequency to about one word every two seconds. Only if the

respondent has hearing problems, you can show the respondent the word list and read the words while showing it. You should read the words out loud and ask the

respondent to repeat the word before switching to the next group of words.]

Machine Translated by Google

1. Yes
2. No

DC011 Interviewer observation: Please record why the respondent cannot complete this test?

(Multiple choices are allowed) 1. Refuse or are unwilling to take this test 2. Unable to speak for life 3. Unable to speak after entering old age

4. Deaf or hard of hearing 5. Others, please specify **(DC011_1)**

DC012Now please tell me the words you can think of.

[Interviewer Note: Read the words to the respondent at a slow, steady pace, and ask the respondent to repeat after you finish. Keep the frequency to about one word every two seconds. Only if the respondent is hearing impaired, you can show the word list to the respondent and read the words while you are showing it. You should read the words out loud and ask the respondent to repeat them before moving on to the next group of words. Give the respondent as much time as he or she needs, up to two minutes.] 1. [XWordlist[9]]

2. [XWordlist[3]]
3. [XWordlist[1]]
4. [XWordlist[2]]
5. [XWordlist[10]]
6. [XWordlist[4]]
7. [XWordlist[5]]
8. [XWordlist[6]]
9. [XWordlist[8]]

10. [XWordlist[7]] 11. Did not recall any words 12. Refuse to recall 13. Respondent did not understand or was unable to perform this test

[conflict(11, 12, 13, [11, 12, 13])]

[Quote: Now I will read you the same series of words as before, but in a different order. Please follow me and read these ten words out loud one by one. Then I will ask you to recall these ten words. Are you ready?]

DC013 Now tell me the words you can think of. transferPic("XWordlist","5 2 9 10 3 1 8 4 6 7")

[Interviewer Note: Read the words to the respondent at a slow, steady pace, and ask the respondent to repeat after you finish. Keep the frequency to about one word every two seconds. Only if the respondent is hearing impaired, you can show the word list to the respondent and read the words while you show it. You should read the words out loud and ask the respondent to repeat them before moving on to the next group of words. Give the respondent as much time as he or she needs, up to two minutes.] 1. [XWordlist[5]]

2. [XWordlist[2]]
3. [XWordlist[9]]
4. [XWordlist[10]]
5. [XWordlist[3]]
6. [XWordlist[1]]
7. [XWordlist[8]]
8. [XWordlist[4]]
9. [XWordlist[6]]

Machine Translated by Google

10. [XWordlist[7]] 11.
No words recalled 12. Refusing
to recall
[conflict(11, 12, [11, 12])]

[Quote: Now I will read you the same series of words in a different order. Please follow me and read these ten words out loud one by one. Then I will ask you to recall these ten words. Are you ready?]

DC014Now tell me the words you can think of. transferPic("XWordlist","1 2 3 4 5 6 7 8 9 10")

[Interviewer's note: Read the words to the respondent at a slow, steady pace, and ask the respondent to repeat after you finish. Keep the frequency to about one word every two seconds. Only if the respondent is hearing impaired, you can show the respondent the word list and read the words while showing it. You should read the words out loud and ask the respondent to repeat the word before moving on to the next group of words. Give the respondent as much time as he or she needs, up to two minutes.]

1. [XWordlist[1]]
2. [XWordlist[2]]
3. [XWordlist[3]]
4. [XWordlist[4]]
5. [XWordlist[5]]
6. [XWordlist[6]]
7. [XWordlist[7]]
8. [XWordlist[8]]
9. [XWordlist[9]]
10. [XWordlist[10]] 11.
No words recalled 12. Refusing
to recall
[conflict(11, 12, [11, 12])]

DC015 Interviewer observation: Please indicate whether the following situations occurred during the whole process (multiple choices)

1. When the vocabulary is being
processed, it is interrupted 2. Other situations
(DC015_1) 3. None of the
above situations [conflict(3, [3])]

[Quote: The following 10 questions are about your feelings and behaviors in the past week. The answer to each question is the same, including rarely or not at all, not very much, sometimes, half the time, or most of the time. Please choose the appropriate answer]

DC016 I am troubled by little things.

[Interviewer's note: If the respondent does not understand the question, please continue to repeat the question to the respondent. Do not just answer "I don't know".]

1. Rarely or not at all (<1 day) 2. Not
very often (1-2 days) 3.
Sometimes or half the time (3-4 days) 4. Most
of the time (5-7 days) 997. Don't
know 999.
Refuse to answer

DC017 I have difficulty concentrating when doing things.

[Interviewer's note: If the respondent does not understand the question, please continue to repeat the question to the respondent. Do not just answer "I don't know".]

1. Little or no (<1 day)

Machine Translated by Google

2. Not too often (1-2 days)

3. Sometimes or half of the time (3-4 days) 4. Most

of the time (5-7 days) 997. Don't know

999. Refuse to

answer

DC018 I feel depressed. [Interviewer note:

If the respondent does not understand the question, please continue to repeat the question to the respondent. Do not just answer "I don't know"]

1. Rarely or not at all (<1 day) 2. Not

very often (1-2 days) 3.

Sometimes or half the time (3-4 days) 4. Most of the

time (5-7 days) 997. Don't know 999.

Refuse to answer

DC019 I find it very difficult to do anything.

[Interviewer's note: If the respondent does not understand the question, please continue to repeat the question to the respondent. Do not just answer "I don't know".]

1. Rarely or not at all (<1 day) 2. Not

very often (1-2 days) 3.

Sometimes or half the time (3-4 days) 4. Most of the

time (5-7 days) 997. Don't know 999.

Refuse to answer

DC020I am full of hope for the future.

[Interviewer's note: If the respondent does not understand the question, please continue to repeat the question to the respondent. Do not just answer "I don't know".]

1. Rarely or not at all (<1 day) 2. Not

very often (1-2 days) 3.

Sometimes or half the time (3-4 days) 4. Most of the

time (5-7 days) 997. Don't know 999.

Refuse to answer

DC021 I feel scared.

[Interviewer's note: If the respondent does not understand the question, please continue to repeat the question to the respondent. Do not just answer "I don't know".]

1. Rarely or not at all (<1 day) 2. Not

very often (1-2 days) 3.

Sometimes or half the time (3-4 days) 4. Most of the

time (5-7 days) 997. Don't know 999.

Refuse to answer

DC022 I don't sleep well. [Interviewer's

note: If the respondent does not understand the question, please continue to repeat the question to the respondent. Do not just answer "I don't know".]

1. Rarely or not at all (<1 day) 2. Not

very often (1-2 days) 3.

Sometimes or half the time (3-4 days)

Machine Translated by Google

4. Most of the time (5-7 days) 997. Don't
know 999. Refuse
to answer

DC023 I am very happy.

[Interviewer's note: If the respondent does not understand the question, please continue to repeat the question to the respondent. Do not just answer "I don't know".]

1. Rarely or not at all (<1 day) 2. Not very
often (1-2 days) 3. Sometimes
or half the time (3-4 days) 4. Most of the time (5-7 days)
997. Don't know 999. Refuse to answer

DC024 I feel lonely.

[Interviewer's note: If the respondent does not understand the question, please continue to repeat the question to the respondent. Do not just answer "I don't know".]

1. Rarely or not at all (<1 day) 2. Not very
often (1-2 days) 3. Sometimes
or half the time (3-4 days) 4. Most of the time (5-7 days)
997. Don't know 999. Refuse to answer

DC025 I feel I can't go on with my life. [Interviewer's note: If
the respondent does not understand the question, please continue to repeat the question to the respondent. Do not just answer "I don't know".]

1. Rarely or not at all (<1 day) 2. Not very
often (1-2 days) 3. Sometimes
or half the time (3-4 days) 4. Most of the time (5-7 days)
997. Don't know 999. Refuse to answer

DC026 Overall, are you satisfied with your life? Extremely satisfied, Very satisfied, Somewhat satisfied, Not very satisfied
Or not satisfied at all? 1.
Extremely satisfied
2. Very satisfied
3. Somewhat
satisfied 4. Not
quite satisfied 5. Not satisfied at all

DC027 Are you satisfied with the relationship between you and your children? Extremely satisfied, very satisfied, somewhat satisfied, not very satisfied, or not at all satisfied
Dissatisfied?

[Interviewer's note: This question is only asked to respondents who currently have
surviving children] 1.
Extremely satisfied 2.
Very satisfied 3.
Somewhat satisfied 4.
Not very satisfied 5. Not at all satisfied

Machine Translated by Google

6. No children now

[Quote: A few minutes ago I asked you to read the words on ten cards. Now I would like to ask you to try to recall these ten words. OK, now please tell me which of these ten words you still remember? The more the better.]

DC028Please select the words that the respondent successfully recalled.

[Interviewer's note: Please give the interviewee as much time as he needs, up to a maximum of two minutes.] 1. [XWordlist[1]]

2. [XWordlist[2]]
3. [XWordlist[3]]
4. [XWordlist[4]]
5. [XWordlist[5]]
6. [XWordlist[6]]
7. [XWordlist[7]]
8. [XWordlist[8]]
9. [XWordlist[9]]
10. [XWordlist[10]] 11. No words recalled 12. Refusing to recall [conflict(11, 12, [11, 12])]

DC029 Interviewer observation: Did the following situations occur during the interview? (Multiple choices) 1. The interviewee has poor eyesight

2. The interviewee has poor hearing and does not wear hearing aids 3. The interviewee wears hearing aids 4. The interviewee's hands are shaking, which affects some tests 5. The interviewee is disturbed by other matters or noise 6. The interviewee has emotional problems, resulting in poor questionnaire quality 7. Others, please specify **(DC029_1)** 8. None of the above

[conflict(8, [8])]

DC030 Interviewer observation: What language did the interviewer use during the interview? 1. Mandarin 2. Local dialect 3. Other dialects, please specify **(DC030_1)**

Auxiliary variable definition

XChroDisType Chronic disease type

add("XChroDisType", ["Hypertension", "Dyslipidemia (high or low blood lipids)", "Diabetes or elevated blood sugar (including impaired
ÿ glucose tolerance and elevated fasting blood sugar)", "Cancer and other malignant tumors (excluding mild skin cancer)",
ÿ "Chronic lung diseases such as chronic bronchitis or emphysema, cor pulmonale (excluding tumors or cancer)", "Liver disease (except fatty liver, tumors or cancer)",
ÿ "Heart disease (such as myocardial infarction, coronary heart disease, angina pectoris, congestive heart failure and other heart diseases)", "Stroke",
ÿ "Kidney disease (excluding tumors or cancer)", "Gastric disease or digestive system disease (excluding tumors or cancer)", "Emotional and mental problems", "Memory-
ÿ related diseases (Alzheimer's disease, brain atrophy)", "Parkinson's disease", "Arthritis or rheumatism", "Asthma (non-lung disease)"])

XFAgePossibility asks for the specific value of living to a certain age

Machine Translated by Google


```
if (!greater("XRAge", "65") && !equal("XRAge", "65")) {
        add("XFAgePossibility", "75")

} if ((greater("XRAge", " 65") || equal("XRAge", "65")) && !greater("XRAge", "69")) {
        add("XFAgePossibility", "80")

} if ((greater("XRAge", " 70") || equal("XRAge", "70")) && !greater("XRAge", "74")) {
        add("XFAgePossibility", "85")

} if ((greater("XRAge", " 75") || equal("XRAge", "75")) && !greater("XRAge", "79")) {
        add("XFAgePossibility", "90")

} if ((greater("XRAge", " 80") || equal("XRAge", "80")) && !greater("XRAge", "84")) {
        add("XFAgePossibility", "95")

} if ((greater("XRAge", " 85") || equal("XRAge", "85")) && !greater("XRAge", "89")) {
        add("XFAgePossibility", "100")

} if ((greater("XRAge", " 90") || equal("XRAge", "90")) && !greater("XRAge", "94")) {
        add("XFAgePossibility", "105")

} if ((greater("XRAge", " 95") || equal("XRAge", "95")) && !greater("XRAge", "99")) {
        add("XFAgePossibility", "110")

} if (greater("XRAge", "100") || equal("XRAge", "100"))
        { add("XFAgePossibility", "115")
}
```

XPsyActTypePhysical activity typeadd ("XPsyActType",

```
["Very strenuous activity that requires a lot of physical effort (strenuous activity that makes you breathe faster, such as carrying heavy objects, digging, farming, aerobic exercise,
ÿ     fast cycling, cycling with cargo, etc.)", "Moderate physical activity (moderate physical activity that makes you breathe faster than usual, such as carrying light objects,
ÿ     riding a bicycle at a normal speed, mopping the floor, practicing Tai Chi, and walking)", "Light physical
ÿ     activity such as walking (walking includes walking from one place to another at work or at home, and other walks you take for leisure, sports, exercise, or entertainment)"])
```

XSocType Social Activity Type

```
if (selected("DA038","1"))
          { add("XSocType[1]", "Visiting, socializing with friends")

} if (selected("DA038","2")) {
        add("XSocType[2]", "Play mahjong, chess, cards, go to the community activity room")

} if (selected("DA038","3"))
        { add("XSocType[3]", "Provide assistance to relatives, friends or neighbors who do not live with you")

} if (selected("DA038","4"))
        { add("XSocType[4]", "Dancing, fitness, qigong, etc.")

} if (selected("DA038","5"))
        { add("XSocType[5]", "Participate in community activities")

} if (selected("DA038","6")) {
        add("XSocType[6]", "Volunteering, charity, or caring for a sick or disabled person who does not live with you")

} if (selected("DA038","7"))
        { add("XSocType[7]", "Go to school or take a training course")

} if (selected("DA038","8"))
        { add("XSocType[8]", "Other social activities")
}
```

XHelperSelect Does anyone help

```
if (equal("DB002", "1") || equal("DB004", " 1") || equal( "DB006", "1") || equal("DB008", "1") || equal("DB010", "1") ||
ÿ     equal("DB013", " 1") || equal( "DB015", "1") || equal("DB017", "1") || equal( "DB019", "1") || equal("DB021", "1") ||
ÿ     equal("DB023", "1")) { add("XHelperSelect", "1") } else { add(" XHelperSelect", "0")



}
```

XHelperChild provides a list of children's names to help

Machine Translated by Google


```
for (var i1 = 1; i1 <= 25; i1++)
        { add("XHelperChild[i1]", value("XChildPanAliveName[i1]"))

} for (var i1 = 26; i1 <= 35; i1++)
        { add("XHelperChild[i1]", value("DB026_1[i1]"))
}
```

XSibName generates a list of sibling names

```
for (var i1 = 1; i1 <= 15; i1++) { if (!
        empty("ZSibName[i1]"))
                { add("XSibName[i1]", pre("XRName")+"ÿÿÿ"+pre("ZSibName[i1]"))
        }

} for (var i1 = 1; i1 <= 15; i1++) { if (!
        empty("ZSibNameS[i1]")) {
                add("XSibName["+(i1+15)+"]", pre("XRName")+"spouse's brothers and sisters"+pre("ZSibNameS[i1]"))
        }
}
```

XHelperSib provides the name of the sibling to help

```
for (var i1 = 1; i1 < 31; i1++)
        { add("XHelperSib[i1]", value("XSibName[i1]"))

} for (var i1 = 31; i1 <= 40; i1++)
        { add("XHelperSib[i1]", value("DB028_1[i1]"))
}
```

XHelperNum provides the number of helpers

```
add("XHelperNum", "0") if
(selected("DB024", "1"))
        { add("XHelperNum", value("XHelperNum")+1)
        add("XHelper["+value("XHelperNum ")+"]", "spouse")

} if (selected("DB024", "5"))
        { add("XHelperNum", value("XHelperNum")+1)
        add("XHelper["+value("XHelperNum")+"]", "Other relatives")
}

if (selected("DB024", "6"))
        { add("XHelperNum", value("XHelperNum")+1)
        add("XHelper["+value("XHelperNum")+"]", "Hire personnel")
}

if (selected("DB024", "7"))
        { add("XHelperNum", value("XHelperNum")+1)
        add("XHelper["+value("XHelperNum")+"]", "Volunteer ")
}

if (selected("DB024", "8"))
        { add("XHelperNum", value("XHelperNum")+1)
        add("XHelper["+value("XHelperNum")+"]", "Nursing home staff")
}

if (selected("DB024", "9"))
        { add("XHelperNum", value("XHelperNum")+1)
        add("XHelper["+value("XHelperNum")+"]", "Staff of home-based elderly care service agencies")
}

if (selected("DB024", "10"))
        { add("XHelperNum", value("XHelperNum")+1)
        add("XHelper["+value("XHelperNum")+"]", "Community" )
}

if (selected("DB024", "11"))
        { add("XHelperNum", value("XHelperNum")+1)
        add("XHelper["+value("XHelperNum")+"]", "Other personnel")

} if (selected("DB025", "1"))
        { add("XHelperNum", value("XHelperNum")+1)
        add("XHelper["+value("XHelperNum")+"]", "Father ")
}
```

Machine Translated by Google


```
if (selected("DB025", "2"))
    { add("XHelperNum", value("XHelperNum")+1)
    add("XHelper["+value("XHelperNum")+"]", "Mother" )

} if (selected("DB025", "3"))
    { add("XHelperNum", value("XHelperNum")+1)
    add("XHelper["+value("XHelperNum")+"]", "Father-in-law /father-in-law")

} if (selected("DB025", "4"))
    { add("XHelperNum", value("XHelperNum")+1)
    add("XHelper["+value("XHelperNum")+"]", "Mother-in-law")

} for (var i1 = 1; i1 < 26; i1++) { if
    (selected("DB026", i1) && selected("DB027[i1]", "1"))
        { add("XHelperNum", value(" XHelperNum")+1)
        add("XHelper["+value("XHelperNum")+"]", value("XChildPanAliveName[i1]")+"I")

    } if (selected("DB026", i1) && selected("DB027[i1]", "2"))
        { add("XHelperNum", value("XHelperNum")+1)
        add("XHelper["+value ("XHelperNum")+"]", value("XChildPanAliveName[i1]")+"spouse")

    } if (selected("DB026", i1) && selected("DB027[i1]", "3"))
        { add("XHelperNum", value("XHelperNum")+1)
        add("XHelper["+value ("XHelperNum")+"]", value("XChildPanAliveName[i1]")+"child")
    }

} for (var i1 = 26; i1 < 36; i1++) { if
    (selected("DB026", i1) && selected("DB027[i1]", "1"))
        { add("XHelperNum", value(" XHelperNum")+1)
        add("XHelper["+value("XHelperNum")+"]", value("DB026_1[i1]")+"myself")

    } if (selected("DB026", i1) && selected("DB027[i1]", "2"))
        { add("XHelperNum", value("XHelperNum")+1)
        add("XHelper["+value ("XHelperNum")+"]", value("DB026_1[i1]")+"spouse")

    } if (selected("DB026", i1) && selected("DB027[i1]", "3"))
        { add("XHelperNum", value("XHelperNum")+1)
        add("XHelper["+value ("XHelperNum")+"]", value("DB026_1[i1]")+"child")
    }

} for (var i1 = 1; i1 < 31; i1++) { if
    (selected("DB028", i1) && selected("DB029[i1]", "1")) { add("XHelperNum",
        value(" XHelperNum")+1)
        add("XHelper["+value("XHelperNum")+"]", value("XSibName[i1]")+"I")

    } if (selected("DB028", i1) && selected("DB029[i1]", "2"))
        { add("XHelperNum", value("XHelperNum")+1)
        add("XHelper["+value ("XHelperNum")+"]", value("XSibName[i1]")+"spouse")

    } if (selected("DB028", i1) && selected("DB029[i1]", "3"))
        { add("XHelperNum", value("XHelperNum")+1)
        add("XHelper["+value ("XHelperNum")+"]", value("XSibName[i1]")+"child")
    }

} for (var i1 = 31; i1 < 41; i1++) { if
    (selected("DB028", i1) && selected("DB029[i1]", "1")) { add("XHelperNum",
        value(" XHelperNum")+1)
        add("XHelper["+value("XHelperNum")+"]", value("DB028_1[i1]")+"myself")

    } if (selected("DB028", i1) && selected("DB029[i1]", "2"))
        { add("XHelperNum", value("XHelperNum")+1)
        add("XHelper["+value ("XHelperNum")+"]", value("DB028_1[i1]")+"spouse")

    } if (selected("DB028", i1) && selected("DB029[i1]", "3"))
        { add("XHelperNum", value("XHelperNum")+1)
        add("XHelper["+value ("XHelperNum")+"]", value("DB028_1[i1]")+"child")
    }
}
```

XHelper helper identity/name

see above

XSelectNum determines whether the number of selected helpers is more than 7

Machine Translated by Google


```
add("XSelectNum", "0")
for (var i1 = 1; i1 < 99; i1++) { if
    (selected("DB032", i1))
        { add("XSelectNum", value("XSelectNum")+1 )
    }
}
```

The main helpers selected by **XHelpList**

```
for (var i1 = 1; i1 < value("XHelperNum")+1; i1++) { if
    ( greater("XHelperNum", "7") && !equal("XHelperNum", "7") && selected("DB032 ", i1) ) { add("XHelpList[i1]",
        value("XHelper[i1]"))
    }

} for (var i1 = 1; i1 < value("XHelperNum")+1; i1++) { if ( !
    greater("XHelperNum", "7") )
        { add("XHelpList[i1]", value("XHelper [i1]"))
    }
}
```

XHelperCurrent Whether there is a caregiver now

```
if (greater("XHelperNum", "0"))
    { add("XHelperCurrent", "1") }
else
    { add("XHelperCurrent", "0")
}
```

XWordlist generates a scale for word tests

```
if (!empty("XMainR"))
    { add("XWordlist", ["River", "Book", "Eyes", "Tile House", "Pole", "Stamp", "Motorcycle", "Grass", "Egg", "Chairman"])

} if (empty("XMainR"))
    { add("XWordlist", ["sky", "newspaper", "arm", "building", "stick", "ticket", "car", "flower", "milk", "prime minister"])
}
```

XWordRecallBR whether to do an instant phrase recall test

```
if (!selected("DC012", "13") && !selected("DC012", "12") && !empty("DC012")) {
    add("XWordRecallBR", "1") }
else
    { add("XWordRecallBR", "0")
}
```

Machine Translated by Google

F Work and Retirement

Machine Translated by Google

F. Proxy Mode Confirmation

Proxy_7 Interviewer record: For the work module, do you use the proxy questionnaire mode? 1. Yes

2. No

FA. Job Overview (I)

[Quote: Next I would like to know about [XRName] 's recent work.]

FA001In the past year, has [XRName] done farm work for more than 10 days? Planting crops, managing fruit trees, collecting agricultural and forestry products, raising fish,

Fishing, raising livestock, or selling home-grown agricultural products at the market are all considered farm work. [Interviewer's

note: The definition of agricultural activities in the work module and the income module is the same. The difference is that the work module asks the interviewee whether he or she does it, while

the income module asks whether the household

members

do it.] 1. Yes 2. No

FA002 Does [XRName] do farm work for his own family, or does he earn a salary working for other farmers or farms, or both? (Multiple choice question, options

1. Work

for your own family 2.

Earn a salary by working for other farmers/ farms

FA004 Let's consider non-agricultural employment. Did [XRName] work at least one hour last week?

[Interviewer Note: The definitions of being employed and

non-agricultural self-employed are the same in the work module and the income module. The difference is that the work module asks the respondents whether they do it

themselves, while the income module asks whether the

household

members do it.] 1. Yes 2. No

FA005 Is [XRName] employed? Has he not worked in the last week? Is he currently on temporary leave, sick leave, or other vacation?

On-the-job training?

1. Yes 2.

No

FA007 Will [XRName] be able to return to his original job within a certain time or within 6 months?

1. Yes 2.

No

FA008 Does the original unit or employer still pay [XRName] ? 1. Yes 2. No

FA009 Does [XPrefixMainJob] [XRName] currently have more than one non-farm job?

[Interviewer's note: odd jobs one after another do not count as multiple jobs.] 1.

Yes, at least two jobs 2. No,

only one job

Machine Translated by Google

FA010 [XPrefixMainJob] [XRName] has multiple jobs. What is the main job of [XRName] ?

[XRName] What is the job with the longest working hours? Is this main job [XSuffixMainJob] doing non-agricultural work for a salary, doing business as an individual, or helping out in a family business without salary? We will focus on this main job later. (optional) [Interviewer's note: Priority will be given to jobs that have not

been terminated.] 1. Non-agricultural employment

2. Engaging in

individual or private economic activities 3. Helping

out in a family business without salary 4.

[XWorkTypeFarmEmployed]

FA011 [XRName] Is this job a part-time job for a salary, a personal business, or an unpaid helper for a family business? (optional

1. Non-

agricultural

employment 2. Engage in individual or private

economic activities 3. Help family business activities without salary

FA. Work Overview (II)

FA013 During the last interview, we learned that [XRName] was not working (including farming) at [ZIWTime] . So from then until now, when did [XRName] start

working (including farming)? [Interviewer Note: If the respondent answered that he was working

during the last interview, please select "Negative last round record". If the respondent confirmed that he

was not working during the last interview, but was unable to answer the month he started working, he can fill in "-1" for the month.]

1. ________ [hc([ZIWYear, 2020], , ÿ), sc([2018, 2020], , ÿ)] **(FA013_1)** year [hc([1, 12], , ÿ1)] **(FA013_2)** month995 . Negative last round

record: I was working at that

time999. Refuse to answer

FA014 When we say [XRName] was not working at that time, we mean that he had not done farm work in the year before [ZIWTime] and had not done non-farm work in the week before.

When we say [XRName] is currently working, we mean that he had done farm work in the past year or had done non-farm work in the week before or was on vacation. Please

confirm again, was [XRName] really working at that time? [Interviewer's note: If the respondent thinks that

the answer about currently working is inaccurate, please go back to the previous question to make corrections. If the respondent

thinks that our record is correct, please go back to the previous question and answer the time when he started working

again.] 1. Respondent confirmed that he was

working at that time

997. Don't know 999. Refuse to answer

FA015 What was the main reason for [XRName] to start working?

1. To earn more income, or produce more things for personal use (e.g. having nothing to do at home, or growing vegetables for

personal use) 2. Health reasons (e.g. previously in poor health, now recovered) 3.

Family reasons (e.g. previously caring for family members, now no longer) 4.

Exercise 5. No

special reason, just want to work, not for money or production 6. Others, please specify **(FA015_1)**

____

FA016 During our last visit, we learned that [XRName] was working at [ZIWTime]. As I said earlier, farm work, wage work, business, and helping in the family

business are all considered work. So from then to now, when was [XRName]?

Machine Translated by Google

Did you stop working when you last interviewed? [Interviewer's note: If the respondent answers that he was not working when you last interviewed him, please select "Negative record of last round". If the respondent confirms that he was working when you last interviewed him but cannot answer the month when he stopped working, you can fill in "-1" for the month.]

1. _________ [hc([ZIWYear,XIWYear], , ÿ), sc([2018,XIWYear], , ÿ)] **(FA016_1)** year , ÿ1)] **(FA016_2)** month ([1, 12], 995. Negative last _________ [hc

at that time round record: I was not working

999. Refuse to answer

FA017 When we say [XRName] was working, we mean that he had done farm work in the year before [ZIWTime] or had done non-farm work in the week before. When we say [XRName] is not working currently, we mean that he had not done farm work in the past year and had not done non-farm work in the week before. Please confirm again, was [XRName] really not working at that time? [Interviewer's note: If the respondent thinks that the answer about not working currently is inaccurate, please go back to the previous question to make corrections. If the respondent thinks that our record is correct, please go back to the previous question and answer the time when he stopped working again.]

1. Respondent confirmed that he was not working at that time 997. Don't know 999. Refuse to answer

FA018 Sorry, we recorded it wrong before. [ZIWTime] [XRName] is not working. So, what was the last time [XRName] worked ? **(FA018_1)** year [hc([1, 12],

When did it stop? [hc([1950, 2020], **18_2)** months [Interviewer's note: If _________ , ÿ1)] **(FA0**

the respondent cannot answer the month, please fill in "-1".]

FA019 What is the main reason for [XRName] to stop working? 1. Passive reasons (such as being dismissed by the company, the store was demolished, and the land was expropriated)

2. The business made little money or the salary was too low (including renting out the farmland) 3. Work reasons other than income (such as the road is too far, dislike) 4. Health reasons (such as poor health) 5. Family reasons (such as needing to take care of family members, family members not allowing work, and getting married and having children) 6. Retirement reasons (leaving the job due to retirement age) 7. Temporary suspension of work (such as self-employed business operators temporarily suspending business due to something) 8. Others, please specify **(FA019_1)**

FB. Self-employed agricultural workers

[Quote: Next I would like to know about the farm work that [XRName] does for his family.]

FB001Where did [XRName] do farm work in the past year ?

[Interviewer's note: If the respondent does not know the street and community, he/she can choose as appropriate] 1. The same village/community where you live: [XRResidenceFull] 2. Other villages/communities in the same district or county [XRResidenceCounty] where you live : **(FB001_1)** Township/town/street/village/community

3. Outside the county of residence: **(FB001_2)** Province/City/District/County 4. Regions where _________ **(FB001_3)** Township/town/street/village/community

none of the above options apply (Hong Kong, Macao, Taiwan and overseas)

999. Refuse to answer

FB002 What type of farm work does [XRName] do? (Multiple choices are allowed)

1. Planting industry (cereals, yam, cotton, linen, sugar, tobacco, vegetables, fruits, Chinese medicinal herbs, tea and grass)

Machine Translated by Google

2. Forestry (breeding and afforestation, wood and bamboo harvesting and transportation, forest management) 3. Animal husbandry (raising livestock, poultry, etc., hunting) 4. Fisheries (aquaculture, aquatic fishing) 5. Professional and auxiliary agricultural, forestry, animal husbandry and fishery activities 997. Don't know [conflict(997, [997])]

FB003 What kind of farm work does [XRName] do for his family? 1. Production labor 2. Business management 3. Machine operation 4. Purchasing and sales 5. Others

FB005 In the past year, how many months did [XRName] do farm work for his family? [hc([1, 12], ______ , ÿ)] months

FB006 In the past year , how many days per week did [XRName] do farm work for his family? [hc([1, 7], ______ , ÿ)] day

[Interviewer's note: If the number of days per month is less than four, and the average number of days per week is less than one, fill in "1".]

FB007In the past year , how many hours did [XRName] usually work on the farm for his family? [hc([1, 24], , ÿ)] hours ______ , ÿ), sc([1, 16],

FC. Employment (I)

[Quote: Next, I want to know about the [XWorkTypeFarmEmployed] job that [XRName] just mentioned . If [XRName] is doing odd jobs and has a different employer, the following employers refer to the current or most recent employer.]

FC001 Does [XRName] get his salary from his employer/boss, or does he need to receive it through a dispatching company or individual contractor? (optional

1. Work unit (can be an individual and work for it) 2. Labor dispatch unit 3. Individual contractor (as a labor intermediary, only pays wages)

FC002 What type of work unit (or employer) does [XRName] work for? [XFC002] 1. Government department 2. Public institution 3. Non-profit organization, such as a society, association, or society 4. Enterprise 5. Self -employed 6. Farmer 7. Residential household 8. Other, please specify ______ **(FC002_1)** 997. Don't know

FC003 I remember that the [XWorkplace] of [XRName] at [ZIWTime] was [ZFD003] during the last visit . Is the [XWorkplace] of [XRName] still the same now?

[Interviewer's note: If the unit/employer does not have departmental divisions, please fill in "None" for the department name.]

Machine Translated by Google

1. Yes, the name of the department you are currently in: (Example 1: Farm Restaurant; Example 2: Spinning Production Workshop) ________ (FC003_2)

2. No

995. Negation of last round record: It is the same [XWorkplace] , but the name was wrong last time

---

Example 2: Beijing Huaxin Garment Co., Ltd.; Example 3: Boss Zhang) The name of the ________

department is: (Example 1: Farm Restaurant; Example 2: Spinning Production Workshop) [Interviewer ________ **(FC003_2)**

Note: If the unit/employer does not have a department, fill in "None" for the department name]

FC004 Where is [XRName] working now?

[Interviewer's note: If the respondent does not know the street and community, he/she can

choose as appropriate] 1. The same village/community where the residence is located:

[XRResidenceFull] 2. Other villages/communities in the same district or county [XRResidenceCounty] where the residence is located : **(FC004_1)** Township/town/street/village/community

3.

Outside the county where you live: (FC004_2) Province/City/District/County 4. Regions where ________ **(FC004_3)** Township/town/street/village/community

none of the above options apply (Hong Kong, Macao, Taiwan and overseas) 999.

Refuse to answer

FC005 What industry does [XRName] 's [XWorkplace] mainly belong to? In other words, what products does [XWorkplace] currently manufacture or what services does it provide?

(Example 1: Providing catering services on campus; Example 2: Manufacturing yarn fabrics) [Interviewer's note: Please fill in the ________

form according to the requirements of the "Industry and Occupation Filling Specifications"]

FC006 Is [XRName] a civil servant now? 1. Yes 2. No

FC008 Is [XRName] a regular employee now? 1. Yes 2. No

FC011 What kind of occupation does [XRName] belong to? In other words, what does [XRName] do now? (Example 1: restaurant pastry chef; Example 2: production line

winding worker) [Interviewer's note: Please fill in the form ________

according to the requirements of the "Industry and Occupation Filling Specifications"]

FC012 Does [XRName] manage others?

1. Yes 2.

No

FC016 Has [XRName] signed a written employment contract with [XEmployer] ? 1. Yes 2. No

FC017 How long is the term of [XRName] 's current labor contract?

[Interviewer Note: If the respondent answers that it is a fixed term but cannot remember the specific year and month, please select fixed term and fill

1. Fixed term [hc([0, 50], 2) months 2. No fixed term in "-1" for the year and month.] , ÿ1)] **(FC017_1)** Year Zero [hc([0, 12], , ÿ1)] **(FC017_**

3.

Employment contract with

a term of completing a certain work task

Machine Translated by Google

997. Don't know999.

Refuse to answer

FC018 I remember that [XRName] started working at [XWorkplace] in [ZFD011_1] . Is this correct? If not,

If the interviewee is unable to answer the year and month, please fill in

"-1". 1. Correct 2. If the interviewee is unable to answer the year and

month,

please fill in "-1". The correct start time of the job is [hc([1950,XIWYear], , ÿ1)] **(FC018_**

1) Year [hc([1, 12], 999. Refused , ÿ1)] **(FC018_2)** month

to answer

FC019 When did [XRName] start working at [XWorkplace] ? **19_1)** year [hc([1, 12], , ÿ1)] ________ [hc([1950,XIWYear], , ÿ1)] **(FC0**

(FC019_2) month [Interviewer's note: If the respondent cannot answer the

year and month, please fill in "-1".]

FC025 In the past year, how many months did [XRName] work in this job? Paid vacation and unpaid sick leave do not need to be deducted. , ÿ)] months

________ [hc([1, 12],

[Interviewer's note: Exception: For this job, the interviewee has been on vacation for the past year and has not received any salary, so fill in "1" for the number of working months,

days per week, and hours per week.]

FC026 In the past year, how many days a week did [XRName] usually work at this job? This does not include paid vacations, sick leave without salary

deductions, and rest days. [hc([1, 7], , ÿ)] days [Interviewer's note: If there

are less than four days per month, and less than one day per week on average, fill in "1".]

FC027During the past year, how many hours did [XRName] usually work per day in this job? Working hours do not include lunch breaks , ÿ)] hours

hours, but includes overtime. ________ [hc([1, 24], , ÿ), sc([1, 16],

FC. Employment (II)

FC032 How is [XRName] 's salary mainly paid? Is it paid regularly, by project, by performance, or in other ways? If it is paid regularly, is it paid annually,

monthly, weekly, daily, or hourly? If the company owes wages and bonuses or has just started working, please tell us the wages that [XRName]

should get. (optional) [Interviewer's note: Wages that should be received but not received refer to those that

can be received in the future. If a certain wage or bonus is no longer possible to be claimed, it cannot be counted as wages that should be received.]

1. Received

annually 2. Received

monthly 3. Received

weekly 4. Received

daily 5. Hourly

wages 6. By project

7. By

performance, including piecework wages

8. Others, please specify **(FC032_1)** 997. Don't know

999. Refuse to

answer

FC033 [XRName] has worked in this job for [FC025] months in the past year . Since [XRName] 's salary is mainly paid annually, and he may not have

received it yet, how much salary should he have received from [XEmployer] for the corresponding [FC025] months? Please give me the bonus

Machine Translated by Google

All kinds of income are included, including tips, red envelopes, and gifts received at work. [hc([0, 10000000], , 1), sc([1000, 1000000], , ÿ1), ub([ÿ1], [10000, 30000, 50000, 100000, 200000])] yuan [Interviewer note: If the respondent cannot answer, please fill in "-1".]

FC034How much salary did [XRName] receive from [XEmployer] last month ? [XPrefixMonthlyWage] Please include all income including bonuses, tips, red envelopes, and gifts received at work. [hc([0, 10000000], ,), sc([100, 100000], , ÿ1), ub([ÿ1], [500, 1000, 2500, 5000, 10000])] yuan [Interviewer's note: If the respondent cannot answer, please fill in "-1".] ÿ1

FC035 How much did [XRName] get from [XEmployer] last week ? Please include all income including bonuses, tips, red envelopes, and gifts received at work. [hc([0, 10000000], , ÿ1), sc([100, 100000], ,), ub([ÿ1], [100, 300, 500, 1000, 2000])] yuan [Interviewer's note: If the respondent cannot answer, please fill in "-1".] ÿ1

FC036 How much does [XRName] usually get from [XEmployer] every day ? Please include all kinds of income such as bonuses, including tips, red envelopes, and gifts received at work. [hc([1, 10000000], , ÿ1), sc([100, 100000], , ÿ1), ub ([ÿ1], [20, 50, 100, 200, 500])] yuan [Interviewer's note: If the respondent cannot answer, please fill in "-1".]

FC037 How much does [XRName] usually get per hour from [XEmployer] ? Please include all income including bonuses, tips, red envelopes, and gifts received at work. [hc([1, 10000000], , ÿ1), sc([10, 100000], , ÿ1), ub([ÿ1], [10, 30, 50, 100, 200])] yuan [Interviewer's note: If the respondent cannot answer, please fill in "-1".]

FC038So , how much did [XRName] get from [XEmployer] last month? [XPrefixMonth-lyWage] Similarly, please include all kinds of income such as bonuses, including tips, red envelopes, and gifts received at work. [hc([0, 10000000], , ÿ1), sc([100, 100000], , ÿ1), ub([ÿ1], [500, 1000, 2500, 5000, 10000])] yuan [Interviewer's note: If the respondent cannot answer, please fill in "-1".]

FC039 In the past year, how much other bonuses did [XRName] receive from [XEmployer] ? This refers to bonuses that are not paid together with salary, such as year-end bonuses, holiday bonuses, etc. [hc([0, 10000000], , ÿ1), sc([0, 1 000000], , ÿ1), ub([ÿ1], [1000, 3000, 5000, 10000, 20000])] yuan [Interviewer note: If the respondent cannot answer, please fill in "-1".]

FC040 Some people have to pay individual income tax, pension insurance, medical insurance, housing provident fund or other miscellaneous Have these fees been deducted from the salary and bonus amounts mentioned above?

1. Need to pay, have they been deducted
2. Need to pay, have not been deducted
3. No need to pay taxes, insurance or other miscellaneous fees

997. Don't know
999. Refuse to answer

How much is **FC041** [XPrefixFC041] ? [Interviewer's note: If the respondent has difficulty reporting the specific amount of tax and insurance, he or she can fill in the first option as the percentage of salary, accurate to ten digits.]

1. Equivalent to [hc([1, 50], , ÿ)] **(FC041_1)** % of salary [hc([100, 100000], , ÿ)]
2. ________ **(FC041_2)** yuan/month [hc([100, 100000], , ÿ)] **(FC041_3)**
3. ________ yuan/year

Machine Translated by Google

997. Don't know

FC042 What benefits does [XRName] [XEmployer] provide? (Multiple choices are allowed) How much is each benefit worth each month?

[Interviewer's note: The value refers to the net value, which is the difference between the market price of the welfare item and the actual cost to the employee. For example, if an employee pays 500 yuan per month to live in the company dormitory, and the market rent of a dormitory with the same conditions is 2,000 yuan, then the net value is 1,500 yuan. If the respondent cannot estimate the net value, please fill in "-1".]

1. Meals: Free breakfast, lunch, dinner and meal allowance, net value of about [hc([10, 10000], , ÿ1), ub([ÿ1], [100, 500, 1000])] **(FC042_1)**

2. Transportation: Unit-provided cars, shuttle buses, and transportation subsidies, with a net value of approximately [hc([10, 50000], , ÿ1), ub([ÿ1], [100, 500, 1000])] **(FC042_2)**

3. Accommodation: free or lower-than-market-price dormitory and housing subsidy, net value of about [hc([10, 100000], , ÿ1), ub([ÿ1], [500, 2000, 5000])] **(FC042_3)**

4. The total net value of the regular distribution of in-kind and other subsidies is about [hc([10, 100000], , ÿ1), ub([ÿ1], [10 0, 500, 1000])] **(FC042_4)** yuan 5. No benefits except salary and bonus (exclusive option)

999. Refusal to answer

[conflict(5, 999, [5, 999])]

FD. Non-agricultural self-employment

[Quote: Next I want to know about the [XRName] [XSelfEmplType] I just mentioned .]

FD002 What is the current address of [XRName] [XSelfEmplName] ?

[Interviewer's note: If the respondent does not know the street and community, he/she can choose as appropriate] 1. The same village/community where the residence is located: [XResidenceFull] 2. Other villages/communities in the same district or county [XResidenceCounty] where the residence is located : **(FD002_1)** Township/town/street/village/community

district

3. Outside the county of residence: **(FD002_2)** Province/City/District/County 4. Regions **(FD002_3)** Township/town/street/village/community where none of the above options apply (Hong Kong, Macao, Taiwan and overseas)

999. Refuse to answer

FD003 What does [XRName] [XSelfEmplName] mainly do now? In other words, what industry do you belong to? What products do you produce or what business activities do you engage in? (Example 1: manufacturing yarn fabrics; Example 2: selling clothing) [Interviewer's note: Please fill in the form according to the requirements of the "Industry and Occupation Filling Standards"]

FD004 [XRName] [XSelfEmplName] How many people do you usually employ now? [hc([0, 9999], , ÿ)] people (fill in if you don't employ anyone) "0")

FD007 In the past year, how many months did [XRName] work in the [XSelfEmplVerb] business? [hc([1, 12], , ÿ)]

moon

FD008 In the past year, how many days a week did [XRName] usually work in the [XSelfEmplVerb] business? , ÿ)] days c([1, 7], [h

[Interviewer's note: If there are less than four days a month, or less than one day a week on average, fill in "1".]

FD009During the past year, how many hours did [XRName] usually work per day ? , ÿ)] hours [h

c([1, 24], , ÿ), sc([1, 16],

Machine Translated by Google

FE. Non-main job

[Quote: Just now you said that [XPrefixMainJob] [XRName] has multiple jobs, which means that in addition to the main job, [XRName] also has [XSuffixMainJob] other jobs.]

FE001 Now let's consider all the other jobs. Taken together, how many days a week does [XRName] usually work? [hc([0, 7], , ÿ), sc((0, 7], , ÿ)] day

FE002For these "all other tasks", how many hours per day does [XRName] usually do? [hc([1, 24], , ÿ)] hours , ÿ), sc([1, 16],

FF. Job search and employment

FF001 In the past year, how many days did [XRName] work? Farm work, wage work, business, and helping out in the family business are all considered work. This question refers to the days [XRName] actually worked and went to work, excluding rest days. [hc([, ÿ1), ub([ÿ1], [10, 50, 100, 150, 250])] days (If the respondent cannot answer, please fill in "-1". If from 0, 366], you have never worked, fill in "0") **[Interviewer Note:** 1. It was previously learned that if the respondent's work day distribution remains constant throughout the year, the respondent has about [XHhldFarmDays] days of self-employment in agriculture, about [XMainJobDays] days of current main job, and about [XSideJobDays] days of current side job. These numbers are for reference only and cannot be directly added up. Note that the respondent may have multiple jobs on the same day. There may be overlap in working days for different jobs. [XFF001]

2. The answer should be accurate to ten days. 3. If the interviewer finds that there is a problem with the previous answers to the work hours, please return to the corresponding sub-module to make changes.

FF002 Does [XRName] currently have a side job that earns a small income?

1. Yes
2. No

FF003Has [XRName] looked for a job in the past month ? 1. Yes 2. No

FF004 At what age do you plan to stop working, that is, stop all activities for the purpose of earning money, stop helping in family business activities, and do not plan to engage in activities that are more strenuous than recreational work in the future?

Please provide an approximate age. [Interviewer's note: The age of stopping work should be greater than or equal to the respondent's current age [XRAge] years old] 1. Plan to stop working at [hc([XRAge, 120], , ÿ1), sc([60, 100], , ÿ1)] **(FF004_1)** years old 2. Plan to stop working in [hc([0, 50], , ÿ1)] **(FF004_2)** years old 3. Work as long as health allows 997. Don't know 999. Refuse to answer

FF005 Imagine that if there was no epidemic, [XRName] might have an idea of the age at which he would stop working, but because of the epidemic, the situation may have changed. So, has the time when [XRName] planned to stop working been delayed or brought forward due to the epidemic, or has it remained unchanged? [Interviewer's note: If there is a change of several months, it will be rounded up to one year if it is less than a year. If the respondent cannot answer the specific number of years, he can fill in "-1". Whether there is an epidemic or not, as long as health allows, he is prepared to work all the time. In this case, select "no change".]

1. Earlier by [hc([1, 50], , ÿ1)] **(FF005_1)** years 2. Later by [hc([1, 50], , ÿ1)] **(FF005_2)** years 3. No change 997. Don't know 999. Refuse to answer

FG. Working during the pandemic

[Quote: Next, I would like to know the impact of the epidemic on [XRName]' s work.]

FG001During the epidemic, some units adopted remote working methods such as online or telephone conferences, online office, and online sales. [XRName] Have you ever worked remotely during the pandemic, staying home to do work for your boss or company?

1. Yes 2. No

FG002There is a situation, has [XRName] ever experienced it? There was a period of time during the epidemic [XVCNotInQuarantine] , [XRName] 's boss or company did not start work, so I can still get part of the salary even if I don't work?

[Interviewer Note: If the respondent cannot answer, please fill in "-1".] 1. Yes, in this case, the average salary is equivalent to [hc([1, 100], 1)% of the normal salary. This situation , ÿ1)] **(FG002_** lasted for [hc([1, 50], 2. Yes, in this case, the average salary is higher , ÿ1)] **(FG002_2)** week than the normal salary. 3. No, there is no such situation

FG003During the epidemic, some people were restricted from going to the fields to do farm work. Imagine if there was no epidemic, [XRName] might do farm work for his family, but because of the epidemic, the situation may have changed. So, from the end of the Chinese New Year to now, has the number of days [XRName] worked on the farm for his family increased, decreased, or had no impact due to the epidemic? Farm work refers to the agricultural activities just introduced, including agriculture, forestry, animal husbandry, and fishery, as well as selling agricultural products produced by the family. [Interviewer's note: If the respondent cannot answer the number of weeks, please fill in "-1".] 1. Will not do farm work for his family whether there is an epidemic or not 2. Worked less [hc([1, 40], , ÿ1)] **(FG003_1)** weeks 3. Worked more [hc([1, 40], , ÿ1)] **(FG003_2)** weeks 4. No change 999. Refuse to answer

FG004During the epidemic, some people who work have delayed returning to work or lost their jobs. Imagine again, if there is no epidemic, [XRName] may go to work to earn a salary, but because of the epidemic, the situation may have changed. So, from the end of the Chinese New Year to now , has the number of days [XRName] worked to earn a salary increased, decreased, or had no impact due to the epidemic? [Interviewer Note: If the respondent cannot answer the number of weeks, please fill in "-1".] 1. Would not go to work to earn a salary whether there is an epidemic or not 2. Worked less [hc([1, 40], , ÿ1)] **(FG004_1)** weeks 3. Worked more [hc([1, 40], , ÿ1)] **(FG004_2)** weeks 4. No change 999. Refuse to answer

FG005During the epidemic, some shops could not open, and even if they did, they had no business. Imagine if there was no epidemic, [XRName] might be an individual business owner, but because of the epidemic, the situation may have changed. So, from the end of the Chinese New Year to now,

Machine Translated by Google

[XRName] Did the number of days for business increase, decrease, or no effect? Business includes self-employment and unpaid help for family businesses, but does not include agricultural production and management. [Interviewer Note: If the respondent cannot answer the number of weeks, please fill in "-1".] 1. Will not engage in non-agricultural self-employment with or without the epidemic 2. Work less [hc([1, 40], , ÿ1)] **(FG005_1)** weeks 3. Work more [hc([1, 40], , ÿ1)] **(FG005_2)** weeks 4. No change 999. Refuse to answer

FG008 During the epidemic, some people only worked half a day. If there was no epidemic, they might have worked full days. For [XRName] , the situation may have changed because of the epidemic. So, from the end of the Chinese New Year to now, in the working months, has the epidemic increased, decreased, or had no effect on [XRName] 's weekly working hours? [Interviewer's note: The days when the respondent does not work should not be considered here, and the reduction in days and hours cannot be counted repeatedly. If the impact of the epidemic causes the respondent to be away from work all day, this situation should be recorded as a reduction in days and recorded in the previous three questions. If the respondent cannot answer the specific number of hours, please fill in "-1".] 1. No change 2. Reduced by [hc([1, 40], , ÿ1)] **(FG008_1)** hours 3. Increased by [hc([1, 40], , ÿ1)] **(FG008_2)** hours 4. Did not work after the Chinese New Year 999. Refuse to answer

FG009Did [XRName] receive unemployment insurance during the epidemic ? 1. Yes 2. No

FG010 How many months did [XRName] receive unemployment insurance? The average monthly unemployment insurance benefit is [hc([1, 10], , ÿ1)] **(FG010_1) yuan .** [Interviewer's note: If the respondent cannot answer, please fill in "-1".]

FH. Retirement procedures

FH001 Has [XRName] completed the retirement procedures, including early retirement and internal retirement? Retirement refers to retirement from government and enterprise institutions. Retirement also includes the retirement of flexible employment personnel who have participated in basic pension insurance. [Interviewer note: Starting to receive urban resident insurance, new rural insurance, and urban and rural resident pension insurance does not count as completing retirement procedures.]

1. Yes 2. No

FH002 [XRName] Is it normal retirement, early retirement, internal retirement first and then formal retirement, or internal retirement and then formal retirement in the future? (optional)

1. Normal retirement
2. Early retirement
3. Internal retirement and then formal retirement 4. Internal retirement but not yet formal retirement

FH003 In which year and month did you complete your official retirement formalities? [hc([1950,XIWYear], , ÿ1), sc([ZIWYear,XIWYear], , ÿ1)] **(FH003_1)** year [Interviewer's [hc([1, 12], , ÿ1)] **(FH003_2)** month note: If the respondent cannot answer, please fill in “-1”.]

Machine Translated by Google

FH004 In which month and year will you complete the formal retirement procedures? [hc([XIWYear, 2050], , ÿ1)] **(FH004_1)** year

______ [hc([1, 12], , ÿ1)] **(FH004_2)** Months

[Interviewer's note: If the respondent cannot answer, please fill in "-1".]

Auxiliary variable definition

Is **XWorking** employed?

```
if (equal("FA001", "1") || equal ("FA004", " 1") || equal ("FA007", " 1") || equal("FA008", "1")) { add("XWorking", "1") } else
    { add("XWorking", "0")

}
```

Is **XEmployed** self-employed?

```
if (equal("FA010", "2") || equal ("FA010", " 3") || equal( "FA011", " 2") || equal("FA011", "3")) { add("XEmployed", "0") }
    else if (equal("FA010", "
1") || equal("FA010", "4") || equal("FA011", "1") || selected("FA002", "2")) { add("XEmployed", "1")

}
```

XFGSample asked whether to work during the epidemic

```
if (equal("XWorking","1")) { //Currently working
    add("XFGSample","1") }
else if (equal("FF003","1")) { //Currently looking for a jobadd
    ("XFGSample","1") } else
if (equal("FF005","1") || equal("FF005","2")) { //The epidemic has changed retirement
    expectationsadd ("XFGSample","1")
} else if (equal("FA016_1","2020")) { // Stop working after January 1st
    add("XFGSample","1") }
else
    { add("XFGSample","0")
}
```

XPrefixMainJob Main job question prefix

```
if (selected("FA002","1"))
    { add("XPrefixMainJob", "Except doing farm work for my family,")
}
```

XSuffixMainJob Main job question suffix

```
if (selected("FA002","2"))
    { add("XSuffixMainJob", "earn wages doing farm work")
}
```

XEmployer salary payment unit

```
if (equal("WC001","2"))
    { add("XEmployer", "Dispatching
Unit") } else if (equal("WC001","3"))
    { add("XEmployer", "Contractor")
} else if (equal("WC001","1") && greater("WC002","4"))
    { add("XEmployer",

"Employer") } else { add("XEmployer", "Workplace")
}
```

XWorkplace work unit

```
if (greater("WC002","4")))
    { add("XWorkplace",

"Employer") } else { add("XWorkplace", "Workplace")
}
```

Machine Translated by Google

XSelfEmplName Non-agricultural self-employed/unpaid family helper job name

```
if (equal("FA010","2") || equal("FA011","2") )
        { add("XSelfEmplName","Business")
        add("XSelfEmplType","Individual/private
        business")

add("XSelfEmplVerb","Do") } else
        { add("XSelfEmplName","Family business with helpers")
        add("XSelfEmplType","Family business with unpaid helpers") add("XSelfEmplVerb","Helper")
}
```

XSelfEmplTypeNon -agricultural self-employment/unpaid home helper business nature

see above

XSelfEmplVerb Non-agricultural self-employment/unpaid home help business verb

see above

XFC002 WC002 Wording

```
if (equal("FC001","2")) {
        add("XFC002", "Note, it is the employer, not the dispatching
unit") } else if (equal("FC001","3"))
        { add("XFC002", "Note, it is the employer, not the contractor")
}
```

XFC003 WC003 Wording

```
if (greater("FC002","4"))
        { add("XFC003", "What is the name of the work
unit") }
        else { add("XFC003", "What is the name of the employer")
}
```

XWorkJustStarted Whether the current employment started less than one year or one month ago

```
if ((equal("FC019_1" , "2020") && greater("FC019_2", value("XIWMonth")-2)) || (equal("FC018_1", "2020") && ÿ greater("FC018_2" ,
 value("XIWMonth")-2))) { // Less than a month
        add("XWorkJustStarted", "1") } else if
((equal("FC019_1", "2020") || (equal(" FC019_1", "2019") && greater("FC019_2",
ÿ       value("XIWMonth")-1))) || (equal("FC018_1", "2020") || (equal("FC018_1", "2019") && greater("FC018_2", value("XIWMonth") -1))))
ÿ       { add("XWorkJustStarted", "2") }
        else { add("XWorkJustStarted", "0")              // Less than a year

                                                         // One year ago
}
```

XMainJobDays Main working days

```
if (equal("XEmployed","1"))
        { add("XMainJobDays", value("FC026")*52)
} else if (equal("XEmployed","1")) {
        add("XMainJobDays", value("FD008")*52) } else

{ add("XMainJobDays", "0")
}
```

XHhldFarmDays Agricultural self-employment days

```
add("XHhldFarmDays", value("FB006")*value("FB005")*4) if
(empty("XHhldFarmDays"))
{ add("XHhldFarmDays", "0") }
```

XSideJobDaysNumber of non-main working days

```
add("XSideJobDays", value("FE001")*52) if
(empty("XSideJobDays"))
        { add("XSideJobDays", "0")
}
```

Machine Translated by Google

XFF001 FF001 Interviewer Attention

```
if (equal("XWorking","0")) { add("XFF001",
    "Also note that respondents may have worked in non-agricultural jobs earlier in the past year, even though they are not currently working.")
} else if (empty("XEmployed")) {
    add("XFF001", "Also note that although the respondents are not currently working in non-agricultural jobs, they may have worked in non-agricultural jobs earlier in the past year.")
} else if (greater("12", "FC025")) { add("XFF001", "Also,
    please note that although the respondents are currently working in non-agricultural jobs, they have been doing this job for less than 12 months in the past year.
     They may not have been working or were doing other different jobs during the rest of the time." )
}
```

XGetJob No Job ÿ Job

```
if (equal("ZWorking","0") && equal("XWorking","1")) { add("XGetJob",
    "1") } else { add("XGetJob",
"0")

}
```

XQuitJob job ÿ no job

```
if (equal("ZWorking","1") && equal("XWorking","0")) { add("XQuitJob",
    "1") }else { add("XQuitJob",
"0")

}
```

XPrefixMonthlyWage: How to use the wording when asking about last month's salary for people who have just started working

```
if (equal("XWorkJustStarted", "1"))
    { add("XPrefixMonthlyWage", "You have just started working. If you have not received salary yet, please tell me how much salary you will get next month.")
}
```

XPrefixFC041 FC041 wording

```
if (equal("FC040", "1"))
    { add("XPrefixFC041", "Then it will be

deducted") } else { add("XPrefixFC041", "Then the salary will be deducted again")
}
```

XWorkTypeFarmEmployedMain jobAgricultureEmployedOptions

```
if (selected("FA002","2"))
    { add("XSuffixMainJob", "Agricultural Employment")
}
```

Machine Translated by Google

G. Income and Expenditure

Machine Translated by Google

G1 Household income and expenditure

G1. Proxy mode confirmation

proxy_12 Interviewer records: Is the proxy questionnaire model used for the household income and housing modules?

1. Yes

2. No

GB. Income of other household members

GB001 Which of the following household members is the household finance respondent? The financial respondent must be familiar with the household's economic situation.

[Interviewer Note: If you want to use a proxy in the household expenditure and income module, please apply for/use the existing [XFLHHFinancialNameList] personal proxy, and then select [XFLHHFinancialNameList] in this question .] 1-25. [XHHMemberName[ ]]

26. [XMainR]

27. [XMainRS]

[Quote: Please make sure no one else is present. Now we would like to ask about the income of other household members in your family. Your answers will be kept strictly confidential and will only be used for academic research.]

GB002[ ] Did [XHHMemberName[ ]] receive any salary in the past year , including bonuses and various subsidies, excluding retirement and resignation?

And the internal retirement salary? The salary here comes from all the employed work.

1. Yes

2. No 997.

Don't know 999.

Refuse to answer

GB003[ ] In the past year, how much money did [XHHMemberName[ ]] receive in total? [hc((0, ÿ), , ÿ1), sc((0, 240000 , ÿ1), ub([ÿ1], [5000, 10000,

], 30000, 50000, 100000])] yuan

[Interviewer's note: If the respondent refuses to answer or forgets, please fill in "-1".]

GB004[ ] Has the salary of [XHHMemberName[ ]] mentioned above been deducted from various insurances, income tax, housing provident fund or other

Miscellaneous

expenses? 1.

Yes 2. No 997. Don't

know 999. Refuse to answer

GB005[ ] [XHHMemberName[ ]] [XGB005Text[ ]] Individual income tax, various insurances, and housing provident fund deducted or paid

How much is the total amount of money or other

miscellaneous expenses? [Interviewer Note: If the respondent refuses to answer or forgets, select the first option and fill in

1. ________ "-1".] [hc((0, ÿ), , ÿ1), sc((0, 10000], , ÿ1), ub([ÿ1], [300, 500, 1000, 2000, 3000])] (G

B005_1[i]) yuan/

________ month [hc((0, ÿ), , ÿ), sc((0, 100000], , ÿ)] **(GB005_2[i])** yuan/year 2. 3. Or

equivalent to salary [hc((0, 100], 4. No, 0 yuan , ÿ)] **(GB005_3[i])** %

Machine Translated by Google

GB006[ ] In the past year, did [XHHMemberName[ ]] receive any of the following transfer payments? Note that after the outbreak, the epidemic subsidies received by individuals also belong to transfer payments, so this must be filled in (multiple choices are allowed).

[Interviewer Note: If the respondent refuses to answer or forgets, fill in "-1".] 1.

Retirement or pension, including government agency and public institution retirement pensions, enterprise employee basic pension insurance, enterprise supplementary pension insurance, retirement or internal retirement compensation, rural or urban and rural or urban resident pension insurance, commercial pension insurance, life insurance, land acquisition pension insurance, etc., received [hc((0, ÿ), , ÿ1), sc((0, 100000], , ÿ1)] **(GB00 6_1[i])** yuan

2. Unemployment benefits: [hc((0, ÿ), , ÿ1), sc((0, 50000], , ÿ1)] **(GB006_2[i])** yuan received 3. Pension cards or coupons: [hc((0, ÿ), , ÿ1), sc((0, 50000], , ÿ1)] **(GB006_3[i])** yuan received 4. Pension subsidies for the elderly: [hc((0, ÿ), , ÿ1), sc((0, 50000], , ÿ1)] **(GB006_4[i]) yuan received**

Yuan

5. Work-related injury insurance includes work-loss allowance, disability allowance, etc., and the amount received is [hc((0, ÿ), , ÿ1), sc((0, 50000], , ÿ1)] **(GB006_5[i])** yuan.

6. Old-age allowance for only children, and the amount received is [hc((0, ÿ), , ÿ1), sc((0, 50000], , ÿ1)] **(GB006_6[i]) yuan.**

Yuan

7. Medical assistance, received [hc((0, ÿ), , ÿ1), sc((0, 100000], , ÿ1)] **(GB006_7[i])** yuan 8. Other government subsidies to individuals, excluding minimum living allowance, five guarantees, special hardship and poverty subsidies, please indicate **(GB006_8_1[i]) ,** received [hc((0, ÿ), , ÿ1), sc((0, 100000], , ÿ1)] **(GB006_8[i])** yuan 9. Other transfer payments from society to individuals, such as social donations, please indicate **(GB006_9_1[i]) ,** received

[hc((0, ÿ), , ÿ1), sc((0, 100000], , ÿ1)] **(GB006_9[i])** yuan 10. None of the above [conflict(10, [10])]

GB007[ ] Consider that if there were no epidemic, [XHHMemberName[ ]] might have some salary income, but because of the epidemic, the situation may have changed. So, from the Spring Festival to now, has the epidemic increased or decreased [XHHMemberName[ ]]'s salary income, or has it had no effect? [Interviewer's note: If the respondent refuses to answer or forgets, fill in "-1".] 1. Decreased, decreased [hc((0, ÿ), , ÿ1), sc((0, 50000], , ÿ1), ub([ÿ1], [1000, 3000, 5000 , 10000, 20000])] **(GB007_1[i])** yuan

2. Increase, increase [hc((0, ÿ), , ÿ1), sc((0, 50000], , ÿ1), ub([ÿ1], [1000, 3000, 5000 , 10000, 20000])] **(GB007_2[i])** yuan 3. No change

GB008[ ] In the past year, did [XHHMemberName[ ]] receive any of the following transfer payments? Please note that after the outbreak, the epidemic subsidies received by individuals also belong to transfer payments, so you must fill in this field (multiple choices are allowed). [Interviewer Note: If the respondent refuses to answer or forgets, fill in "-1".]

7. Medical assistance, received [hc((0, ÿ), , ÿ1), sc((0, 50000], , ÿ1)] **(GB008_7[i])** yuan 8. Other government subsidies to individuals, excluding minimum living allowance, five guarantees, special hardship and poverty subsidies, please indicate **(GB008_ 8_1[i]) ,** received [hc((0, ÿ), , ÿ1), sc((0, 50000], , ÿ1)] **(GB008_8[i])** yuan 9. Other transfer payments from society to individuals, such as social donations, please indicate **(GB008_9_1[i]) ,** received [hc((0, ÿ), , ÿ1), sc((0, 50000], , ÿ1)] **(GB008_9[i])** yuan 10. None of the above [conflict(10, [10])]

GC. Household agricultural income

Machine Translated by Google

[Quote: Below, we will ask some questions about your family's agricultural income and expenditure. In the personal questionnaire, agricultural work is about the individual respondent, but here it is at the household level. Even if the respondent does not engage in agricultural activities, if there are members of the household engaged in agricultural activities, it is considered that the household is engaged in agricultural activities.]

GC001 In the past year, have any members of [XMainR] 's household, including [XFLHHNameList] , engaged in any agricultural activities such as planting crops, managing fruit trees, collecting agricultural and forestry products, raising fish, fishing, raising livestock, or selling their own agricultural products in the market?

1. Yes

2. No

GC002 In the past year, which household members were engaged in these agricultural activities? (Multiple selections are allowed) 1-25. [XHHMemberName[ ]]

26. [XMainR]

27. [XMainRS]

GC003 Have the household members of [XMainR] , including [XFLHHNameList] , engaged in planting or forestry in the past year, including planting flowers, trees, vegetables and various crops, planting mushrooms, fungus and other understory crops, planting tea, or collecting wild agricultural and forestry products?

1. Yes 2. No

GC004 In the past year, how much net income did [XMainR] get from the above planting/forestry production and collection of wild agricultural and forestry products? Did they make money or lose money? Net income is the value of output minus input. Output includes the part sold, consumed and stored, and input includes seeds, fertilizers, pesticides, irrigation, hired workers, rented machines, plastic film, rented land from others, fuel, transportation fees, processing fees, packaging fees, management fees, etc.

[Interviewer Note: If the respondent refuses to answer or forgets, fill in "-1".] 1. Make money, net income is [hc((0, ÿ), , ÿ1), sc((0, 100000], , ÿ1), ub([ÿ1], [2000, 5000, 10000, 50000, 100000])] **(GC004_1)** yuan

2. Loss, loss of [hc((0, ÿ), , ÿ1), sc((0, 100000], , ÿ1), ub([ÿ1], [2000, 5000, 10000, 50000, 100000])] **(GC004_2)** yuan 3. No profit or loss

GC005 Have any household members of [XMainR] , including [XFLHHNameList] , raised livestock or aquatic products, or caught wild animals or aquatic products in the past year? Livestock includes poultry, livestock (such as chickens, ducks, cattle, pigs and sheep), etc., and aquatic products include fish, etc.

1. Yes

2. No

GC006 In the past year, how much net income did [XMainR] get from livestock and aquatic product farming activities and wild animal and aquatic product fishing activities? Did they make money or lose money? Net income is the value of output minus input. Output includes the income from the sale of final products and by-products in the market and the value of the portion consumed by the family. Input includes feeding fees, medical expenses, grazing fees, animal house fence fees, labor fees, etc. [Interviewer Note: If the respondent refuses to answer or forgets, fill in "-1".] 1. Make money, net income is [hc((0, ÿ), , ÿ1), sc((0, 100000], , ÿ1), ub([ÿ1], [2000, 5000, 10000, 50000, 100000])] **(GC006_1)** yuan 2. Loss, loss of [hc((0, ÿ), , ÿ1), sc((0, 100000], , ÿ1), ub([ÿ1], [2000, 5000, 10000, 50000, 100000])] **(GC006_2)** yuan 3. No profit or loss

GC007 Consider that if there were no epidemic, the household members of [XMainR], including [XFLHHNameList] , might have some agricultural income, but because of the epidemic, the situation may have changed. So, from the Spring Festival to now, the epidemic has made [XMainR]

Machine Translated by Google

Did the net income from agricultural production increase, decrease, or have no effect?

[Interviewer's note: If the respondent refuses to answer or forgets, fill in "-1".]

1. Decreased, decreased by [hc((0, ÿ), , ÿ1), sc((0, 10000], , ÿ1), ub([ÿ1], [200, 500, 1000, 5 000, 10000])] **(GC007_1)** yuan 2. Increased,

increased by [hc((0, ÿ), , ÿ1), sc((0,

10000], , ÿ1), ub([ÿ1], [200, 500, 1000, 5 000, 10000])] (GC007_1) yuan

000, 10000])] **(GC007_2)** yuan 3. No

change

GD. Income from self-employment and private enterprise

GD001 In the past year, did the household members of [XMainR] , including [XFLHHNameList] , engage in some self-employment or start a business?

Private enterprises?

[Interviewer's note: Respondents were asked about self-employment in the personal questionnaire, but that was at the individual level. Self-employment and private enterprises at the household level are only required to involve household members, and the scope is wider than that of the personal questionnaire.]

1. Yes
2. No

GD002 In the past year, [XMainR] household members, including [XFLHHNameList] , engaged in several self-employed activities or started ,

How many private enterprises? [hc([1, 25],

GD003 In the past year, which household members were engaged in this [GD002] individual business or private enterprise? (Multiple choices are allowed)

1-25. [XHHMemberName[ ]]

26. [XMainR]

27. [XMainRS]

GD004 Can you accurately estimate how much net profit or loss [XMainR] 's family made from this [GD002] business activity in the past year? If non-household members participate in the business activity, only calculate the net income of the household members. Don't forget to consider the following costs: depreciation of fixed capital, energy, housing and equipment rental fees, raw materials, transportation costs, marketing, wages, taxes and miscellaneous expenses. Fixed capital

investment in the past year should not be included in the cost. [Interviewer

Note: If the respondent refuses to answer or forgets, fill in "-1". ÿ 1. Make money, net profit is [hc((0, ÿ), , ÿ1), sc((0, 500000], , ÿ1),

ub([ÿ1], [5000, 10000, 50 000, 100000, 200000])]

(GD004_1) yuan 2. Lose money, lose [hc((0, ÿ), , ÿ1), sc((0, 500000], , ÿ1), ub([ÿ1], [5000, 10000, 5000

0, 100000, 200000])] **(GD004_2)** yuan 3. No profit

or loss 999. Refuse

to answer

GD005 Consider that if there were no epidemic, the household members of [XMainR], including [XFLHHNameList] , might have some self-employed and private enterprise income, but because of the epidemic, the situation may have changed. So, from the Spring Festival to now, has the epidemic increased, decreased, or had no effect on the net profit of [XMainR] 's self-employed and private enterprises? [Interviewer's

note: If the respondent refuses to answer or forgets, fill in "-1".] 1. Decreased,

decreased [hc((0, ÿ), , ÿ1), sc((0, 50000], , ÿ1), ub([ÿ1], [500, 1000, 5000,

10000, 20000])] **(GD005_1)** yuan

2. Increase, increase [hc((0, ÿ), , ÿ1), sc((0, 50000], , ÿ1), ub([ÿ1], [500, 1000, 5000,

10000, 20000])] **(GD005_2)** yuan 3. No

change

Machine Translated by Google

GD006 Due to the impact of the epidemic, have the self-employed and private enterprises of [XMainR] changed? 1. My family did not have self-employed and private enterprises before and after the epidemic . 2. My family had self-employed and private enterprises before and after the epidemic . 3. My family did not have self-employed and private enterprises before the epidemic, but they were established after the epidemic . 4. My family had self-employed and private enterprises before the epidemic, but they were closed after the epidemic.

GD007 Due to the impact of the epidemic, from the Spring Festival to now, have [XMainR]'s self-employed and private enterprises received the following subsidies or grants? (Multiple choices are allowed) [Interviewer's note: If the respondent refuses to answer or forgets, fill in "-1".] 1. Government loan, loan of [hc((0, ÿ), , ÿ1), sc((0, 500000], , ÿ1)] **(GD007_1)** yuan, interest The information is [hc([0, 100], , ÿ1)] **(GD007_2)** % per year

2. Factory/store rent reduction, from the Spring Festival to now, the total reduction is [hc((0, ÿ), , ÿ1), sc((0, 50000], , ÿ1)] **(GD007_3)**

3. Tax reductions: From the Spring Festival till now, the total amount of tax reductions is [hc((0, ÿ), , ÿ1), sc((0, 50000], , ÿ1)] **(GD007_4)** Yuan

4. Others, please specify **(GD007_5) ,** from the Spring Festival to now, the total amount of [hc((0, ÿ), , ÿ1), sc((0, 50000], , ÿ1)] **(GD007_6)** yuan 5. None of the above

[conflict(5, [5])]

GE. Household public transfer income

GE001 Has [XMainR] or any household member of [XMainR] , including [XFLHHNameList] , ever been or is currently a five-guarantee household/elderly, minimum living allowance household/elderly, extremely poor household/elderly, or impoverished household? (Multiple choices are allowed) 1. Five-guarantee household/elderly

2. Minimum living allowance household/elderly 3. Extremely poor household/elderly 4. Poverty-stricken household registered in archives 5. Other types of impoverished households, please specify **(GE001_1)** 6. None of the above

[conflict(6, [6])]

GE002[ ] [XMainR] family or any of its members, was or is [XPoorHHName[ ]]? Since when? If If it has been interrupted, please use the oldest start time as the start time. 1. Now, the start time is [hc((1980, 2020], , ÿ)] **(GE002_1[i])** years 2. It used to be but is no longer, the start time is [hc((1980, 2020], , ÿ)] **(GE002_2[i])** years

GE003[ ] Which of the following household members received [XPoorHHName[ ]] subsidies from the government? There may be non-household members and household members Received the subsidy from the government together, non-household members are not considered here (multiple selections are allowed).

1-25. [XHHMemberName[ ]]

26. [XMainR]

27. [XMainRS]

GE004[ ] In the past year, how much subsidy did the household members of [XMainR] , including [XFLHHNameListPoor[ ]] from [XPoorHH-Name[ ]] just mentioned , receive in total? If the subsidy is received by non-household members, only the household member's portion needs to be calculated. Physical goods need to be converted into cash. [hc([0, ÿ), , ÿ1), sc((0, 20000], , ÿ1), ub([ÿ1], [500, 100 0, 5000, 10000, 20000])] yuan

Machine Translated by Google

[Interviewer's note: If the respondent refuses to answer or forgets, please fill in "-1".]

GE006 [XMainR] Has your family received any of the following government subsidies, social donations or compensation at the household level in the past year? This does not include the subsidies for the five-guarantee households, low-income households, extremely poor households or poverty-stricken households mentioned earlier. Note that after the outbreak of the epidemic, the epidemic subsidies received at the household level also belong to transfer payment income, so you must fill it in here (multiple choices are allowed). [Interviewer Note: If the respondent refuses to answer or forgets, fill in "-1".]

1. Returning farmland to forest: How much? [hc((0, ÿ), , ÿ1), sc((0, 20000], , ÿ1)] **(GE006_1)** yuan 2. Agricultural subsidies: How much? [hc((0, ÿ), , ÿ1), sc((0, 20000], , ÿ1)] **(GE006_2)** yuan 3. Pensions for dependents of workers injured in work: How much? [hc((0, ÿ), , ÿ1), sc((0, 20000], , ÿ1)] **(GE006_3)** yuan

4. After a major disaster, the government's donations and subsidies, including relief funds and disaster relief funds, etc.: together with the value of in-kind assistance, the total is How many? [hc((0, ÿ), , ÿ1), sc ((0, 20000], , ÿ1)] **(GE006_4)** yuan . 5. Social donations and subsidies, including food, clothing, school bags, and crowd-funded medical expenses, etc.: together with the value of in-kind assistance, the total is How many? [hc((0, ÿ), , ÿ1), sc((0, 20000], , ÿ1)] **(GE006_5)** yuan . 6. Land acquisition compensation: how much? [hc((0, ÿ) , , ÿ1), sc((0, 20000], , ÿ1)] **(GE006_6)** yuan . 7. Housing demolition compensation: how much? [hc((0, ÿ), , ÿ1), sc((0, 20000], , ÿ1)] **(GE006_7)** yuan 8. Other subsidies, please specify **(GE006_8_1) :** How much? [hc((0, ÿ), , ÿ1), sc((0, 2000 0], , ÿ1)] **(GE006_8)** yuan

9. No government or social subsidies or donations received

[conflict(9, [9])]

GE007 [XMainR] Did your family receive any compensation for production insurance in the past year? How much did you receive? (If not, fill in 0)

Insurance compensation includes agricultural insurance compensation, etc. [hc([0, ÿ), , ÿ1), sc([0, 20000], , ÿ1)] yuan

[Interviewer's note: If the respondent refuses to answer or forgets, fill in "-1".]

[Quote: This section asks about the various transfer payments received by households and their members from public institutions. Public transfer payments are welfare expenditures, such as government subsidies to households receiving five guarantees and extremely poor households.]

GE008Due to the impact of the epidemic, did [XMainR] and [XMainR] 's household members, including [XFLHHNameList] , receive any government subsidies for the epidemic? Including government subsidies for agricultural epidemics given to households, excluding subsidies for self-employed/private enterprises, such as reductions in store/factory rents and tax exemptions. In-kind subsidies must be converted into cash. [Interviewer's note: If the respondent refuses to answer or forgets, fill in "-1".] 1. Yes, the total amount of various epidemic subsidies is [hc((0, ÿ), , ÿ1), sc((0, 20000], , ÿ1), ub([ÿ1], [500, 1000, 5000, 10000, 20000])] **(GE008_1)** yuan 2. No

GE009 [XMainR] Whether the family has installed solar panels on the roof of the house, agricultural greenhouse or other places to generate electricity for their own use, Sell excess electricity to the national grid?

1. Yes 2. No

GE010 [XMainR] When did you install solar panels? [hc((1990, 2020], , ÿ1)]

[Interviewer's note: If the respondent refuses to answer or forgets, please fill in "-1".]

GE011 In the past year, how much income did [XMainR] earn from photovoltaic power generation? Including the value of electricity consumed by the family and the electricity sold to the national grid. [hc([0, ÿ), , ÿ1), sc((0, 20000], , ÿ1)] yuan [Interviewer's note: If the respondent refuses to answer or forgets, fill in "-1".]

Machine Translated by Google

GE012 Does [XMainR] have collectively allocated land? Does [XMainR] rent out land to others? If so, how much did you rent out in the past year?

If you do not have land or do not rent out land, please fill in "0". [hc([0, ÿ), , ÿ1), sc ([0, 20000], , ÿ1), ub([ÿ1], [500, 1000, 5000, 10000, 20000])] yuan [Interviewer's note: If the respondent refuses to answer or forgets, fill in "-1".]

GE013 Does [XFamilyRAndS] own any property? Has the property under [XFamilyRAndS] been rented out? How much rent did you receive in the past year? If you do not own any property or have not rented it out, please fill in "0". If the property is owned by multiple people, please calculate the rent that [XFamilyRAndS] should receive based on the property rights. [hc([0, ÿ), , ÿ1), sc([0, 20000], , ÿ1), ub([ÿ1], [5 00, 1000, 5000, 10000, 20000])] yuan [Interviewer's note: If the respondent refuses to answer or forgets, fill in "-1".]

GE014 In addition to renting out houses or land, how much rent did [XMainR] household receive from renting out other household assets, such as trees, use of fixed capital, durable goods, or livestock in the past year? [Interviewer Note: If the respondent refuses to answer or forgets, fill in "-1".] 1. Yes, the rent in the past year is [hc([0, ÿ), , ÿ1), sc((0, 20000], , ÿ1)] **(GE014_1)**

Yuan 2. No rental

997. Don't know

999. Refuse to answer

GE015[ ] You said that [XMainR]' s family or its members were once [XPoorHHName[ ]], starting from [GE002_2[ ]], then When does it end? [hc([GE002_2[i], 2020], , ÿ)]

GF. Household living expenses

[Quote: Please read aloud "This section is the living expenses of all household members, including [XFLHHNameList] ."]

GF001On average, how much do the household members of [XMainR], including [XFLHHNameList] , spend in total per month? This includes rent, food, clothing, communication expenses, utilities, fuel, services, entertainment, daily necessities, and medical expenses.

______ [hc((0, ÿ), , ÿ), sc((0, 20000], , ÿ)]

GF002 We would like to know the food expenses of [XMainR]' s family in the past week. Are you responsible for buying food for your family?

1. Yes
2. No

GF003 Who is responsible for buying food for [XMainR]' s family?

1-25. [XChildAliveName[ ]]

26-35. [XHHOtherMemberName2[ ]]

36. [XMainR]

37. [XMainRS] 38. Babysitter

39. Neighbor

40. Others, please specify **(GF003_1)**

[**Quote:** If possible, GF004-GF010 should be answered by the person responsible for purchasing food for the household.]

GF004In the past week, excluding guests, how many people usually ate at home? [hc([0, ÿ), , ÿ), sc([1, 1 0], , ÿ)] people

Machine Translated by Google

GF005 In the past week, how many meals did [XMainR]'s guests eat at your home, calculated by person? [hc([0, ÿ), , ÿ), sc ([0, 100], , ÿ)] people

[Quote: The following series of questions are about the living expenses of [XMainR] 's family. These expenses include the living expenses of all household members. As long as they are members of the household, their expenses outside the home must also be included, such as accommodation and food expenses.]

GF006 In the past week, how much money did [XMainR]'s family spend on food, not including wine, wedding banquets, dining out, cigarettes, Drinks, etc? _______ [hc([0, ÿ), , ÿ), sc((0, 6000], , ÿ)] element

GF007 Does [XMainR] produce its own agricultural products, including grain crops, meat, eggs, aquatic products, oil, vegetables and fruits, tobacco, alcohol, beverages and dairy products, processed

foods,

seasonings, etc.? 1. Yes 2. No

GF008 In the past week, [XMainR] How much is the value of the agricultural products produced by the family if sold in the market? [hc([0, ÿ), , ÿ), sc((0, 6000], , ÿ)] yuan

GF009 In the past week, how much did the household members of [XMainR] , including [XFLHHNameList] , spend on dining out, excluding Setting up a wedding banquet? [hc([0, ÿ), , ÿ), sc([0, 3000], , ÿ)]

GF010 In the past week, how much money did the household members of [XMainR] , including [XFLHHNameList] , spend on cigarettes, alcohol, etc.? _______ [hc([0, ÿ), , ÿ), sc([0, 3000], , ÿ)] element

GF011 Now we want to know how much [XMainR] , whose household members include [XFLHHNameList] , spent on the following items in the past month

1. Postal and

telecommunication expenses, including telephone, mobile phone, Internet access, mail, etc. [hc([0, ÿ), , ÿ1), sc((0, 5000], , ÿ1)] **(GF011_1)**

2. Water and electricity costs [hc([0, ÿ), , ÿ1), sc((0, 5000], , ÿ1)] **(GF011_2)** yuan 3. Fuel costs, including coal,

coal products, firewood, charcoal, natural gas, liquefied gas, etc. [hc([0, ÿ), , ÿ1), sc ((0, 5000], , ÿ1)] **(GF011_3)** yuan 4. Expenses for

nannies, hourly workers, servants, etc. [hc([0,

ÿ) , , ÿ1), sc((0, 5000], , ÿ1)] **(GF011_4)** yuan 5. Local transportation costs [hc([0, ÿ), , ÿ1), sc((0, 5000], , ÿ1)] **(GF011_5)** yuan 6. Daily

necessities, including toiletries, household items, kitchen and bathroom supplies, decorative items, etc. [hc([0, ÿ), , ÿ1),

sc((0, 5000], , ÿ1)] **(GF011_6)** yuan 7. Cultural and entertainment expenses, including books, newspapers, magazines, CDs, movie and

drama tickets, dance halls and Internet cafes

[hc([0, ÿ), , ÿ1), sc ((0, 5000], , ÿ1)] **(GF011_7)** yuan [Note to interviewers: If there is no corresponding item, use "0" yuan. If you can't

remember, please ask the respondent to

estimate a number. If the respondent refuses to answer or forgets, fill in "-1".]

GF012 Will the nanny/part-time worker/maid hired by [XMainR] take care of the following people? (Multiple choices

are allowed) 1. Elderly

people in the household 2.

Children in the household 3. Others, please specify

(GF012_1) 4. None of the above

[conflict(4, [4])]

GF013 Now we want to know how much [XMainR] household, including [XFLHHNameList] , spent on the following items in the past year:

1. Clothing

consumption [hc([0, ÿ), , ÿ1), sc([0, 100000], , ÿ1)] **(GF013_1)** yuan

Machine Translated by Google

2. Family travel expenses, including travel expenses for going back and forth from home, travel expenses for cars and hotels, etc.
[hc([0, ÿ), , ÿ1), sc([0, 100000], , ÿ1)] **(GF013_2)** yuan 3. Family
heating expenses, referring to central heating [hc([0, ÿ), , ÿ1), sc([0, 100000], , ÿ1)] **(GF013_** 3) yuan 4. Purchase, repair and spare parts
of furniture, durable consumer goods and electrical appliances. Furniture, durable consumer goods and electrical appliances include
refrigerators, washing machines, televisions, computers and high-end musical instruments such as pianos. [hc([0, ÿ), , ÿ1), sc([0, 100000], , ÿ1)] **(GF013_4)** yuan
5. Education and training expenses, including tuition fees, training fees, etc. [hc([0, ÿ), , ÿ1), sc([0, 100000], , ÿ1)]
(GF013_5) yuan 6.
Medical expenses, including direct and indirect. Note: Indirect medical expenses refer to transportation costs, nutrition costs, family care costs, etc.
incurred due to medical treatment. It does not include the part that has been reimbursed by medical insurance [hc([0, ÿ), , ÿ1), sc([0, 100000], , ÿ1)]
(GF013_6) yuan
7. Health care expenses, including fitness training and product equipment, health care products, etc. [hc([0, ÿ), , ÿ1), sc([0, 100000], , ÿ1)] **(GF013_7)**
8. Beauty expenses, including cosmetics, beauty care, massage, etc. [hc([0, ÿ), , ÿ1), sc([0, 100000], , 1)] **(GF013_8)** yuan 9. Purchase, maintenance
and spare parts of automobiles [hc([0, ÿ), , ÿ1), sc([0, 100000], , ÿ1)] **(GF013_9)** yuan 10. Purchase, maintenance and spare parts of
various means of transportation and communication. Note: Transportation includes bicycles and electric bicycles but not cars.
Communication includes telephones and mobile phones [hc([0, ÿ), , ÿ1), sc([0, 100000], , 1)] **(GF013_10)** yuan 11. Property fees, including parking fees
[hc([0, ÿ), , ÿ1), sc([0, 100000], , ÿ1)] **(GF013_11)** yuan 12. Taxes and miscellaneous fees paid to relevant government
departments, excluding income tax [hc([0, ÿ), , ÿ1), sc([0, 10000 0], 13. Social donations, including cash, food, clothing, etc. [hc([0,
, ÿ1)] **(GF013_12)**
ÿ), , ÿ1), sc([0, 100000], ,)] **(GF013_13)** yuan 14. Rent of house or bed, including accommodation expenses for household ÿ1
members outside, such
as school dormitory fees, excluding hotel fees for travel [hc([0, ÿ), , ÿ1), sc([0, 100000], , ÿ1)] **(GF013_14)** yuan 15. Expenses for
setting up and hosting wedding banquets [hc([0, ÿ), , ÿ1), sc([0, 100000], , ÿ1)] **(GF013_15)**
yuan 16. Expenditure on epidemic prevention, including the purchase of masks, protective clothing and disinfectants [hc([0,
ÿ), , ÿ1), sc([0, 100000], , ÿ1)] **(GF013_16)** yuan [Interviewer's note: If there is no corresponding item, use "0" yuan to represent it. If you can't remember,
please ask the respondent to estimate a number. If the respondent refuses to answer or forgets, fill in "-1".]

GF014Since the outbreak of the epidemic, do you think [XMainR] 's family income can cover daily expenses?

1. Very difficult 2.
Somewhat difficult
3. Easy 4.
Very easy

GF015 Since the outbreak of the epidemic, has [XMainR] stopped paying rent, mortgage, and utility bills? (Multiple choices) 1.
Stopped paying rent
2. Stopped paying
mortgage 3. Stopped paying
utility bills 4. None of the above

[conflict(4, [4])]

Machine Translated by Google

GF016Since the income is not enough to maintain daily life, how did [XMainR]'s family get through the difficult times? The following amounts are the total amounts since the outbreak of the epidemic (multiple

choices are allowed). [Interviewer's note: If the respondent refuses to answer

or forgets, fill in "-1".] 1. Reduced spending, a total reduction of [hc((0, ÿ), , ÿ1), sc((0, 50000], , ÿ1)] **(GF016_1)** yuan 2. Used savings, withdrew [hc((0, ÿ), , ÿ1), sc((0, 50000], , ÿ1)] **(GF016_2)** yuan 3. Sold assets, such as **(GF016_9) ,** and obtained [hc((0, ÿ), , ÿ1), sc((0, 50000], ,

1)] **(GF016_3)** yuan

4. Get help from relatives and friends, that is, money that does not need to be repaid, and get [hc((0, ÿ), , ÿ1), sc((0, 500

00], , ÿ1)] **(GF016_4)**

5. Borrow money from relatives and friends, and get [hc((0, ÿ), , ÿ1), sc((0, 50000], , ÿ1)] **(GF016_5)** yuan . 6. Borrow money from the bank, and get [hc((0, ÿ), , ÿ1), sc((0, 50000], , ÿ1)] **(GF016_6)** yuan . 7. Others, such as **(GF016_8) ,** get [hc((0, ÿ), , ÿ1), sc((0, 50000], , ÿ1)] **(GF016_9) yuan.**

F016_7) 8. Do nothing

[conflict(8, [8])]

GF017 Did the respondents ask for help when filling in this part of the questionnaire?

1. Never 2. A few times 3. Most of the time

I. Housing conditions

I001 [XFinancialResp] What is the building structure of the house you are living in? [Interviewer's note: If the interviewer is at the respondent's home, the interviewer should record it himself. Otherwise, ask the respondent about the relevant situation.]

1. Reinforced concrete or brick-wood structure 2. Adobe house/ earth house 3. Wooden house/ thatched house 4. Cave house 5. Mongolian yurt/ felt house/tent 6. Stone house 7. Other structures, please specify **(I001_1)**

I002 [XFinancialResp] When did you move into the house you are currently living in? [hc([1900, 2020], , ÿ1)] years

[Interviewer's note: If the respondent refuses to answer or forgets, please fill in "-1".]

I003 If you are not sure of the exact year, please select from the following options:

1. 0-5 years 2. 5-10 years 3. 10-20 years 4. 20-30 years 5. 30-40 years 6. More than 40 years

I004 [XFinancialResp] Is the building where you are living a bungalow or a building?

[Interviewer's note: If the interviewer is at the respondent's home, the interviewer should record it himself. Otherwise, ask the respondent about the relevant situation.]

1. Bungalow

Machine Translated by Google

2. General buildings

3. Single-family house

Is **I005** an independent bungalow or a large courtyard?

[Interviewer's note: If the interviewer is at the respondent's home, the interviewer should record it himself. Otherwise, ask the respondent about the relevant situation.]

1. Independent bungalow

2. The slum

I006Which floor is this house on?

[Interviewer Note: If you live on the second floor underground, please fill in 2nd floor in option 2. If the interviewer is at the respondent's home, the interviewer should record it himself. Otherwise, ask the respondent Relevant information of the visitor.

1. Above ground [hc([1, ÿ), , ÿ), sc([1, 20], 2. Underground , ÿ)] **(I006_1)** layer

[hc([1, ÿ), , ÿ), sc([1, 5], , ÿ)] **(I006_2)** layer

Is there an elevator at **I007 ?**

[Interviewer's note: If the interviewer is at the respondent's home and knows the answer, the interviewer should record it himself. Otherwise, ask the respondent about the relevant situation.]

1. Yes

2. No

I008Is the elevator in the building already there when it was built or was it added later?

[Interviewer's note: If the respondent refuses to answer or forgets, please fill in "-1".]

1. Just built

2. Added later, the time added is [hc([1900, 2020], , ÿ1)] **(I008_1)**

3. Don't know

I009 Is the residence barrier-free? (e.g., ramp without steps, etc.)

[Interviewer's note: If the interviewer is at the respondent's home and knows the answer, the interviewer should record it himself. Otherwise, ask the respondent about the relevant situation.]

1. Yes

2. No

3. Flat ground with no steps, no barrier-free access required

I010How many steps do I have to climb to get to my front door when I come back from outside? If there is an elevator, how many steps do I have to climb to get to my front door when I take the elevator? ________

[hc([0, ÿ), , ÿ), sc((0, 30], [Interviewer note: If the , ÿ)]

interviewer is at the respondent's home and knows the answer, the interviewer should record it himself. Otherwise, ask the respondent about the relevant situation.]

The house **I011** is living in has [hc([0, ÿ), , ÿ), sc((0, 20], , ÿ)] **(I011_1)** room [hc([0, ÿ), , ÿ)] **(I011_2)** living room [hc([0, ÿ), , ÿ), , ÿ), sc((0, 20], ÿtoiletÿ [hc([0, sc((0, 20], , ÿ)] **(I011_3)** bathroom

ÿ), , ÿ), sc((0, 20], , ÿ)] **(I011_5)** balcony. , ÿ)] **(I011_4)** kitchen [hc([0, ÿ), , ÿ), sc(________

(0, 20],

[Interviewer note: If the interviewer is at the respondent's home and knows the answer, the interviewer should record it himself. Otherwise, ask the respondent about the relevant situation.]

I012 How far is the nearest toilet from the house where [XFinancialResp] is living? [hc([0, ÿ), , ÿ), sc((0, 100], , ÿ)] m

I013 What is the toilet like? Is it a squat toilet or a sit-down toilet? If there are both, choose the sit-down toilet.

[Interviewer's note: If the interviewer is at the respondent's home and knows the answer, the interviewer should record it himself. Otherwise, ask the respondent about the relevant situation.]

1. Squat

2. Sitting

Machine Translated by Google

I014Can the toilet be flushed?

[Interviewer's note: If the interviewer is at the respondent's home and knows the answer, the interviewer should record it himself. Otherwise, ask the respondent about the relevant situation.]

1. Yes 2.

No

I015 [XFinancialResp] Is there electricity in the house you are living in? [Interviewer's note: If the

interviewer is in the respondent's home and knows the answer, the interviewer should record it himself. Otherwise, ask the respondent about the relevant situation.]

1. Yes 2.

No

I016 Is there running water? There is water when you turn on the tap.

[Interviewer's note: If the interviewer is at the respondent's home and knows the answer, the interviewer should record it himself. Otherwise, ask the respondent about the relevant situation.]

1. Yes 2.

No

I017 Are there bathing facilities in the accommodation? What are they like?

1. Unified hot water

supply 2. Home-installed water

heater 3. None

I018 Is there pipeline gas or natural gas? 1. Yes 2. No

I019 Is there centralized heating? (Does not include local heating and air conditioning that can provide

heating)

1. Yes 2. No

I020 If you heat your home, what is the main energy source? 1. Solar energy 2. Coal,

briquettes 3.

Pipeline natural gas or coal

gas 4. Liquefied petroleum gas 5.

Electricity 6. Straw,

firewood

7. Others, please specify

(I020_1) 8. No heating ________

I021 If you cook, what is the main fuel you use? 1. Coal, briquettes 2. Pipeline

natural gas or coal gas 3.

Biogas 4. Liquefied petroleum gas 5.

Electricity 6.

Straw, firewood 7. Solar

energy 8.

Others, please specify

(I021_1) 9. No

cooking ________

Machine Translated by Google

I022 [XFinancialResp] Is there a telephone installed at your residence? [Interviewer's note: If the interviewer is at the respondent's home and knows the answer, the interviewer should record it himself. Otherwise, ask the respondent about the relevant situation.]

1. Yes
2. No

I023 [XFinancialResp] Is broadband internet available where I live?

[Interviewer's note: If the interviewer is at the respondent's home and knows the answer, the interviewer should record it himself. Otherwise, ask the respondent about the relevant situation.]

1. Yes
2. No

I024 [XFinancialResp] Is there an air purifier where you are living? [Interviewer's note: If the interviewer is at the respondent's home and knows the answer, the interviewer should record it. Otherwise, ask the respondent about the situation.]

1. Yes
2. No

I025How clean is the interior of this household?

ÿInterviewer's note: Interviewer should record by himself.ÿ

1. Very clean 2. Very clean
3. Clean 4. Average 5. Untidy 6. Not applicable

I026 What is the indoor temperature of this household?

[Interviewer's note: Interviewer should record it by himself.] 1. Very hot 2. Relatively hot 3. OK 4. Relatively cold 5. Very cold 6. Not applicable

I027What is the condition of this household's floor?

[Interviewer's note: If the interviewer is at the respondent's home and knows the answer, the interviewer should record it himself. Otherwise, ask the respondent about the relevant situation.]

1. Flat covered ground, such as carpet, wooden floor, marble, tile, or floor leather 2. Flat cement ground 3. Uneven ground

I028 [XFinancialResp] What is the current building area of housing? [hc((0, ÿ), ______ , ÿ), sc((10, 500], , ÿ)] m2

I029 Is the tap water treated by the water plant?

1. Yes
2. No

997. Don't know

Machine Translated by Google

G2 Personal income

G2. Proxy mode confirmation

proxy_8 Interviewer records: For the personal income module, is the proxy questionnaire model used?

1. Yes
2. No

GA. Personal income

[Quote: This part of the questionnaire was asked to the main respondent and his/her spouse separately. It is not allowed to ask someone else to fill in this part for you.]

GA001Did [XRName] receive any salary, including bonuses and various subsidies, excluding retirement, resignation or early retirement salary in the past year?

The salary here comes from all the hired work.

1. Yes
2. No

GA002 How much money did [XRName] receive in the past year? [hc((0, ÿ), , ÿ1), sc((0, 240000], , ÿ1), ub ([ÿ1], [5000, 10000, 30000, 50000, 100000])] yuan [Interviewer's note: If the respondent refuses to answer or forgets, fill in "-1".]

GA003 Are various insurances, income tax, housing provident fund or other miscellaneous expenses deducted from the salary mentioned above?

1. Yes
2. No 997.

Don't know 999.

Refuse to answer

GA004 [XRName] [XGA004Text] Individual income tax, various insurances, housing provident fund or other miscellaneous fees deducted or paid

How much is it in

total? [Interviewer's note: If the respondent refuses to answer or forgets, select the first option

1. _______ and fill in "-1".] [hc((0, ÿ), , ÿ1), sc((0, 10000], , ÿ1), ub([ÿ1], [300, 500, 1000, 2000, 3000])] **(GA004_1)** yuan/month

[hc((0, ÿ), , ÿ), sc((0,

2. _______ 100000], , ÿ)] **(GA004_2)** yuan/year 3. Or equivalent to salary [hc((0, 100], 4. No, 0

yuan _______ , ÿ)] **(GA004_3)** %

GA005 In the past year, did [XRName] receive any of the following transfer payments? Note that after the outbreak,

The epidemic subsidy is also a transfer payment income, so it must be filled in here (multiple

choices are allowed). [Interviewer Note: If the respondent refuses to answer or

forgets, fill in "-1".] 1. Retirement or pension, including government agency and public institution retirement pension, enterprise employee basic pension insurance, enterprise supplementary pension insurance, retirement or internal retirement compensation, rural or urban and rural or urban resident pension insurance, commercial pension insurance, life insurance, land acquisition pension insurance, etc., received [hc((0, ÿ), , ÿ1), sc((0, 100000], , ÿ1), ub([ÿ1], [1000, 3000, 6000, 10000, 25000])] **(GA005_1)** yuan

2. Unemployment benefits: [hc((0, ÿ), , ÿ1), sc((0, 50000], , ÿ1)] **(GA005_2)** yuan received 3. Pension cards or coupons: [hc((0, ÿ), , ÿ1), sc((0, 50000], , ÿ1)] **(GA005_3)** yuan received 4. Pension subsidies for the elderly: [hc((0, ÿ), , ÿ1), sc((0, 50000], , ÿ1)] **(GA005_4)** yuan received _______

Machine Translated by Google

5. Work injury insurance includes compensation for lost work, disability allowance, etc., and the amount of compensation received is [hc((0, ÿ), , ÿ1), sc((0, 50000], , ÿ1)] **(GA005_5)**

6. Old-age allowance for one-child, received [hc((0, ÿ), , ÿ1), sc((0, 50000], , ÿ1)] **(GA005_6)** yuan 7. Medical assistance, received [hc((0, ÿ), , ÿ1), sc((0, 100000], , ÿ1)] **(GA005_7)** yuan 8. Other government subsidies to individuals, excluding minimum living allowance, five guarantees, special hardship and poverty subsidies, please indicate **(GA005 _8_1) ,** received [hc((0, ÿ), , ÿ1), sc((0, 100000], , ÿ1)] **(GA005_8)** yuan 9. Other transfer payments from society to individuals, such as social donations, please indicate **(GA005_9_1) ,** received [hc((0, ÿ), , ÿ1), sc((0, 100000], , ÿ1)] **(GA005_9)**ÿ10. None of the above [conflict(10, [10])]

GA006 Consider that if there were no epidemic, [XRName] might have some salary income, but because of the epidemic, the situation may have changed. So, from the Spring Festival to now, has the epidemic increased or decreased [XRName]' s salary income,

or has it had no effect? [Interviewer's note: If the respondent refuses to answer or forgets, fill in "-1".] 1. Decreased, decreased [hc((0, ÿ), , ÿ1), sc((0, 50000], , ÿ1), ub([ÿ1], [1000, 3000, 5000 , 10000, 20000])] **(GA006_1)** yuan

2. Increase, increase [hc((0, ÿ), , ÿ1), sc((0, 50000], , ÿ1), ub([ÿ1], [1000, 3000, 5000 , 10000, 20000])] **(GA006_2)** yuan 3. No change

GA007 Has [XRName] made a will? 1. Yes 2. No

GA008 Has [XRName] considered the arrangement of his property after his death? 1. Yes 2. No 3. No property

GA009 [XGA009Text] If [XRName] dies, how will the property be distributed?

[Interviewer Note: If the respondent refuses to answer or forgets, fill in "-1".]

1. Spouse [hc((0, 100], , -1)] **(GA009_1)** % 2. Children, son-in-law, daughter-in-law

3. Brothers and sisters [hc((0, 100], , ÿ1)] **(GA009_3)** % 4. Other relatives [hc((0 , 100], , ÿ1)] **(GA009_4)** % 5. Parents, parents-in-law [hc((0, 100], , ÿ1)] **(GA009_5)** % 6. Grandchildren 7. Friends [hc((0, 100], , ÿ1)] (GA009_7) % 8. Charitable organizations [hc((0, 100], , ÿ1)] **(GA009_8)** % 9. Others, please specify **(GA009_9_1) ,** [hc((0, 100], , ÿ1)] **(GA009_9)** % 10. No allocation

997. [XProxyText]

[conflict(10, 997, [10, 997])]

To which children/spouse of children is the **GA010** property distributed? The spouse of the child is counted as the child portion.

In the options, please add the names of [XRName] 's children (multiple selections are allowed).

Machine Translated by Google

1-25. [XChildAliveName[ ]] 26-35. Other

children, named **(GA010_1[i])** ________

The percentage of property obtained by **GA011[ ]** [XGAChildList1[ ]] and his/her spouse is [hc((0, 100], [Interviewer's ________ , ÿ1)] %.

note: If the respondent refuses to answer or forgets, please fill in "-1".]

GA014 Whose children are the grandchildren? If the father of the grandchild to whom the property is distributed is not the mother of the grandchild,

In the Children section, please add the names of [XRName] 's children (multiple selections are allowed).

1-25. [XChildAliveName[ ]] 26-35. Other

children, named **(GA014_1[i])** ________

GA015[ ] [XGAChildList2[ ]] The percentage of children who received property is [hc((0, 100], [Interviewer Note: If the ________ , ÿ1)] %.

respondent refuses to answer or forgets, fill in "-1".]

GA016 When do you think [XRName]' s assets (inheritance) will actually be distributed? Before or after your death?

1. Allocate after [XRName] dies 2.

Allocate while [XRName] is alive 997.

[XProxyText] [conflict(997,

[997])]

GA017 Is the distribution of [XRName] 's property distributed in accordance with inheritance law?

1. According to the inheritance law, the inheritance is divided equally among those who are eligible for inheritance .

2. Make your own arrangements

GA018 When do you think the property (estate) will actually be distributed? Before, after, or after the death of yourself and your spouse ?

1. Distributed after both [XRName] and his/her spouse die

2. Distributed while both [XRName] and his/her spouse are

alive 3. Distributed when either [XRName] or his/her spouse dies

997. [XProxyText]

[conflict(997, [997])]

Auxiliary variable definition

XFLHHFinancialNameList connects the names of the main respondent and his/her spouse with "or"

```
if (!empty("XMainRS"))
        { add("XFLHHFinancialNameList", value("XMainR")+"or"+value("XMainRS")) }
else
    { add("XFLHHFinancialNameList", value("XMainR") )
}
```

XFinancialRespHousehold Finance Respondent

```
for (var i1 = 1; i1 < 26; i1++) { if
   (equal("GB001", i1)) {
^^I^^Iadd("XFinancialResp", value("XHHMemberName[i1]"))


^^I} } if (equal("GB001", 26)) {
^^Iadd("XFinancialResp", value("XMainR")) } if

(equal("GB001", 27)) {
^^Iadd("XFinancialResp", value("XMainRS")) }
```

Machine Translated by Google

XGB005Text GB005 Words used in the title

```
add("XGB005Text[i]", "") if
(equal("GB004[i]", "2") || equal("GB004[i]", "997")) {
^^Iadd("XGB005Text[i]", "should") }
```

XFLHHNameList household members' names are connected by commas, and the words in the title are

```
if (!empty("XMainRS"))
        { add("XFLHHNameList", value("XMainR")+","+value("XMainRS")) }
else
    { add("XFLHHNameList", value("XMainR") )
}

for (var i1 = 1; greater("XHHMemberNum", i1, true) ; i1++){
        add("XFLHHNameList", value("XFLHHNameList")+","+value("XHHMemberName[i1]"))
}
```

XHHOtherMemberName2 Name of household member other than child

```
for (var k = 1; k <= 10; k++) {
    add("XHHOtherMemberName2["+(k+25)+"]", value("XHHOtherMemberName[k]"))
}
```

XPoorHHNameList of names of poverty subsidies given by the government

```
add("XPoorHHName[1]", "Five-guarantee households")
add("XPoorHHName[2]", "Low-income households")
add("XPoorHHName[3]", "Poverty-stricken households registered on
file") add("XPoorHHName[4]", "Other types of poverty-stricken households you mentioned, i.e. "+value("GE001_1"))
```

XPoorLstYr In the past year, has there been a list of poverty subsidies provided by the government?

```
add("XPoorLstYr[i]", "0") if
(equal("GE002[i]", "1"))
    { add("XPoorLstYr[i]", "1")

} if (equal("GE002[i]", "2") && equal("GE015[i]", "2020"))
    { add("XPoorLstYr[i]", "1")
}
```

XFLHHNameListPoorList of household members who received various poverty subsidies in the past year

```
add("XFLHHNameListPoor[i]", "") for
(var i1 = 1; i1 < 26; i1++) {
  ^^Iif (selected("GE003[i]", i1)) {
^^I^^Iadd("XFLHHNameListPoor[i]", value("XHHMemberName[i1]")+","+value("XFLHHNameListPoor[i]"))
^^I} }


if (selected("GE003[i]", 26)) {
^^I^^Iadd("XFLHHNameListPoor[i]", value("XMainR")+","+value("XFLHHNameListPoor[i]"))^^I }


if (selected("GE003[i]", 27)) {
^^I^^Iadd("XFLHHNameListPoor[i]", value("XMainRS")+","+value("XFLHHNameListPoor[i]"))^^I }
```

XProxyText adds "Don't know" option to some questions in proxy mode

```
add("XProxyText", "") if
(equal("proxy_8", "1")) {
^^Iadd("XProxyText", "Unknown") }
```

XGA004Text GA004 Words used in the title

```
add("XGA004Text", "")
if (equal("GA003", "2") || equal("GA003", "997")) {
^^Iadd("XGA004Text", "should") }
```

Machine Translated by Google

XGA009Text GA009 Words used in the title

```
add("XGA009Text", "")
if (equal("GA007", "1")) {
^^Iadd("XGA009Text", "In the will") }
```

XGAChildList1 GA partial child list 1

```
for (var i1 = 1; i1 <=25 ; i1++)
    { add("XGAChildList1[i1]", value("XChildAliveName[i1]"))

} for (var i1 = 26; i1 <=35 ; i1++)
    { add("XGAChildList1[i1]", value("GA010_1[i1]"))
}
```

XGAChildList2 GA partial children list 2

```
for (var i1 = 1; i1 <=25 ; i1++)
    { add("XGAChildList2[i1]", value("XChildAliveName[i1]"))

} for (var i1 = 26; i1 <=35 ; i1++)
    { add("XGAChildList2[i1]", value("GA014_1[i1]"))
}
```

Machine Translated by Google

This page intentionally left blank

Machine Translated by Google

V Epidemic

Machine Translated by Google

V. Proxy Mode Confirmation

proxy_14 Interviewer record: For the epidemic module, do you use the proxy questionnaire mode? 1. Yes 2.

No

VA. Disease Awareness

[Quote: Next, I would like to know the impact of the epidemic on [XRName] .]

VA001 Do you know that the following practices can reduce the risk of contracting the new coronavirus? (Multiple choices are allowed)

[Interviewer's note: The "epidemic module" only involves information about respondents related to epidemic prevention and control. For questions about the impact of the epidemic on respondents' medical

care,

employment and income, please refer

to other modules.] 1. Wash hands

2. Use alcohol and disinfectants 3.

Avoid shaking hands

with others 4. Wear masks and gloves 5. Avoid

traveling 6. Avoid going to large gatherings 7. Keep distance when

talking to others face to face 8. Others, please briefly explain **(VA001_1)**

10. [XRName] knew about the epidemic, but didn't know any preventive measures (exclusive option) 11. [XRName] had never

heard of the disease before and didn't know about the epidemic (exclusive option) [conflict(10, 11, [10, 11])]

VA002 Where did [XRName] learn how to reduce the risk of infection? (Multiple choices are allowed, no need to read the options) 1. TV news 2.

Newspaper news 3.

Online media such as

mobile phone news and WeChat 4. Radio broadcasts 5.

Friends, relatives,

colleagues, leaders, community staff 6. Medical staff 7.

Loudspeaker

broadcasts 8. Promotional

posters 9. Others

10. There is no external channel, [XRName] relies entirely on his own understanding and life experience (exclusive option)

[conflict(10, [10])]

VA003During the epidemic, does [XRName] wear a mask when going out? (Read the first three options)

1. Wear it every time I go out

2. Sometimes I don't wear it when

I go out 3. Never wear

it 4. Haven't gone out during the epidemic

VA004 Will [XRName] go out now? Will he wear a mask when he goes out? (optional)

1. I wear it when I go out now

Machine Translated by Google

2. I don't wear it when I go out now

3. I never go out

VA005 Do you wear a mask when you go out now ? If you used to wear one but don't now, when did you stop wearing one? (Select

[Interviewer

Note : Now sometimes I wear a mask and sometimes I don't. Please select the first option. If the respondent cannot answer the month, please

fill in "-1".] 1. I still wear one

when I go out 2. I wore one during the epidemic, but I don't wear one when I go out now: I have not worn a mask for [hc([0, 14], , ÿ1), sc([0, 6], ,

ÿ1)] **(VA005_1)** months 3. Did not

wear one during the epidemic and now 4. Did

not go out after the epidemic

VA006 was in Wuhan for three days after the city was locked down due to the epidemic, from New Year's Eve to the second day of the first lunar month (January 24 to January 26).

For the following items, did [XRName] buy more than usual due to the epidemic and stock up first? (Multiple choices are allowed)

1. Cereals, oils and

vegetables 2. Masks, hand sanitizer or

disinfectant 3. None of the above

[conflict(3, [3])]

VA007 During the epidemic, our government has taken some measures to control the new coronavirus. Do you think they are too strict, appropriate, or not strict enough?

(optional) 1. Too strict 2. Appropriate 3.

Not strict enough 997.

Don't know 999.

Refuse to answer

VB. Personal Illness and Isolation

VB001 [XRName] , have you, anyone around you, or anyone you know been diagnosed with COVID-19 or suspected of COVID-19?

Yes, what is the relationship with [XRName] ? (Multiple selections are allowed, no need to read the options)

1. Myself 2.

People living together: **(VB001_1)** 3. Other relatives (not living

together): **(VB001_2)** 4. Acquaintances and friends (such as good friends,

neighbors, colleagues, etc.): **(VB001_3)** 5. None 999. Refuse to answer

[conflict(5, 999, [5, 999])]

VB002 Are they all recovering well? Has anyone died? If so, what is their relationship with [XRName] ? (Multiple choices are allowed, no need to read the options) 1. People

who live together: **(VB002_1)** 2. Other relatives (not living

together): **(VB002_2)** 3. Acquaintances and friends (such as close friends,

neighbors, colleagues, etc.): **(VB002_3)** 4. None 999. Refuse to answer

[conflict(4, 999, [4, 999])]

Machine Translated by Google

VB004 Was [XRName] hospitalized for treatment of COVID-19 ? Did he stay in the hospital for a total of [hc([0, 250], , ÿ1)] days? (No

If hospitalized, fill in

"0") [Interviewer's note: If the respondent cannot answer, please fill in "-1".]

VB005 [XVAHospitalizationExcluded] Has [XRName] ever been isolated or placed under medical observation for the following reasons? Home quarantine

Leaving and closing the building are both considered as being isolated. (Multiple choices are allowed)

1. Traveling or going on a business trip (including going to work after

the Spring Festival) 2. Being a close contact of a

COVID-19 case 3. The residence or residential building is closed (excluding the

closure of the community) 4. Being required to be isolated when going to a medical

institution for diagnosis and treatment or after

being discharged from the hospital 5. The nucleic acid test

showed a positive result

6. No isolation experience

(exclusive option) 997. Don't know 999. Refuse to answer [conflict(6, 997, 999, [6, 997, 999])]

VB008 [XRName] was isolated for a total of [hc([1, 250], , ÿ1), sc([1, 14], [Interviewer Note: If there are multiple , ÿ1)] day?

isolation experiences, please inform the total duration. If the respondent cannot answer, please fill in "-1".]

VB009 Where is [XRName] isolated? (Multiple choices) 1. Hospital 2. Hotel or

other

centralized medical observation point 3. Own

residence 4. Other:

997. Don't ________ **(VB009_1)**

know 999. Refuse to

answer [conflict(997, 999,

[997, 999])]

VB010 How much did [XRName] pay for quarantine? Including food and lodging, the total is [hc([0, 100000], , ÿ1)] yuan. [Interviewer's note: If the respondent

cannot answer, please fill in "-1".]

VB011During [XRName] 's quarantine experience, have there been other people who were quarantined with [XRName] in the same quarantine residence, and could they see each other?

Face-to-face

conversation? 1. Yes

2. No 997. Don't know 999. Refuse to answer

VB012 Has [XRName] been tested for COVID-19?

[Interviewer's note: If the respondent cannot answer the month, please fill in

"-1".] 1. Yes, the most recent test was in [hc([2020,XIWYear], , ÿ1)] **(VB012_1)** ________ [hc

([1, 12], 2. , ÿ1), sc([1,XIWMonth], , ÿ1)] **(VB012_2)** Month

No 997.

Don't know 999.

Refuse to answer

Machine Translated by Google

VC. Personal activities during the epidemic

During the **VC000** epidemic [XVCNotInQuarantine] , did [XRName] ever stay at home for several days because of fear of infection , -1)] days (no such situation or leave home every day,

door? How many days was the longest time this happened? [hc([0, 250], fill in "0") [Interviewer's note: If the

respondent

cannot answer, please fill in "-1".]

[Quote: Next, I would like to know about some of [XRName]'s activities during the most severe period of the epidemic, which was the period after the Spring Festival this year [XVCOutbreak] , from January 25 to February 22.]

VC001 [XVCOutbreak] , where does [XRName] usually live?

[Interviewer's note: If the respondent does not know the street and community, he/she can choose as appropriate]

1. The same village/community as the current residence: [XRResidenceFull] 2. Other villages/

communities in the same district/county as the current residence : [XRResidenceCounty] : **(VC001_1)** Township/town/street/village/community

district

3. Current residence outside the county: **(VC001_2)** Province/City/District/County ____ **(VC001_3)** Township/town/street/village/community

district

4. Regions where none of the above options apply (Hong Kong, Macau, Taiwan and overseas)

999. Refuse to answer

VC002 [XVCOutbreak] [XVCNotInQuarantine] , the number of times [XRName] goes out every day is the same as if the epidemic had not occurred.

Compared with the situation, has it increased, decreased, or remained the same?

[Interviewer's note: If the epidemic did not happen, it cannot be interpreted as "last year's first lunar month" or "now when it is normal". Special case: When the interviewee knows nothing about the epidemic and the interviewer cannot describe the situation if the epidemic did not happen, the interviewer can interpret "compared with the situation if the epidemic did not happen" as "compared with last year's first lunar month, without considering the factor of changes in physical condition". The same below.]

1. Increased significantly

2. Increased slightly 3.

No change 4. Decreased

slightly 5. Decreased

significantly

VC003 [XVCOutbreak] [XVCNotInQuarantine] , [XRName] how much time do you spend outside every day and what would you do if the epidemic had not occurred?

Compared with the previous situation, has it increased, decreased, or remained the same?

[Interviewer's note: This situation did not occur during the epidemic, so it cannot be interpreted as "last year's first month" or "now is a normal time".]

1. Increased significantly

2. Increased slightly 3.

No change 4. Decreased

slightly 5. Decreased

significantly

VC004 [XVCOutbreak] [XVCNotInQuarantine] , compared with the situation if the epidemic had not occurred, has the time spent on vigorous activities every day increased, decreased, or remained the same? Vigorous activities are very physically demanding and can make people breathe rapidly, including carrying heavy objects, digging, farming, aerobic exercise, fast cycling, cycling with cargo, etc., and also include vigorous indoor activities. [Interviewer's note: The epidemic has not occurred, so it cannot be interpreted as "last year's first month" or "now normal time".]

1. Significant increase 2.

Slight increase

Machine Translated by Google

3. No change 4. Slight decrease 5. Significant decrease

VC005 [XVCOutbreak] [XVCNotInQuarantine] , compared with if the epidemic had not occurred, has the time spent doing moderate-intensity physical activities increased, decreased, or remained the same every day? Moderate-intensity physical activities include carrying light objects, mopping the floor, riding a bicycle at a normal speed, brisk walking, etc. [Interviewer's note: If the epidemic had not occurred, it cannot be interpreted as "last year's first month" or "now when it is normal."]

1. Increased significantly
2. Increased slightly 3. No change 4. Decreased slightly 5. Decreased significantly

VC006 [XVCOutbreak] [XVCNotInQuarantine] , [XRName] Perform light physical activity every day, such as walking, strolling, etc.

Compared with the period if the epidemic had not occurred, has the activity time increased, decreased, or remained the same? [Interviewer's note: If the epidemic had not occurred, it cannot be interpreted as "last year's first month" or "normal time now".]

1. Increased significantly
2. Increased slightly 3. No change 4. Decreased slightly 5. Decreased significantly

VC007 [XVCOutbreak] [XVCNotInQuarantine] , the frequency of [XRName] visiting and what would happen if the epidemic had not occurred

Compared to the previous year, has it increased, decreased, or remained the same? [Interviewer's note: This situation did not occur during the epidemic, and cannot be interpreted as "last year's first month" or "now is a normal time."]

1. Significant increase
2. Slight increase 3. No change 4. Slight decrease
5. Significant decrease
6. Never visit

VC008 [XVCOutbreak] [XVCNotInQuarantine] , Compared with the situation if the epidemic had not occurred, has the frequency of playing mahjong, chess and cards increased, decreased, or remained the same? [Interviewer's note: The situation if the epidemic had not occurred cannot be interpreted as "last year's first month" or "now is normal."]

1. Significant increase
2. Slight increase 3. No change 4. Slight decrease
5. Significant decrease
6. Never participate in chess and card activities

VC009 [XVCOutbreak] [XVCNotInQuarantine] , [XRName] How often do you dance in the square and if the epidemic had not happened?

Compared with the previous situation, has it increased, decreased, or remained the same? [Interviewer's note: This situation did not occur during the epidemic, and cannot be interpreted as "last year's first month" or "now is a normal time".]

1. Significant increase

Machine Translated by Google

2. Slightly increased 3.

No change 4. Slightly

decreased 5.

Significantly decreased

6. Never dance square dance

VC010 [XVCOutbreak] , [XRName]' s frequency of phone calls and text messages is compared to what it would have been if the epidemic had not occurred.

Has it increased, decreased, or remained the same?

[Interviewer's note: This situation did not occur during the epidemic, so it cannot be interpreted as "last year's first month" or "now is a normal time."]

1. Increased significantly

2. Increased slightly 3.

No change 4. Decreased

slightly 5. Decreased

significantly 6. The

accommodation is not suitable 7.

Never

VC011 [XVCOutbreak] , [XRName], has the frequency of using the Internet to contact relatives and friends, such as sending WeChat messages, voice and video chats, increased,

decreased, or remained the same compared to the situation if the epidemic had not occurred? [Interviewer's note: If the

epidemic had not occurred, it cannot be interpreted as "last year's first month" or "now is normal."]

1. Increased significantly

2. Increased slightly 3.

No change 4. Decreased

slightly 5. Decreased

significantly 6. The

accommodation is not suitable 7.

Never

VC012 [XVCOutbreak] , has [XRName] ever felt scared because of the epidemic or anything related to it?

[Interviewer's note: This situation did not occur during the epidemic, so it cannot be interpreted as "last year's first month" or "now when it is

normal."] 1. Rarely or not at all 2. Not

very often 3.

Sometimes or half of the time 4. Most of the time 997.

Don't know 999. Refuse to

answer

VC013 [XVCOutbreak] , has [XRName] ever felt nervous or anxious because of the epidemic or things related to the epidemic? [Interviewer's note: The epidemic did

not happen in this way, so it cannot be interpreted as "last year's first month" or "now when it is normal."] 1. Rarely or not at all 2. Not very

often 3. Sometimes or half of the time 4.

Most of the time

997. Don't know 999. Refuse to answer

VC014 [XVCOutbreak] , [XRName] 's smoking volume has increased, decreased, compared to what it would have been if the epidemic had not occurred.

Machine Translated by Google

Has it changed, or has it not

changed? [Interviewer's note: "Never smoked" includes never smoking in recent years. This situation did not occur during the epidemic, and cannot be interpreted as "last year's first month" or "now

when it is normal."] 1.

Significant increase 2.

Slight increase 3. No

change 4. Slight

decrease 5. Significant

decrease 6. Never smoked

VC015 [XVCOutbreak] , has [XRName]'s alcohol consumption increased, decreased, or remained the same compared to if the epidemic had not occurred? [Interviewer's note: "Never drank" includes never drinking

in recent years. If the epidemic had not occurred, it cannot be interpreted as "last year's first month" or "now when it is normal."] 1. Significantly increased 2. Slightly increased

3. No change 4. Slightly

decreased 5.

Significantly decreased

6. Never drank

VC016 [XVCOutbreak] , [XRName] Compared with the situation if the epidemic had not occurred, has the sleep time every night increased, decreased, or remained the same? Sleep time refers to the time actually asleep, which may be shorter than the time lying in bed. [Interviewer's note: The epidemic had not occurred, so it cannot be interpreted as "last year's first month" or "now normal time".]

1. Increased significantly

2. Increased slightly 3.

No change 4. Decreased

slightly 5. Decreased

significantly

VC017 [XVCOutbreak] , [XRName]' s daily food intake has increased compared to what it would have been if the epidemic had not occurred.

Less, or no change? [Interviewer's

note: This situation did not occur during the epidemic, and cannot be interpreted as "last year's first month" or "now is a normal time".]

1. Increased significantly

2. Increased slightly 3.

No change 4. Decreased

slightly 5. Decreased

significantly

VD. Control of residence during epidemic period

VD001 I would like to know from you that due to the epidemic control, has the community or village where [XRName] lived since the Spring Festival implemented the following types of restrictions on the entry and exit of internal and external personnel? How many days has [XRName] experienced each restriction? The order of inquiry for these four types of restrictions is arranged from tight to loose, and the restriction periods cannot overlap, and the total length of time should not exceed [XIWMonth] months. (Multiple selections are allowed, and prompts are given item by item)

Machine Translated by Google

[Interviewer's note: Only restrictions experienced by the interviewee are considered. Due to relocation or repeated outbreaks, the experience of restrictions may be discontinuous, and the discontinuous stages of the same period need to be added together. Passes and facial recognition, which are means of identifying people in the area, are restrictions on visitors, not restrictions on entry and exit of people in the area. If a restriction experience has not yet ended, the duration ends on the day of the interview. Interviewers can ask for the approximate start and end dates to assist the interviewee in calculation. If the interviewee cannot answer the number of days experienced, fill in "-1".]

1. Closed management of internal personnel, no outsiders allowed to enter: Unless there are special circumstances, people in the area are completely prohibited from entering

The total experience [hc([1, 120], , ÿ1)] **(VD001_1)** days 2. Semi-closed management for internal personnel, no entry for outsiders: People in the area can enter and exit, but the number of entries and exits per day is restricted.

For example, each household is limited to the number of times it is allowed to go out to purchase daily necessities, etc., with a total experience of [hc([1, 2 , 00], ÿ1)] **(VD001_2)** days

3. Internal personnel can enter and exit without restrictions, and outsiders are prohibited from entering: Visitors cannot enter the community/village, and the total experience is [hc([1, 300], , ÿ1)] **(VD001_3)** days 4.

 Internal personnel can enter and exit without restrictions, and outsiders can enter under certain conditions: Visitors can enter the community with a health code

District/village, total experience [hc([1, 400], , ÿ1), sc([1, 300], 5. None of the above types of control have , ÿ1)] **(VD001_4)** days been experienced, and the freedom of entry and exit of internal and external personnel is the same as before the epidemic (exclusive option)

997. I have no idea whether the above four control measures have ever been implemented .

999. I refuse to answer

[conflict(5, 997, 999, [5, 997, 999])]

VD002 I would like to know from you, since the Spring Festival, have the square dance activities in the community or village where [XRName] lives been cancelled? Counting all the square dance cancellations that [XRName] has experienced, there are a total of [hc([0, 250], , ÿ1)] days to date ? (If not experienced, fill in "0") [Interviewer Note: If the respondent cannot answer, please fill in "-1".]

VD003 I would like to know from you, since the Spring Festival, have the mahjong halls or community activity rooms and other chess and card entertainment venues in the community or village where [XRName] lives been cancelled? Counting all the public chess and card activities that [XRName] has experienced cancellation, as of today, there are a total of [hc([0, 250], , ÿ1)] days? (If not experienced, fill in "0") [Interviewer Note: If the respondent cannot answer, please fill in "-1".]

Auxiliary variable definition

Has **XQuarantined** been quarantined? **if**

```
(selected("VB005", "1") || selected("VB005", " 2") || selected("VB005", "3") || selected("VB005", "4") ÿ || selected("VB005", "5"))
 { add("XQuarantined", "1") } else
     { add("XQuarantined", "0")

}
```

XVCOutbreak wording: Epidemic activity observation period

```
add("XVCOutbreak", "First month")
```

XVCNotInQuarantine wording: Exclude quarantine during the epidemic

```
if (equal("XQuarantined", "1"))
     { add("XVCNotInQuarantine", ", does not include mandatory quarantine")
}
```

XVAHospitalizationExcluded wording: Isolation exclusion hospitalization time

```
if (greater("VB004", "0")) {
     add("XVAHospitalizationExcluded", "Exclude those days of hospitalization,")
}
```

Machine Translated by Google

EX Exit Questionnaire

Machine Translated by Google

EXB. Basic Information

[Quote: Sorry, the previous information was a bit incomplete, I want to confirm it again.]

EXB001 What is the date of death of [XRName] ? [hc([2011, 2020], , ÿ), sc([ZIWYear, 2020], , ÿ)] **(EXB00 1_1)** year [hc([1, 12], , ÿ)]

(EXB001_3) day [Note to interviewers: Use 4 digits to represent the year] , ÿ)] **(EXB001_2)**ÿ[hc([1, 31],

EXB002 Is [XRName] 's date of death the Gregorian calendar (solar calendar) or the lunar calendar (lunar calendar)? 1.

Gregorian calendar (solar

calendar) 2. Lunar calendar (lunar calendar)

EXB003 Where did [XRName] live before his death?

1. Mainland China **(EXB003_1)** Province/City/County/District ________ **(EXB003_2)** Township/town/street/village/community ________

(EXB003_3) Community/Building/Unit/House

Number 2. Hong

Kong, China 3.

Macau, China 4.

Taiwan, China 5. Abroad ________ **(EXB003_4)**

EXB004 What was the type of residence of [XRName] before his death? Was it a family home, workplace, nursing home or other elderly care institution, hospital ward, or

other? 1. Family

home 2. Workplace

3. Nursing home or other elderly care

institution 4.

Hospital ward 5. Other, please specify ________ **(EXB004_1)**

EXB005 Where did [XRName] die?

1. [XAliveResidenceFull] 2. Mainland

China: **(EXB005_1)** Province/City/County **(EXB005_3)** ________ **(EXB005_2)** Township/town/street/village/community ________

Residential District/Building Number/Unit/House

Number 3. Hong

Kong, China 4.

Macau, China 5.

Taiwan, China 6. Overseas ________ **(EXB005_4)**

EXB006 When [XRName] died, was he/she at home, at work, in a hospital, in a nursing home, in a relief facility, or elsewhere?

Place?

1. Home 2.

Workplace 3.

Hospital 4.

On the way to the

hospital 5.

Nursing home 6.

Relief agency 7. Other places, please specify ________ **(EXB006_1)**

EXB007Did you ever think that [XRName] would die at that time?

Machine Translated by Google

1. Expected 2.

Unexpected 3. Others,

please specify **(EXB007_1)** ________

EXB008 How long did [XRName] last from his last illness until his death? One or two hours, less than a day, less than a week, less than a month.

Months, less than a year, or several years?

1. One or two hours or no signs 2. Less than a day

3. Less than a week

4. Less than a month

5. Less than a year 6.

One year and more,

is [hc([1,XRExitAge], ________ , ÿ), sc([1, 20], , ÿ)] **(EXB008_1)** year

EXB009 What was [XRName] 's marital status at the time of

his death? 1.

Married 2. Separated (no longer living together as a

couple) 3.

Divorced 4.

Widowed 5. Never

married 6. Cohabiting

EXB010 Was [XRName] living with his spouse/communicative partner at the time of his death?

1. Yes 2.

No

EXB011 Does [XRName] have a death certificate? 1.

Yes 2. No

EXB012 Where do I apply for the death certificate of [XRName] ?

[Interviewer's note: Please try to find the medical certificate of death. The certificate may have relevant application

address information.] 1. The village/community where the previous residence

[XAliveResidenceFull] is located 2. Other villages/communities in the county/city/district where the previous residence [XAliveResidenceFull] is

located **(EXB012_1)** 3. Others: **(EXB012_2)** Province/city/ ________ **(EXB012_3)** Township/town/street/village/community

county 4. Overseas: **(EXB012_4)**

EXB013 Has [XRName] 's account been cancelled? 1. Yes 2. No

Why is **EXB014** not cancelled? 1. The time is

too short and I didn't have time to process it. 2.

Cancellation is not important. 3. Others,

please specify **(EXB014_1)** ________

VA1A730 Could you please provide me with [XRName] 's ID number before his death ? ________

[Interviewer's note: Please try to find the medical certificate of death, which will contain the relevant ID number. If there is no death certificate, please ask the agent if they can provide other documents including [XRName] 's ID number before his death or ask the agent to recall the ID number.

Machine Translated by Google

This ID number information will be mainly used to match personal records in the National Death Registry System and will only be used for scientific research. No personal privacy information will be disclosed. If the agent cannot answer, fill in "-1". ÿ

VA1A750 Please record the cause of death reported on the medical certificate of death ________

After getting permission, please take a photo of the medical certificate of death. **(VA1A750_1)** [Interviewer's note: Please ask the agent/family member to try to find the medical certificate of death and record the cause of death reported on it. Explain the data confidentiality regulations to the agent/family member. This part of the information will only be used for scientific research and will not disclose any personal privacy information. After getting permission, please take a photo of the medical certificate of death.]

EXC. Family

[Quote: Now I would like to ask some questions about [XRName] 's children]

EXC001[ ] Is [ZChildName[ ]] (gender: [XEChildGenderDis[ ]]) still alive?

1. Yes
2. No

EXC003[ ] [XEChildAliveName[ ]] When was he born? [hc([1910, 2018], , ÿ), sc([1940, 2018], , ÿ)] year

[Interviewer's note: Use 4 digits to represent the year. If the respondent cannot remember the year of birth, the year of birth can be estimated by the current age of the children, or the year of death, the age of the respondent when the children were born, etc.]

EXC004[ ] [XEChildAliveName[ ]]'s gender? 1. Male 2. Female

EXC005[ ] Excluding adult education, what is [XEChildAliveName[ ]]'s highest level of education?

1. No formal education 2. Did not finish primary school 3. Private school 4. Primary school graduate 5. Junior high school graduate 6. Senior high school graduate 7. Technical secondary school graduate (including secondary normal school, vocational high school) 8. Junior college graduate 9. Bachelor degree graduate 10. Master degree graduate 11. Doctorate degree graduate

997. Don't know999. Refuse to answer

EXC006[ ] One year before his death, how long did [XEChildAliveName[ ]] live with [XRName] ? [hc([0, 12] , , ÿ)] month ________

[Interviewer's note: Short visits to relatives do not count as living together; if you do not live together, please fill in 0; if you always live together, please fill in 12]

EXC007In the year before [ ] died, when [XRName] and [XEChildAliveName[ ]] were not living together, how often did [XRName] see [XEChildAliveName[ ]]?

Machine Translated by Google

1. Almost every day 2.

2-3 times a week 3.

Once a week 4.

Once every half month 5.

Once a month 6.

Once every three months

7. Once every six

months 8. Once a

year 9. Almost never 10.

Other

EXC008 How many grandchildren did [XRName] have before he died? (Including biological children, stepchildren, and adopted children, including grandchildren

Grandchildren) ________ of the family) , ÿ)]

EXC009 How many great-grandchildren (including great-grandchildren) did [XRName] have before he died? [hc([0, 25], ________ , ÿ)]

EXD. Health Status and Function (I)

EXDA001[ ] Has a doctor diagnosed [XRName] with [XEChroDisType[ ]] since [ZIWTime] ?

1. Yes 2.

No

EXDA002[ ] When was the first time that the doctor diagnosed [XRName] with [XEChroDisType[ ]]? **[Interviewer's note: Use** 4

digits to record the year; if you don't know,

please fill in -1]

1. Year [hc([1900, 2020], , ÿ1), sc([1920, 2020), , ÿ1)] **(EXDA002_1[i])** years 2. Age [hc([0, 120], , ÿ1), sc((0, 100], , ÿ1)]

(EXDA002_2[i]) years ________

EXDA003Did [XRName] have a heart attack or myocardial infarction between [ZIWTime] and the time of his/ her death ?

1. Yes 2.

No

EXDA004 When was the last time [XRName] had a heart attack? **[Interviewer note:**

Use 4 digits to record the year; if you don't

know, please fill in -1]

1. Year [hc([1900, 2020], , ÿ1), sc([1920, 2020), , ÿ1)] **(EXDA004_1)** years 2. Age [hc([0, 120], , ÿ1), sc((0, 100], , ÿ1)]

(EXDA004_2) years ________

EXDA005 Which organ or part of the body does [XRName] have or have had cancer? Include primary and metastatic tumors. (Multiple

(Optional) [Interviewer's note: If the respondent has recovered from cancer, it is

still necessary

to record] 1.

Brain 2.

Oral cavity

3. Larynx 4.

Pharynx 5. Thyroid 6. Lung

Machine Translated by Google

7. Breast

8.

Esophagus

9. Stomach

10. Liver 11.

Pancreas 12.

Kidney 13.

Prostate 14.

Testis 15.

Ovaries 16. Cervix

17. Endometrium 18.

Colon or

Rectum 19.

Bladder 20. Skin 21. Non-Hodgkin's Lymphoma 22.

Leukemia 23.

Other Organs **(EXDA005_1)**

EXDA006 In the two years before his death, did [XRName] use any of the following methods to treat the tumor or relieve the pain, nausea and other symptoms caused by the tumor?

(Multiple choices are allowed) [Interviewer

Note: Please read out the options one by one and ask the respondents to answer

them one by one. (1) "Chemotherapy" refers to the method of using chemical synthetic drugs to treat diseases. Chemotherapy (abbreviated as chemotherapy) is currently

one of the main means of treating tumors

and certain autoimmune diseases. (2) "Surgery" is the earliest method used to treat cancer and is currently the preferred treatment for many

early cancers. (3) "Radiotherapy" is a treatment method that uses various energies of radiation to irradiate tumors to inhibit and kill cancer cells.]

1. Taking Chinese

medicine 2.

Taking Western

medicine 3.

Chemotherapy 4.

Surgery 5. Radiotherapy 6. Other treatments, please specify

(EXDA006_1) 7. None of the above

[conflict(7, [7])]

EXDA007 Has any doctor diagnosed [XRName] with a recurrence of stroke since [ZIWTime] ?

1. Yes

2. No

EXDA008 When was [XRName] 's most recent stroke diagnosed?

[Interviewer's note: Use 4 digits to

record the year; if

you don't know, please fill in -1] 1. Year , ÿ1), sc([1920, 2020), , ÿ1)] **(EXDA008_1)** year

[hc([1900, 2020], 2. Age [hc([0, 120], , ÿ1), sc((0, 100], , ÿ1)] **(EXDA008_2)** years old

EXDA009 Did [XRName] have memory problems one month before his

death?

1. Yes 2. No

Machine Translated by Google

EXDA010 At what age did [XRName] start to have obvious memory problems? **[Interviewer's**

note: Use 4 digits to record the year; if you

don't know, please fill in -1]

1. Year [hc([1900, 2020], , ÿ1), sc([1920, 2020), , ÿ1)] **(EXDA010_1)** years 2. Age [hc([0, 120], , ÿ1), sc((0, 100], , ÿ1)] **(EXDA010_2)** years

EXDA011Do memory problems occur suddenly or over a slow process? 1.

Sudden 2.

Slowly

EXDA012 Are memory problems getting worse? 1.

Yes 2.

No

EXDA013Since [ZIWTime] , has [XRName] fallen down ? 1. Yes 2. No

EXDA014 How many times did [XRName] fall and get injured badly enough to need medical treatment? [hc([0, 99], , ÿ)] times

EXDA015Since [ZIWTime] , has [XRName] ever had a hip fracture? 1. Yes 2. No

EXDA016 Does [XRName] often suffer from body pain? Not at all, a little, some, quite a lot, or very much?

A

lot? 1. Not at all 2.

A little 3. Some

4. Quite a lot

5. Very much

EXDA017Did [XRName] have any other serious illnesses that I have not asked about since [ZIWTime] until his death ?

1. Yes

2. No

EXDA018What are these diseases? ________

EXDA019 Did [XRName] feel severely tired for at least one month in the year before his death?

1. Yes

2. No

EXDA020 Did [XRName] have incontinence for at least one month in the year before his death?

1. Yes

2. No

Machine Translated by Google

EXD. Health Status and Function (II)

EXDB001 Could you please tell me if [XRName] had difficulty dressing himself in the three months before his death due to health and memory problems, including Take clothes out of the closet, put them on, button them up, and tie their belts?

1. No Difficulty
2. It is difficult but still achievable
3. Having difficulties and needing help
4. Unable to complete

EXDB002Does anyone help you get dressed?

1. Yes
2. No

EXDB003 How long do you need help getting dressed?

1. _______ [hc((0, 99], , , ÿ)] **(EXDB003_1)** month
2. _______ [hc((0, 99]ÿ)] **(EXDB003_2)**
3. Since _______ [hc([1, 120], , ÿ), sc((1, 100], , ÿ)] **(EXDB003_3)** years old
4. Since _______ [hc([1900, 2020], , ÿ), sc([1920, 2020], , ÿ)] **(EXDB003_4)** years

EXDB004 Could you please tell me if [XRName] had difficulty bathing himself in the three months before his death due to health and memory issues?

1. No Difficulty
2. It is difficult but still achievable
3. Having difficulties and needing help
4. Unable to complete

EXDB005 Is there anyone to help you when you take a bath?

1. Yes
2. No

EXDB006 How long do you need help taking a bath?

1. _______ [hc((0, 99], , ÿ)] **(EXDB006_1)** month
2. _______ [hc((0, 99], , ÿ)] **(EXDB006_2)**
3. Since _______ [hc([1, 120], , ÿ), sc((1, 100], , ÿ)] **(EXDB006_3)** years old
4. Since _______ [hc([1900, 2020], , ÿ), sc([1920, 2020], , ÿ)] **(EXDB006_4)** years

EXDB007 Did [XRName] have difficulty feeding himself in the three months before his death due to health and memory issues?

1. No Difficulty
2. It is difficult but still achievable
3. Having difficulties and needing help
4. Unable to complete

EXDB008 Is there anyone to help you when you eat?

1. Yes
2. No

EXDB009 How long do you need help with eating?

1. _______ [hc((0, 99], , ÿ)] **(EXDB009_1)** month
2. _______ [hc((0, 99], , ÿ)] **(EXDB009_2)**

Machine Translated by Google

3.Since _______ [hc([1, 120], , ÿ), sc((1, 100], , ÿ)] **(EXDB009_3)** years old

4.Since _______ [hc([1900, 2020], , ÿ), sc([1920, 2020], , ÿ)] **(EXDB009_4)** years

EXDB010 Did [XRName] have difficulty getting out of bed or getting out of bed three months before his death due to health or memory issues?

1. No Difficulty
2. It is difficult but still achievable
3. Having difficulties and needing help
4. Unable to complete

EXDB011Is there anyone to help you when you get up or get out of bed?

1. Yes
2. No

EXDB012How long do you need help getting up and out of bed?

1. _______ [hc((0, 99], , , ÿ)] **(EXDB012_1)** month
2. _______ [hc((0, 99], , ÿ)] **(EXDB012_2)**

3.Since _______ [hc([1, 120], , ÿ), sc((1, 100], ÿ)] **(EXDB012_3)** years old

4.Since _______ [hc([1900, 2020], , ÿ), sc([1920, 2020], , ÿ)] **(EXDB012_4)** years

EXDB013 Did [XRName] have difficulty going to the toilet on his own in the three months before his death due to health and memory issues?

1. No Difficulty
2. It is difficult but still achievable
3. Having difficulties and needing help
4. Unable to complete

EXDB014Is there anyone to help you when you go to the toilet?

1. Yes
2. No

EXDB015 How long do you need help going to the toilet?

1. _______ [hc((0, 99], , , ÿ)] **(EXDB015_1)** month
2. _______ [hc((0, 99], , ÿ)] **(EXDB015_2)**

3.Since _______ [hc([1, 120], , ÿ), sc((1, 100], ÿ)] **(EXDB015_3)** years old

4.Since _______ [hc([1900, 2020], , ÿ), sc([1920, 2020], , ÿ)] **(EXDB015_4)** years

EXDB016 Did [XRName] have difficulty cooking for himself in the three months before his death due to health and memory problems? (Definition:

We define a meal as preparing the ingredients, cooking the food, and serving it on the table.

1. No Difficulty
2. It is difficult but still achievable
3. Having difficulties and needing help
4. Unable to complete

EXDB017 Is there anyone to help with cooking?

1. Yes
2. No

EXDB018 How long do you need someone to help you cook?

1. _______ [hc((0, 99], , ÿ)] **(EXDB018_1)** month
2. _______ [hc((0, 99], , ÿ)] **(EXDB018_2)**

Machine Translated by Google

3.Since ________ [hc([1, 120], , ÿ), sc((1, 100], , ÿ)] **(EXDB018_3)** years old

4.Since ________ [hc([1900, 2020], , ÿ), sc([1920, 2020], , ÿ)] **(EXDB018_4)** years

EXDB019 Did [XRName] have difficulty going to the store to buy groceries three months before his death due to health and memory problems?

Disaster?

1. No Difficulty
2. It is difficult but still achievable
3. Having difficulties and needing help
4. Unable to complete

EXDB020 Is there anyone who can help me buy groceries?

1. Yes
2. No

EXDB021How long does it take for someone to help him/her go to the store to buy groceries?

1. ________ [hc((0, 99], , ÿ)] **(EXDB021_1)** month

2. ________ [hc((0, 99], , ÿ)] **(EXDB021_2)** year

3.Since ________ [hc([1, 120], , ÿ), sc((1, 100], , ÿ)] **(EXDB021_3)** years old

4.Since ________ [hc([1900, 2020], , ÿ), sc([1920, 2020], , ÿ)] **(EXDB021_4)** years

EXDB022Did [XRName] have difficulty making phone calls in the three months before his death due to health and memory issues?

[Interviewer's note: If the deceased interviewee has never done this, and the family members judge that the deceased interviewee has done this because of health or memory problems,

If you cannot make a call due to the reason, select "I have difficulties and need help" or "I cannot complete the task" according to the actual situation.

1. No Difficulty
2. It is difficult but still achievable
3. Having difficulties and needing help
4. Unable to complete

EXDB023 Is there anyone who can help me when I call?

[Interviewer's note: If the deceased respondent has never called, select No.]

1. Yes
2. No

How long does it take for **EXDB024** to need help making a phone call?

1. ________ [hc((0, 99], , ÿ)] **(EXDB024_1)** month

2. ________ [hc((0, 99], , ÿ)] **(EXDB024_2)** year

3.Since ________ [hc([1, 120], , ÿ), sc((1, 100], , ÿ)] **(EXDB024_3)** years old

4.Since ________ [hc([1900, 2020], , ÿ), sc([1920, 2020], , ÿ)] **(EXDB024_4)** years

EXDB025 Did [XRName] have difficulty taking medicine on his own three months before his death due to health and memory problems?

Remember when and how much to eat

1. No Difficulty
2. It is difficult but still achievable
3. Having difficulties and needing help
4. Unable to complete

EXDB026 Is there anyone who can help me with taking medicine?

1. Yes

Machine Translated by Google

2. No

EXDB027How long does he/she need help taking medicine?

1. _______ [hc((0, 99], , , ÿ)] **(EXDB027_1)** month
2. _______ [hc((0, 99], , ÿ)] **(EXDB027_2)**
3. Since _______ [hc([1, 120], , ÿ), sc([1, 100], , ÿ)] **(EXDB027_3)** years old
4. Since _______ [hc([1900, 2020], , ÿ), sc([1920, 2020], , ÿ)] **(EXDB027_4)** years

EXDB028Did [XRName] have difficulty managing his own money in the three months before his death due to health and memory problems? ? For example , Bills, record expenses, manage finances?

1. No Difficulty
2. It is difficult but still achievable
3. Having difficulties and needing help
4. Unable to complete

EXDB029Is there anyone who can help me manage my money?

1. Yes
2. No

EXDB030How long does he/she need someone to help him/her manage money?

1. _______ [hc((0, 99], , , ÿ)] **(EXDB030_1)** month
2. _______ [hc((0, 99], , ÿ)] **(EXDB030_2)**
3. Since _______ [hc([1, 120], , ÿ), sc([1, 100], , ÿ)] **(EXDB030_3)** years old
4. Since _______ [hc([1900, 2020], , ÿ), sc([1920, 2020], , ÿ)] **(EXDB030_4)** years

EXD. Health Status and Function (III)

EXDB031Who helped [XRName] the most in the above difficulties ? (In dressing, bathing, eating, getting up, going to the toilet, cooking, Difficulties in shopping, making phone calls, taking medicine, managing money, etc.) (multiple choices are allowed)

[Interviewer Note: The option of nursing home residents is only for respondents who live in nursing homes or died in nursing homes]

1. Spouse
2. Parents, parents-in-law, father-in-law, mother-in-law
3. Children, daughters-in-law/sons-in-law, grandchildren
4. Brothers and sisters and their spouses and children, and brothers and sisters of [XRName]'s spouse and their spouses and children
5. Other relatives
6. Hired personnel (such as nannies), a total of _______ [hc((0, 99), , ÿ1), sc([1, 10), , ÿ1)] **(EXDB031_1)** positions
7. Volunteers or staff of voluntary organizations
8. Nursing home staff
9. Personnel of home-based elderly care service institutions
10. Help from the community
11. Other personnel, please specify _______ **(EXDB031_2)**

EXDB032 Among parents, parents-in-law, father-in-law, and mother-in-law, which ones help [XRName] ? (Multiple choices are allowed)

1. Father
2. Mother
3. Father-in-law
4. Mother-in-law

Machine Translated by Google

EXDB033 Help [XRName] 's children, daughters-in-law/sons-in-law, grandchildren/grandchildren. Which of the following children's families are they from? (Multiple choices are allowed)

1-25. [XEChildAliveName[ ]] 26-35. Other children, named **(EXDB033_1[i])** _______

EXDB034[ ] Who in [XEHelperChild[ ]]'s family personally helped [XRName] ? (Multiple selections allowed) 1. [XEHelperChild[ ]] himself

2. Spouse of [XEHelperChild[ ]] 3. Children

of [XEHelperChild[ ]], i.e. grandchildren of [XRName] , personally helping [XRName] 's [XE- , ÿ1)] **(EXDB034_1[i])**

HelperChild[ ]] has [hc((0, 99), children_______ , ÿ1), sc([1, 10),

EXDB035 Help [XRName] 's brothers and sisters and their spouses and children. [XRName] 's spouse's brothers and sisters and their spouses and children. Which of the following brothers and sisters' families are they from? (Multiple choices are allowed) 1-30. [XESibName[ ]] 31-40. Other brothers and sisters, whose names are **(EXDB035_1[i])**

EXDB036[ ] Who in the [XEHelperSib[ ]] family personally helped [XRName] ? (Multiple selections allowed)

1. [XEHelperSib[ ]] I

2. Spouse of [XEHelperSib[ ]] 3.

Children of [XEHelperSib[ ]], i.e. nieces and nephews of [XRName] , who personally help [XRName] , ÿ1)] **(EXDB036**

[XEHelperSib[ ]] has several children: [hc((0, 99), , ÿ1), sc([1, 10), **_1[i])**

EXDB037 has [hc((0, 99), , ÿ1), sc([1, 10), , ÿ1)] other relatives who personally helped [XRName]

Who are they from [XRName]? [Interviewer _______ **(EXDB037_1)**

note: If you don't know, please fill in -1]

EXDB038 Other people who personally helped [XRName] have a total of [hc((0, 99), , ÿ1), sc([1, 10), , ÿ1)]

Who are they from [XRName]? [Interviewer _______ **(EXDB038_1)**

note: If you don't know, please fill in -1]

EXDB039Of all the helpers listed below, please select the 7 types of people who helped [XRName] the most.

1-99. [XEHelper[ ]]

EXDB040[ ] In the month before [XRName]' s death, how many days did [XEHelpList[ ]] help [XRName] ? , ÿ1)] days 0, _______ [hc([31],

[Interviewer's note: If you don't know, please fill in -1]

EXDB041[ ] On the days when [XEHelpList[ ]] helped [XRName] , how many hours per day did he/she spend helping [XRName] ? [hc([0, 24], ,

_______ ÿ1)] hours [Interviewer's note: If it is less than one hour, please write 1; if you don't know, please write -1]

EXDB042[ ] [XEHelpList[ ]] Do you live with [XRName] while you are taking care of [XRName] ?

1. Yes

2. No

EXDB043 Does [XRName] use the following assistive tools? (Multiple selections are allowed) 1. Crutches

Machine Translated by Google

2. Walker 3.

Manual wheelchair

4. Electric

wheelchair 5. Catheter,

catheter bag 6.

Toilet 7. None of the above

[conflict(7, [7])]

EXE. Health Care and Insurance (I)

[Quote: We would now like to understand what health insurance or benefits [XRName] was enjoying before his death.]

EXEA001 Did [XRName] participate in the following medical insurance when he died? (Multiple choices are allowed) 1.

Urban employee basic medical insurance (medical insurance)

2. Urban and rural residents basic medical insurance (combined urban residents and new rural cooperative medical

insurance) 3. Urban residents basic medical

insurance 4. New rural cooperative medical insurance (cooperative

medical insurance) 5.

Public medical care 6.

Medical assistance 7. Commercial medical

insurance: purchased by the employer 8.

Commercial medical insurance: purchased by the

individual 9. Major disease

medical insurance for unemployed urban residents 10. Long-term care insurance 11.

Other medical insurance,

please specify **(EXEA001_1)** 12. No insurance [conflict(12, [12])]

EXEA001_verify Are you sure that [XRName] did not participate in any of the above medical insurances during his lifetime? [Interviewer Note: If the agent answers that [XRName] actually participated in the above insurances, please return to the previous question to make changes and select [XRName] during his lifetime.

1. Confirm that [XRName]

did not participate in any of the above medical insurances during his lifetime .

EXEA002 Did [XRName] participate in supplementary medical insurance before his death? (e.g. major illness medical insurance, etc.)

[Interviewer's note: "Supplementary medical insurance" means that since the country's basic medical insurance can only meet the basic medical needs of the insured, medical needs beyond the scope of basic medical insurance can be supplemented by supplementary medical insurance. It is relative to basic medical insurance and includes various forms such as enterprise supplementary medical insurance, commercial medical insurance, social mutual assistance and community medical insurance.]

1. Yes

2. No

EXEA003[ ] Where was [XEXMIns[ ]] of [XRName] obtained? 1. General

residence before death: [EXB003_1] 2. (If the household

registration is not in the county/city of general residence) Place of

household registration 3. Other **(EXEA003_1[i])** Province/city/county

EXEA008[ ] When did [XRName] participate in [XEXMIns[ ]]?]) year [hc([1, 12], , ________ [hc([1900, 2020], ,)] **(EXEA008_1[i**

ÿ1)] **(EXEA008_2[i])** month

Machine Translated by Google

[Interviewer's note: If the respondent is not sure about the insurance enrollment time, please roughly estimate the insurance enrollment year. Use 4 digits to represent the year, and fill in the month according to the actual month. For example: January is written as "1" instead of "01", and December is written as "12". If you can't remember the month, please fill in "-1"]

EXEA009 What was the main reason why [XRName] did not participate in any medical insurance before his death (multiple choices)? 1. No need 2. Thought the insurance premium was too expensive 3. Did not know where to apply 4. Did not trust the health insurance agency 5. No suitable insurance program 6. Never thought about this issue 7. Other reasons **(EXEA009_1)** ________

EXEB001 Has [XRName] ever participated in the following medical insurance? (Multiple choices are allowed)

1. Basic medical insurance for urban employees (medical insurance) 2. Basic medical insurance for urban and rural residents (combined urban residents and new rural cooperative medical insurance) 3. Basic medical insurance for urban residents 4. New rural cooperative medical insurance (cooperative medical insurance) 5. Public medical care 6. Medical assistance 7. Commercial medical insurance: purchased by the employer 8. Commercial medical insurance: purchased by the individual 9. Major disease medical insurance for unemployed urban residents 10. Long-term care insurance 11. Other medical insurance, ________ please specify **(EXEB001_1)** 12. No insurance [conflict(12, [12])]

EXEB003[ ] When will [XRName] withdraw from [XEXPMIns[ ]] ? **i])** year [hc([1, 12], , ÿ1)] ________ [hc([1900, 2020], ,)] **(EXEB003_1[**

(EXEB003_2[i]) month [Interviewer's note: If the respondent is not sure when he/she will withdraw from the insurance, please roughly estimate the year of withdrawal. Use 4 digits to represent the year and fill in the month according to the actual month. For example: January is written as "1" instead of "01", and December is written as "12". If you cannot remember the month, please fill in "-1".]

EXEB004[ ] Why did [XRName] quit [XEXPMIns[ ]]? 1. The company no longer exists 2. The local area no longer provides this insurance 3. I resigned from the company/was fired 4. I don't want to participate 5. My family doesn't want me to participate 6. The premium is too expensive 7. Others, please specify **(EXEB004_1[i])**

____

EXEB005 After [XRName] passed away, did family members or friends withdraw money from the medical insurance account?

1. Yes 2. No

EXEB006 How much money is this? [hc([0, ÿ), ________ , ÿ1), sc([0, 50000), , ÿ1)]

Machine Translated by Google

EXE. Health Care and Insurance (II)

EXEC001Between the time of his last visit and the time of his death, when was [XRName]'s last routine physical examination?

ÿInterviewer's note: Routine physical examination is a comprehensive health checkup to help you understand your health status. Going to the hospital to only measure blood pressure, blood sugar or

Blood tests done just for checkup purposes are not considered routine physical examinations. Use 4 digits to indicate the year. If you cannot remember the time of your routine physical examination, please estimate it roughly.

Calculate the year of the physical examination. Fill in the month according to the actual month. For example, January is written as "1" instead of "01", and December is written as "12". If you can't remember the month,

Please fill in "-1"]

1. ________ [hc([1900, 2020], ,)] **(EXEC001_1)** year [hc([1, 12], 2. No routine physical examination , ÿ1)] **(EXEC001_2)** Month since the last visit

EXED030In addition to being hospitalized, did [XRName] visit a medical institution for outpatient treatment or receive home medical services in the month before his death?

several times? ________ [hc([0, ÿ), ,), sc([0, 10], ,)] times

EXED031 In addition to being hospitalized, did [XRName] visit a medical institution for outpatient care or receive home medical services in the month before his death?

What is the total cost? [hc([0, ÿ), 00, 1000])] yuan , ÿ1), sc([0, 30000], , ÿ1), ub([ÿ1], [50, 100, 200, 5

[Interviewer's note: If the respondent cannot answer, please fill in "-1".]

EXED032How much of these medical expenses did [XRName] pay out of pocket?

[Interviewer's note: If the respondent cannot answer, please fill in "-1".]

1. The self-paid portion is [hc((0, ÿ), 00, 800])] , ÿ1), sc((0, 30000], , ÿ1), ub([ÿ1], [50, 100, 250, 5
   (EXED032_1) yuan
2. No money was paid

EXED033 Which medical insurance is used or will be used to reimburse the medical expenses (including medical treatment fees and drug costs)? (Multiple choices are allowed)

1-11. [XEXMIns[ ]]

12. Reimbursement by the unit
13. No Insurance
14. Not Applicable

One month before **EXEF001's** death, did [XRName] buy his own medicine?

[Interviewer's note: This does not include the situation of taking medicine with a prescription, but taking medicine given by others or medicine stored by oneself is also considered as buying medicine for oneself.]

1. Yes
2. No

EXEF002 How much did [XRName] spend on his own medicine in the month before his death ? Include the total amount of money paid out of pocket and reimbursed.

________ [hc([0, ÿ), , ÿ1), sc([0, 2000], , ÿ1), ub([ÿ1], [10, 30, 100, 200, 300])]

[Interviewer's note: If the respondent cannot answer, please fill in "-1".]

EXEF003In addition to the reimbursement, how much did [XRName] pay out of pocket?

[Interviewer's note: If the respondent cannot answer, please fill in "-1".]

1. The self-paid part is [hc((0, ÿ), 200])] **(EXEF003_1)** , ÿ1), sc((0, 2000], , ÿ1), ub([ÿ1], [10, 30, 70, 100,
   yuan
2. No money was paid

EXEF005 [XRName] Which medical insurance did you use to reimburse for self-treatment? (Multiple choices are allowed)

1-11. [XEXMIns[ ]]

12. Reimbursement by the unit

Machine Translated by Google

13. No Insurance
14. Not Applicable

EXEG000 Did [XRName] die in the hospital?

1. Yes
2. No

EXEG001 You previously told us that [XRName] died in a hospital. How long was he a patient in that hospital before he died?

between?

[Interviewer's note: If it is less than 1 hour, please fill in 1 hour]

1. ______ [hc([0, 24), , , ÿ1)] **(EXEG001_1)** hours
2. ______ [hc([1, 7), , ÿ1)] **(EXEG001_2)** days
3. ______ [hc([1, 5), , ÿ1)] **(EXEG001_3)** week
4. ______ [hc([1, 12), ÿ1)] **(EXEG001_4)** month
5. ______ [hc([1, ÿ), ÿ1)] **(EXEG001_5)** years

EXEG002 Why was [XRName] hospitalized? Surgery, other treatment, some form of symptom relief, or something else?

1. Surgery
2. Other treatments
3. Alleviate symptoms
4. Others **(EXEG002_1)** ______

EXEG003 In addition to the hospitalization during which [XRName] died, was he hospitalized again in the month before his death?

1. Yes
2. No

EXEG004 Was [XRName] hospitalized in the year before his death?

1. Yes
2. No

EXEG005 How many times was [XRName] hospitalized in the year before his death (including the hospitalization at the time of his death)? ______ [hc((0, ÿ), ,)] times

EXEG006Did [XRName] stay in the ICU while hospitalized ?

1. Yes, the total number of days in the ICU in the year before death is ______ [hc([1, 366], , ÿ1), sc([1, 180], , ÿ1)] **(EXEG006_1)** days
2. No

EXEG007While in hospital, did [XRName] use any life support equipment, such as a ventilator, artificial liver, or artificial lung? (Multiple choices are allowed)

1. Respirator
2. Artificial liver
3. Artificial Lung
4. None of the above

[conflict(4, [4])]

EXEG008Did [XRName] undergo renal dialysis while in hospital?

1. Yes
2. No

EXEG009While in the hospital, did [XRName] receive antibiotics for pneumonia or other infectious diseases?

Machine Translated by Google

1. Yes

2. No

EXEG010 What was the total cost of all hospitalization expenses of [XRName] in the year before his death, including only the fees paid to the hospital, excluding the salary of the attendant, the transportation and accommodation expenses of himself or his family, but including the hospital ward fee? [hc([0, ÿ), , 1), sc([0, 300000], , ÿ1), ub([ÿ1], [1500, 3000, 7000, 15000, 30000])] yuan

[Interviewer's note: If the respondent cannot answer, please fill in "-1".]

EXEG011 Of these hospitalization expenses, how much did [XRName] pay out of pocket?

[Interviewer Note: If the respondent cannot answer, please fill in "-1".] 1. Out-of-pocket portion [hc((0, ÿ), , ÿ1), sc((0, 300000], , ÿ1), ub([ÿ1], [1000, 2000, 5 000, 10000, 20000])] **(EXEG011_1)** yuan 2. Did not pay anything

EXEG012 Which medical insurance is used or will be used to reimburse hospitalization expenses? (Multiple choices are allowed)

1-11. [XEXMIns[ ]] 12. Reimbursed by the employer 13. No insurance 14. Not applicable

EXF. Work and retirement

EXF001 According to our records, [XRName] was working at [ZIWTime] during the last survey . What year and month was [XRName]? Stopped working?

[Interviewer Note: If the respondent cannot answer the year and month, please fill in "-1".]

1. ________ [hc([ZIWYear,XIWYear], , ÿ1)] **(EXF001_1)** year [hc([1, 12], , ÿ1)] **(EXF001_2)** month2 .

[XRName] worked until his death995 .

[XRName] was not working in the last round of survey997 .

Don't know999 .

Refuse to answer

EXF002 How many days did [XRName] work the week before [XRName] stopped working? [hc ([1, 7], , ÿ1)] day

[Interviewer's note: If the respondent cannot answer, please fill in "-1".]

EXF003 During the days that [XRName] worked this week , how many hours did [XRName] work on average each day? [hc([1, 24], , ÿ)] **(EXF003_1)** hours/day, including: agricultural activities [hc([0,EXF003_1], , ÿ1)]), sc([1, 16], **(EXF003_2)** hours/day; non-agricultural activities [hc([0,EXF003_1], , ÿ1)] **(EXF003_3)** hours/day. [Interviewer's note: The sum of agricultural and non-agricultural working hours should be equal to the total working hours. If the respondent cannot answer, please fill in "-1".]

EXF004 According to our records, [XRName] had not yet completed retirement procedures when we last investigated . What happened to [XRName] when he passed away ? Have you completed the retirement procedures before? 1. Yes 2. No 997. Don't know 999. Refuse to answer

Machine Translated by Google

EXFN. Pension

EXF005 How much money was withdrawn from the pension or retirement insurance account after [XRName] passed away? [hc([0, 10000000] , , ÿ1)] yuan

[Interviewer's note: If the agent cannot answer, please fill in "-1".]

EXF006 After [XRName] passed away, did he receive a lump sum payment or funeral expenses from his pension insurance or old-age pension?

1. Yes 2.

No

EXF007 How much is this money? [hc([10, 10000000], , ÿ1)] yuan [Interviewer's note: If the agent cannot answer, please fill in "-1".]

EXF008 After [XRName] passed away, did you receive a lump sum pension or funeral benefits from somewhere other than retirement/pension insurance? Fee?

1. Yes 2.

No

EXF009 How much money is this? [hc([10, 10000000], , ÿ1)] yuan

ÿInterviewer's note: If the agent cannot answer, please fill in "-1". ÿ

EXF010 Did [XRName] have life insurance? 1. Yes 2. No

EXF011 How much did the life insurance pay out after [XRName] died? [hc([10, 10000000], , ÿ1)] yuan

ÿInterviewer's note: If the agent cannot answer, please fill in "-1". ÿ

EXF012 Did [XRName] have any commercial pension insurance before his death?

1. Yes 2. No

EXF013 How much money did [XRName] withdraw from the commercial pension insurance after his death? [hc([10, 10000000], , ÿ1)] yuan [Interviewer's note: If the agent cannot answer, please fill in "-1".]

EXG. Income, Expenses and Assets

EXG001 When [XRName] died, did he own any property? If he did, what was the total value of the property he owned? If he only had partial ownership, calculate the value of the property he owned. If he had multiple properties, calculate the total value of the properties. [Interviewer's note: If the respondent refuses to answer or forgets, fill in "-1".] 1. He has real estate, with a total value of [hc((0, ÿ), , ÿ1), sc((0, 1000000], , ÿ1)] **(EXG001_1)** yuan 2. He does not have any real estate

EXG002 How is [XRName] 's property distributed? What is their respective inheritance ratio? If the property is sold, please also calculate Calculate the proportion of each allocation (multiple choices are allowed). [Interviewer's note: If the respondent refuses to answer or forgets, fill in "-1".]

1. Spouse [hc((0, 100], 2. Children, son-in- , ÿ1)] **(EXG002_1)** %
law, daughter-in-law

Machine Translated by Google

3. Brothers and sisters [hc((0, 100], , ÿ1)] **(EXG002_3)** %
4. Other relatives [hc((0, 100], , ÿ1)] **(EXG002_4)** %
5. Parents, parents-in-law [hc((0, 100], , ÿ1)] **(EXG002_5)** %
6. Grandchildren
7. Friends [hc ((0, 100], , ÿ1)] **(EXG002_7)** %
8. Charity [hc((0, 100], , ÿ1)] **(EXG002_8)** %
9. Other, please specify **(EXG002_9_1) ,** 10. Not allocated [hc((0, 100], , ÿ1)] **(EXG002_9)** %

[conflict(10, [10])]

EXG003Which children/children's spouses will inherit the property? The children's spouses are counted as children.

In the options, please add the names of [XRName] 's children (multiple selections are allowed).

1-25. [ZChildName[ ]]

26-35. Other children, named **(EXG003_1[i])**

EXG004[ ] [XEXGChildList1[ ]] and his spouse receive a percentage of the house value of [hc((0, 100], , ÿ1)] %.

[Interviewer's note: If the respondent refuses to answer or forgets, please fill in "-1".]

EXG007 Whose children are the grandchildren/grandchildren-in-law? If the father of the grandchildren/mother of the grandchildren who inherit the property is not present

In the children option, please add the names of [XRName] 's children (multiple selections are allowed).

1-25. [ZChildName[ ]]

26-35. Others, children's names are **(EXG007_1[i])**

EXG008[ ] [XEXGChildList4[ ]]'s child gets the percentage of the house value [hc((0, 100], , ÿ1)] %.

[Interviewer's note: If the respondent refuses to answer or forgets, please fill in "-1".]

EXG009 In addition to the aforementioned real estate, medical insurance accounts, retirement/pension accounts and related pensions, other pensions or funeral expenses

In addition to the medical insurance premiums, life insurance claims and commercial pension insurance, did [XRName] leave any cash after his death?

1. Yes
2. No

EXG010 How much cash did [XRName] leave behind after his death? [hc((0, ÿ), , ÿ1), sc((0, 100000), , ÿ1)] yuan

[Interviewer's note: If the respondent refuses to answer or forgets, please fill in "-1".]

EXG011 In addition to the aforementioned real estate, medical insurance accounts, retirement/pension accounts and related pensions, other pensions or funeral expenses

In addition to the fees, life insurance claims and commercial pension insurance, did [XRName] leave any savings after his death?

1. Yes
2. No

EXG012 How much money did [XRName] leave behind after his death? [hc((0, ÿ), , ÿ1), sc((0, 100000), , ÿ1)] yuan

[Interviewer's note: If the respondent refuses to answer or forgets, please fill in "-1".]

EXG013 In addition to the aforementioned real estate, medical insurance accounts, retirement/pension accounts and related pensions, other pensions or funeral expenses

In addition to the insurance premiums, life insurance claims and commercial pension insurance, did [XRName] leave any stocks, funds, or treasury funds after his death?

Securities and other financial assets?

1. Yes
2. No

Machine Translated by Google

EXG014 How many financial assets such as stocks, funds, and treasury bonds did [XRName] leave behind after his death? [hc((0, ÿ), , ÿ1

), sc((0, 100000), , ÿ1)] yuan

[Interviewer's note: If the respondent refuses to answer or forgets, fill in "-1".]

EXG015 In addition to the aforementioned real estate, medical insurance accounts, retirement/pension accounts and related pensions, other pensions or funeral expenses

In addition to the medical insurance premiums, life insurance claims and commercial pension insurance, did [XRName] leave any physical objects after his death?

1. Yes
2. No

EXG016 How much is the value of the physical objects left behind after [XRName] dies? [hc((0, ÿ), , ÿ1), sc((0, 100000), , ÿ1)]

Yuan [Interviewer's note: If the respondent refuses to answer or forgets, please fill in "-1".]

EXG017 In addition to the aforementioned real estate, medical insurance accounts, retirement/pension accounts and related pensions, other pensions or funeral expenses, life insurance compensation, commercial pension insurance, cash, deposits, financial assets, and physical objects, did [XRName] leave any other property after his death? If so, how much? [hc([0, ÿ), , ÿ1), sc([0, 100000), , ÿ1)] yuan [Interviewer's note: If the respondent refuses to answer or forgets, fill in "-1".]

EXG018 In addition to real estate, [XRName] left the following inheritance: medical insurance accounts, retirement/pension and related pensions, funeral expenses, life insurance compensation, cash, deposits, stock funds, treasury bonds and other financial assets, physical value and others. Is the total [XEXGTotalValue]

correct? [Interviewer Note: If the respondent refuses to answer or forgets, fill in "-1".]

1. Yes
2. No, it should be [hc([0, ÿ), , ÿ1), sc([0, 200000], , ÿ1)] **(EXG018_1)**

EXG019 Before [XRName] passed away, did he/she make a will? 1. Yes 2. No

EXG020 Has [XRName] 's will been notarized? 1. Yes 2. No

EXG021 In [XRName] 's will, how did he/she distribute his/her estate? Excluding real estate (multiple selections allowed).

[Interviewer Note: If the respondent refuses to answer or forgets, please fill

1. Spouse [hc((0, 100], 2. Children, son- in "-1".] , -1)] **(EXG021_1)** %

in-law, daughter-in-law 3. Brothers

and sisters [hc((0, 100], , ÿ1)] **(EXG021_3) %** 4. Other relatives [hc((0, 100], , ÿ1)]

(EXG021_4) % 5. Parents, parents-in-law [hc((0, 100], , ÿ1)] **(EXG021_5)** % 6.

Grandchildren 7. Friends [hc((0, 100], , ÿ1)] (EXG021_7) % 8. Charitable organizations

[hc((0, 100], , ÿ1)] **(EXG021_8)**

% 9. Others, please specify **(EXG021_9_1) ,** [hc((0, 100], 10. [conflict(10,

[10])] is not assigned

, ÿ1)] **(EXG021_9)** %

EXG022 Which children/spouse of children are they? The spouse of the children is counted as a child. If the children mentioned in the will are not included in the options,

Machine Translated by Google

Please add the names of [XRName] 's children (multiple selections allowed).

1-25. [ZChildName[ ]]

26-35. Others, children's names are (EXG022_1[i])

EXG023[ ] [XEXGChildList2[ ]] and his spouse receive the following percentage of the estate in the will: [hc((0, 100], , ÿ1)] %.

[Interviewer's note: If the respondent refuses to answer or forgets, please fill in "-1".]

EXG026 Whose children are the grandchildren/grandchildren? If the father of the grandchildren/mother of the grandchildren mentioned in the will is not In the options, please add the names of [XRName] 's children (multiple selections are allowed).

1-25. [ZChildName[ ]]

26-35. Others, children's names are (EXG026_1[i])

EXG027[ ] [XEXGChildList5[ ]]'s children receive a percentage of the estate in the will [hc((0, 100], , ÿ1)] %.

[Interviewer's note: If the respondent refuses to answer or forgets, please fill in "-1".]

EXG028In a will, is the inheritance given to brothers and sisters divided equally among them?

1. Yes
2. No

EXG029 Of all the estate left by [XRName] , minus funeral expenses, is there any estate left to inherit? The last part to be inherited What is the value? Excluding property

[Interviewer's note: If the respondent refuses to answer or forgets, please fill in "-1".]

1. There is also an inheritance, valued at [hc((0, ÿ), , ÿ1), sc((0, 200000], , ÿ1)] **(EXG029_1)** yuan
2. No inheritance

EXG030 When dividing [XRName] 's estate, did you follow his/her will completely?

1. Yes
2. No

EXG031 How was [XRName] 's estate distributed? Excluding real estate (multiple selections allowed).

[Interviewer's note: If the respondent refuses to answer or forgets, please fill in "-1".]

1. Spouse [hc((0, 100], 2. Children, son-in-law, daughter-in-law , ÿ1)] **(EXG031_1)** %
3. Brothers and sisters [hc((0, 100], , ÿ1)] **(EXG031_3)** %
4. Other relatives [hc((0, 100], , ÿ1)] **(EXG031_4)** %
5. Parents, parents-in-law [hc((0, 100], , ÿ1)] **(EXG031_5)** %
6. Grandchildren
7. Friends [hc ((0, 100], , ÿ1)] **(EXG031_7)** %
8. Charity [hc((0, 100], , ÿ1)] **(EXG031_8)** %
9. Other, please specify **(EXG031_9_1) ,** 10. No allocation [hc((0, 100], , ÿ1)] **(EXG031_9)** %

[conflict(10, [10])]

EXG032 Which children/spouse of children are they? Spouses of children are counted as children. If the children who inherit the estate are not included in the selection, please Add the names of [XRName] 's children (multiple selections allowed).

1-25. [ZChildName[ ]]

Machine Translated by Google

26-35. Others, children's names are **(EXG032_1[i])**

EXG033[ ] [XEXGChildList3[ ]] and his spouse receive a percentage of the estate of [hc((0, 100], , ÿ1)] %.

[Interviewer's note: If the respondent refuses to answer or forgets, please fill in "-1".]

EXG036 Whose children are the grandchildren/grandchildren-in-law? If the father of the grandchildren/mother of the grandchildren who inherit the estate is not alive

In the children option, please add the names of [XRName] 's children (multiple selections are allowed).

1-25. [ZChildName[ ]] 26-35.

Others, children's names are **(EXG036_1[i])**

EXG037[ ] [XEXGChildList6[ ]] The percentage of inheritance received by the children is [hc((0, 100], , ÿ1)] %.

[Interviewer's note: If the respondent refuses to answer or forgets, fill in "-1".]

EXK. Funeral

K01 What is the burial method of [XRName] ?

1.

Cremation

2. Burial 3.

Sky burial

4. Water burial 5. Other, please specify **(K01_1)**

K02 Where is [XRName] buried?

1. Own contracted land

2. Village collective

cemetery 3. Commercial

cemetery

4. Wasteland 5. Ashes stored

in funeral home 6. Other, please specify **(K02_1)**

K03 How much did the grave/cemetery cost? If no money was spent, please

fill in 0. 1 . _______ [hc([0, ÿ), , ÿ1), sc([0, 50000), , ÿ1)] **(K03_1)**

2. _______ [hc([0, ÿ), , ÿ1), sc([0, 30000), , ÿ1)] **(K03_2)** yuan/year

K04 In which year was the grave/cemetery purchased? [hc([1900, 2020], ,)]

K06 Who paid for the cemetery? Multiple choices are allowed.

1. [XRName] 's spouse

2-26. [XKChildName[ ]]

27-51. [XKChildAndS[ ]] 52.

Grandson 53.

Granddaughter 54.

Relatives

55. Workplace (including village

collective)

56. Insurance 57. Others

Machine Translated by Google

K07 K06 If you selected grandchild or grandchild, please specify which child of [XRName] is this grandchild or grandchild.

parents?

1-25. [ZChildName[ ]]

K08 If you chose relative or other person among K06, please specify who he/she is to the respondent. ________

K09 How much did [XRName] 's funeral cost in total? Including the cost of the coffin/urn, the portrait, the funeral ceremony, the ritual, etc., , ÿ1), ub([ÿ1],

Excluding cemetery/funeral home fees. Total [hc([0, ÿ), 0, 3000, 5000, , ÿ1), sc([0, 100000), [100

8000, 10000])] yuan

K10 Who pays for the funeral expenses? You can select multiple options.

1. [XRName] 's spouse 2-26.

[XKChildName[ ]]

27-51. [XKChildAndS[ ]] 52. Grandson

53. Granddaughter 54.

Relatives 55. Workplace

(including

village collective) 56. Insurance 57.

Others

K11 K10 If you selected grandchild or grandchild, please specify which child of [XRName] is this grandchild or grandchild.

parents?

1-25. [ZChildName[ ]]

K12 If you chose relative or other person among K10, please specify who he/she is to the respondent. ________

How much money did **K13** receive as a gift when [XRName] was buried? Total [hc([0, ÿ), , ÿ1), sc([0, 50000], ,

1), ub([ÿ1], [1000, 3000, 5000, 8000, 10000])]

EXV. Epidemic related

EXV001 Was [XRName] ever diagnosed with COVID-19 before his death?

1. Yes
2. No

999. Refuse to answer

EXV002 Was [XRName] identified as a suspected case of COVID-19 before his death?

1. Yes
2. No

999. Refuse to answer

EXV003 Was [XRName] hospitalized for treatment of COVID-19 ? He stayed in the hospital for a total of [hc([0, 250], [Interviewer , ÿ1)] day?

Note: If not hospitalized, fill in "0". If the respondent cannot answer, please fill in "-1".]

EXV004 [XEXVAHospitalizationExcluded] Has [XRName] ever been isolated or placed under medical observation for the following reasons? Home quarantine and building closure are both considered quarantine. (Multiple choice question)

1. Travel or business trip (including going to work somewhere after the Spring Festival)

Machine Translated by Google

2. Close contact with COVID-19 cases 3.

Residence or residential building is closed (excluding community

closure) 4. Required to be isolated when going to a medical institution

for treatment or after discharge 5.

Nucleic acid test shows positive 6. No isolation

experience (exclusive

option) 997. Don't know

999. Refuse to answer [conflict(6, 997, 999, [6, 997, 999])]

EXV005 [XRName] has been isolated for a total of [hc([1, 250], ________ , ÿ1), sc([1, 14], , ÿ1)] day?

[Interviewer's note: If there are multiple quarantine experiences, please record the total duration. If the respondent cannot answer, please fill in "-1".]

EXV006 Where is [XRName] isolated? (Multiple choice) 1. Hospital 2. Hotel or

other

centralized medical observation point 3. Own

residence 4. Other:

999. Refuse ________ **(EXV006_1)**

to answer [conflict(999,

[999])]

EXV007 During [XRName] 's quarantine experience, was [XRName] ever accompanied by others in the quarantine residence? (Interviewer's note: If there are multiple quarantine experiences, as long as there is one quarantine experience in which someone else accompanied him/her, it can be considered "yes")

1. Yes 2.

No 999.

Refuse to answer

EXV008 Has [XRName] been tested for COVID-19?

[Interviewer's note: If the respondent cannot answer the month, please fill in "-1".]

1. Yes, the last test time was [hc([1, 12], 2. No 999. Refuse to answer ________ , ÿ1)] **(EXV008_1)** Month

EXV009During the epidemic, [XEXVCNotInQuarantine] , [XRName] , have you ever stayed at home for several days because you were worried about contracting the disease?

[XRName] How many days in a row have you stayed at home? [hc([0, 250], ________ , ÿ1)] days (if you go out every day, fill in "0") [Interviewer's note: If the respondent cannot answer, please fill in "-1".]

EXV010During the epidemic, did [XRName] ever need to see a doctor, including a dentist, but was forced to postpone or not see a doctor due to the epidemic?

Can I go and

see? 1.

Yes 2. No

EXV011Why was [XRName]' s visit to the doctor delayed or not possible? (Multiple choice question, optional) 1. Unable to make an

appointment, or the hospital's regular appointments were cancelled 2.

The hospital rescheduled the regular treatment

arrangements 3. [XRName] decided to wait 4. [XRName] was

afraid of going to the hospital 5. Other,

please specify **(EXV011_1)** ________

Machine Translated by Google

EXV012 Could you please explain in detail what disease [XRName] wanted to see or what medical service he wanted during the epidemic?

Was it postponed or cancelled? (Multiple-choice

question) [Interviewer's note: Please read out each option and check all that apply]

1. Major surgery that requires hospitalization 2.

Minor surgery that can be done in an outpatient clinic or day ward 3. Going to see a doctor

for a general outpatient clinic 4. Going to

get prescription drugs 5. Going

to the dentist, oral treatment 6. Others,

please briefly explain **(EXV012_1)** ________

EXV013 [XRName] wanted to see a doctor during the epidemic because of new symptoms or diseases, or because he wanted to treat an existing disease.

Diseases that have emerged, or routine physical examinations and

screenings? (Multiple choice) 1. New symptoms

or diseases 2. Treatment of existing diseases

3. Routine physical examinations and screenings

EXV014 In your opinion, is [XRName]'s death related to the COVID-19 epidemic? (Multiple choice question)

1. Died due to confirmed infection with COVID-19

2. Unable to receive routine diagnosis and treatment in time due to the

epidemic 3. Unable to receive emergency treatment in time due to the

epidemic 4. Unable to receive nursing and care due to the epidemic 5.

Accident caused by the epidemic 6. Other

reasons, please briefly explain **(EXV014_1)** ________ 7. Death is not related to the epidemic

[conflict(7, [7])]

VA. Cause of Death Analysis

EX004 Name of the agent who answered the exit questionnaire ________

EX005 What is the relationship between [XRName] and the agent? 1.

Father 2.

Mother 3.

Spouse 4.

Brothers and sisters 5.

Other relatives (please specify) 6. No ________ **(EX005_1)**

relationship

EX006 Contact information of the agent who answered the questionnaire ________

VAS41 Can you tell me what disease or event caused [XRName] 's death? If there are relevant medical documents ________

(excluding death certificates, which have been asked), such as medical records, doctor's diagnosis, etc., please take photos of key medical documents after obtaining

permission. **(VAS41_1)** ________

VAS42 Can you tell me what was the number one cause of death for [XRName] ? ________

If there are relevant medical documents (excluding death certificates and documents collected in the previous question, which have been asked), such as medical records,

doctor's diagnosis, etc., please take photos of key medical documents after obtaining permission. **(VAS42_1)** ________

Machine Translated by Google

VAS43 Can you tell me what the second cause of death of [XRName] was? ________

[Interviewer's note: If the respondent cannot answer, please fill in "-1".]

Auxiliary variable definition

XRDeathTime died on: year, month, day

```
if (!empty("XRDeathYear") && !empty("XRDeathMonth" ) && !empty("XRDeathDate") && !
      equal("XRDeathMonth", "-1") && !equal("XRDeathDate", "-1" ))
      { add("XRDeathTime", value("XRDeathYear")+"Year"+value("XRDeathMonth")+"Month"+value("XRDeathDate")+"Day")
}

if (empty("XRDeathYear") || empty("XRDeathMonth") || empty("XRDeathDate") || equal("XRDeathMonth", "-1") ÿ ||

 equal("XRDeathDate", "-1" ))
      { add("XRDeathTime", value("EXB001_1")+"year"+value("EXB001_2")+"month"+value("EXB001_3")+"day")
}
```

XAliveResidenceFull General residence before death Option 1. Mainland China Level 5 address + house number

```
if (equal("EXB003", "1")) {
      add("XAliveResidenceFull",value("EXB003_1")+value("EXB003_2")+value("EXB003_3"))
}
```

XEZDisease generates the disease status of the previous period based on the loaded variables

```
if (equal("ZDisease[4]", "1"))
      { add("XEZDisease[1]", "1")

} if (equal("ZDisease[5]", "1"))
      { add("XEZDisease[2]", "1")

} if (equal("ZDisease[7]", "1"))
      { add("XEZDisease[3]", "1")

} if (equal("ZDisease[8]", "1"))
      { add("XEZDisease[4]", "1")

} if (equal("ZDisease[11]", "1"))
      { add("XEZDisease[5]", "1")
}
```

XEChroDisType chronic disease typeadd

```
("XEChroDisType", ["cancer and other malignant tumors (excluding mild skin cancer)", "chronic lung diseases such as chronic bronchitis or emphysema, cor pulmonale
 ÿ      (excluding tumors or cancer)", "heart disease (such as myocardial infarction, coronary heart disease, angina pectoris, congestive heart failure and other heart
 ÿ      diseases)", "stroke", "emotional and mental problems"])
```

XEDisease[3] Whether you have heart disease

```
if ( equal("EXDA001[3]", "1") || equal("XEZDisease[3]", "1") )
      { add("XEDisease[3]", "1") }
else
      { add( "XEDisease[3]", "2")
}
```

XEDisease[1] Whether you have cancer

```
if (equal("EXDA001[1]", "1") || equal("XEZDisease[1]", "1"))
      { add("XEDisease[1]", "1") }
else
      { add( "XEDisease[1]", "2")
}
```

XEDisease[4] Whether you have suffered a stroke

```
if (equal("EXDA001[4]", "1") || equal("XEZDisease[4]", "1"))
      { add("XEDisease[4]", "1") }
else {
```

Machine Translated by Google


```
        add("XEDisease[4]", "2")
    }
```

XEHelperSelect Does anyone help

```
    if (equal("EXDB002", "1") || equal ("EXDB005", "1" ) || equal("EXDB008 ", " 1") || equal("EXDB011", "1") || equal("EXDB014",
ÿ       "1") || equal ("EXDB017", "1" ) || equal("EXDB020", " 1") || equal("EXDB023", "1") || equal( "EXDB026", "1") ||
ÿ       equal("EXDB029", "1") ) { add("XEHelperSelect", "1") } else
        { add("XEHelperSelect", "0")


    }
```

XEHelperChild provides a list of children's names to help

```
    for (var i1 = 1; i1 <= 25; i1++)
        { add("XEHelperChild[i1]", value("XEChildAliveName[i1]"))

    } for (var i1 = 26; i1 <= 35; i1++)
        { add("XEHelperChild[i1]", value("EXDB033_1[i1]"))
    }
```

XESibName Generates a list of siblings' names Generates a list of siblings' names

```
    for (var i1 = 1; i1 <= 15; i1++) { if (!
        empty("ZSibName[i1]"))
            { add("XESibName[i1]", pre("XRName")+"ÿÿÿ"+pre("ZSibName[i1]"))
        }

    } for (var i1 = 1; i1 <= 15; i1++) { if (!
        empty("ZSibNameS[i1]")) {
            add("XESibName["+(i1+15)+"]", pre("XRName")+"spouse's brothers and sisters"+pre("ZSibNameS[i1]"))
        }
    }
```

XEHelperSib provides the name of the sibling who is helping

```
    for (var i1 = 1; i1 < 31; i1++)
        { add("XEHelperSib[i1]", value("XESibName[i1]"))

    } for (var i1 = 31; i1 <= 40; i1++)
        { add("XEHelperSib[i1]", value("EXDB035_1[i1]"))
    }
```

XEHelperNum provides the number of helpers

```
    add("XEHelperNum", "0") if
    (selected("EXDB031", "1"))
        { add("XEHelperNum", value("XEHelperNum")+1)
        add("XEHelper["+value("XEHelperNum ")+"]", "spouse")

    } if (selected("EXDB031", "5"))
        { add("XEHelperNum", value("XEHelperNum")+1)
        add("XEHelper["+value("XEHelperNum")+"]", "Other relatives")
    }

    if (selected("EXDB031", "6"))
        { add("XEHelperNum", value("XEHelperNum")+1)
        add("XEHelper["+value("XEHelperNum")+"]", "Hire personnel")
    }

    if (selected("EXDB031", "7"))
        { add("XEHelperNum", value("XEHelperNum")+1)
        add("XEHelper["+value("XEHelperNum")+"]", "Volunteer ")
    }

    if (selected("EXDB031", "8"))
        { add("XEHelperNum", value("XEHelperNum")+1)
        add("XEHelper["+value("XEHelperNum")+"]", "Nursing home staff")
    }

    if (selected("EXDB031", "9"))
        { add("XEHelperNum", value("XEHelperNum")+1)
```

Machine Translated by Google


```
        add("XEHelper["+value("XEHelperNum")+"]", "Staff of home-based elderly care service organization")
}

if (selected("EXDB031", "10"))
        { add("XEHelperNum", value("XEHelperNum")+1)
        add("XEHelper["+value("XEHelperNum")+"]", "Community" )
}

if (selected("EXDB031", "11"))
        { add("XEHelperNum", value("XEHelperNum")+1)
        add("XEHelper["+value("XEHelperNum")+"]", "Other personnel")

} if (selected("EXDB032", "1"))
        { add("XEHelperNum", value("XEHelperNum")+1)
        add("XEHelper["+value("XEHelperNum")+"]", "Father ")

} if (selected("EXDB032", "2"))
        { add("XEHelperNum", value("XEHelperNum")+1)
        add("XEHelper["+value("XEHelperNum")+"]", "Mother ")

} if (selected("EXDB032", "3"))
        { add("XEHelperNum", value("XEHelperNum")+1)
        add("XEHelper["+value("XEHelperNum")+"]", "Father-in-law /father-in-law")

} if (selected("EXDB032", "4"))
        { add("XEHelperNum", value("XEHelperNum")+1)
        add("XEHelper["+value("XEHelperNum")+"]", "Mother-in-law /Mother-in-law")

} for (var i1 = 1; i1 < 26; i1++) {
        if (selected("EXDB033", i1) && selected("EXDB034[i1]", "1"))
                { add("XEHelperNum", value("XEHelperNum")+1)
                add("XEHelper["+value( "XEHelperNum")+"]", value("XEChildAliveName[i1]")+"I")

        } if (selected("EXDB033", i1) && selected("EXDB034[i1]", "2"))
                { add("XEHelperNum", value("XEHelperNum")+1)
                add("XEHelper["+value ("XEHelperNum")+"]", value("XEChildAliveName[i1]")+"spouse")

        } if (selected("EXDB033", i1) && selected("EXDB034[i1]", "3"))
                { add("XEHelperNum", value("XEHelperNum")+1)
                add("XEHelper["+value ("XEHelperNum")+"]", value("XEChildAliveName[i1]")+"child")
        }

} for (var i1 = 26; i1 <= 35; i1++) { if
        (selected("EXDB033", i1) && selected("EXDB034[i1]", "1"))
                { add("XEHelperNum", value( "XEHelperNum")+1)
                add("XEHelper["+value("XEHelperNum")+"]", value("EXDB033_1[i1]")+"myself")

        } if (selected("EXDB033", i1) && selected("EXDB034[i1]", "2"))
                { add("XEHelperNum", value("XEHelperNum")+1)
                add("XEHelper["+value ("XEHelperNum")+"]", value("EXDB033_1[i1]")+"spouse")

        } if (selected("EXDB033", i1) && selected("EXDB034[i1]", "3"))
                { add("XEHelperNum", value("XEHelperNum")+1)
                add("XEHelper["+value ("XEHelperNum")+"]", value("EXDB033_1[i1]")+"child")
        }

} for (var i1 = 1; i1 < 31; i1++) {
        if (selected("EXDB035", i1) && selected("EXDB036[i1]", "1"))
                { add("XEHelperNum", value("XEHelperNum")+1)
                add("XEHelper["+value( "XEHelperNum")+"]", value("XESibName[i1]")+"I")

        } if (selected("EXDB035", i1) && selected("EXDB036[i1]", "2"))
                { add("XEHelperNum", value("XEHelperNum")+1)
                add("XEHelper["+value ("XEHelperNum")+"]", value("XESibName[i1]")+"spouse")

        } if (selected("EXDB035", i1) && selected("EXDB036[i1]", "3"))
                { add("XEHelperNum", value("XEHelperNum")+1)
                add("XEHelper["+value ("XEHelperNum")+"]", value("XESibName[i1]")+"child")
        }

} for (var i1 = 31; i1 <= 40; i1++) { if
        (selected("EXDB035", i1) && selected("EXDB036[i1]", "1")) { add("XEHelperNum",
                value( "XEHelperNum")+1)
                add("XEHelper["+value("XEHelperNum")+"]", value("EXDB035_1[i1]")+"myself")
```

Machine Translated by Google


```
            } if (selected("EXDB035", i1) && selected("EXDB036[i1]", "2"))
                  { add("XEHelperNum", value("XEHelperNum")+1)
                  add("XEHelper["+value ("XEHelperNum")+"]", value("EXDB035_1[i1]")+"spouse")

            } if (selected("EXDB035", i1) && selected("EXDB036[i1]", "3"))
                  { add("XEHelperNum", value("XEHelperNum")+1)
                  add("XEHelper["+value ("XEHelperNum")+"]", value("EXDB035_1[i1]")+"child")
            }
      }
```

XEHelper Helper's identity/name

see above

XEHelpList selected main helpers

```
for (var i1 = 1; i1 < value("XEHelperNum")+1; i1++) { if
      ( greater("XEHelperNum", "7") && !equal("XEHelperNum", "7") && selected("EXDB039 ", i1) ) { add("XEHelpList[i1]",
            value("XEHelper[i1]"))
      }

} for (var i1 = 1; i1 < value("XEHelperNum")+1; i1++) { if ( !
      greater("XEHelperNum", "7") )
            { add("XEHelpList[i1]", value("XEHelper [i1]"))
      }
}
```

XESelectNum XSelectNum determines whether the number of selected helpers is more than 7

```
add("XESelectNum", "0")
for (var i1 = 1; i1 < 99; i1++) { if
      (selected("EXDB039", i1))
            { add("XESelectNum", value("XESelectNum")+1 )
      }
}
```

XEChildAndS If the child's name is not empty, it will display "XXX's spouse"

```
if (empty("ZChildName[i]"))
      { add("XEChildAndS[i]", "") }
else
      { add("XEChildAndS[i]", value("ZChildName[i]")+"'s spouse ")
}
```

XEChildGenderDis Displays the loaded gender in the question stem

```
if (equal("ZChildGender[i]", "1")) {
      add("XEChildGenderDis[i]", "Male") }
else if (equal("ZChildGender[i]", "2"))
      { add("XEChildGenderDis[i]", "Female") }
else
      { add("XEChildGenderDis[i]", "Missing")
}
```

XEChildAlive Whether the child is alive when accessed

```
if (equal("EXC001[i]", "1"))
      { add("XEChildAlive[i]", "1") }
else if (equal("EXC001[i]", "2")) { add
        ("XEChildAlive[i]", "0")
}
```

XEChildAliveName Name of the child who is alive at the time of access

```
if (equal("XEChildAlive[i]", "1"))
      { add("XEChildAliveName[i]", value("ZChildName[i]"))
}
```

XEChildBirthChildren 's birth year

Machine Translated by Google


```
if (!empty("ZChildBirth[i]"))
    { add("XEChildBirth[i]", value("ZChildBirth[i]")) } else

{ add("XEChildBirth[i]", value("EXC003[ i]"))
}
```

XEChildGenderChild gender

```
if (!empty("ZChildGender[i]"))
    { add("XEChildGender[i]", value("ZChildGender[i]")) } else

{ add("XEChildGender[i]", value("EXC004[ i]"))
}
```

XEChildEduThe highest level of education for children

```
if (!empty("ZChildEdu[i]"))
    { add("XEChildEdu[i]", value("ZChildEdu[i]")) } else

{ add("XEChildEdu[i]", value("EXC005[ i]"))
}
```

Medical insurance that **XEXMIns** participated in when he died

```
add("XEXMIns[1]", "Basic medical insurance for urban employees")
add("XEXMIns[2]", "Basic medical insurance for urban and rural residents (merging urban residents and new rural cooperative
medical insurance)") add("XEXMIns[3]", "Basic medical insurance
for urban residents") add("XEXMIns[4]", "New rural cooperative medical insurance
(cooperative medical insurance)")
add("XEXMIns[5]", "Public medical care")
add("XEXMIns[6]", "Medical assistance") add("XEXMIns[7]",
"Commercial medical insurance: purchased by the unit")
add("XEXMIns[8]", "Commercial medical insurance: purchased by
individuals") add("XEXMIns[9]", "Major disease
medical insurance for unemployed urban
residents") add("XEXMIns[10]", "Long-term care
    insurance") add("XEXMIns[12]", "No insurance") if (selected("EXEA001", "11")){ add("XEXMIns[11]", value("EXEA001_1"))
}
```

XEXHaveMins whether they have medical insurance when they die

```
if (selected("EXEA001", "1") || selected("EXEA001", "2" ) || selected( "EXEA001", " 3") ||
ÿ   selected("EXEA001", "4") || selected("EXEA001", "5") || selected("EXEA001", "6" ) ||
ÿ   selected("EXEA001", " 7") || selected("EXEA001", "8") || selected( "EXEA001", "9") ||
ÿ   selected("EXEA001", "10")|| selected("EXEA001", "11"))
    { add("XEXHaveMIns", "1")

} if (selected("EXEA001", "12"))
    { add("XEXHaveMIns", "0")
}
```

Does **XEXMInsPrivate** only have commercial medical insurance when he dies?

```
if (!empty("EXEA001"))
{ add("XEXMInsPrivate", " 0") }

if ((!selected("EXEA001", "1")) && (!selected("EXEA001", "2") ) && (!selected("EXEA001", "3")) && (!
ÿ   selected("EXEA001", " 4")) && (!selected("EXEA001", "5")) && (!selected("EXEA001 ", "6")) && (!
ÿ   selected("EXEA001", "9")) && (!selected("EXEA001", "10")) && (!selected("EXEA001", "11")) &&
ÿ   (selected("EXEA001", "7") || selected("EXEA001", "8")))
    { add("XEXMInsPrivate", "1")
}
```

Medical insurance previously participated in by **XEXPMIns**

```
add("XEXPMIns[1]", "Basic medical insurance for urban employees (medical
insurance)") add("XEXPMIns[2]", "Basic medical insurance for urban and rural residents (merging urban residents and new rural cooperative
medical insurance)") add("XEXPMIns[3]", "Basic medical insurance for
urban residents") add("XEXPMIns[4]", "New rural cooperative medical insurance (cooperative
medical insurance)") add("XEXPMIns[5]", "Public
medical care") add("XEXPMIns[6]", "Medical
assistance") add("XEXPMIns[7]", "Commercial medical insurance: purchased
by the unit") add("XEXPMIns[8]", "Commercial medical insurance: purchased by the individual")
```

Machine Translated by Google


```
add("XEXPMIns[9]", "Major disease medical insurance for
unemployed urban residents") add("XEXPMIns[10]",
"Long-term care insurance")
add("XEXPMIns[12]", "No insurance") if
    (selected("EXEB001", "11")){ add("XEXPMIns[11]", value("EXEB001_1"))
}
```

Has **XEXQuarantined** ever been quarantined?

```
if (selected("EXV004", "1") || selected("EXV004", "2" ) || selected("EXV004", "3") || selected("EXV004", ÿ "4")| |
 selected("EXV004", "5"))
    { add("XEXQuarantined", "1") }
else
    { add("XEXQuarantined", "0")
}
```

XEXVAHospitalizationExcluded wording: Isolation excludes hospitalization time

```
if (greater("EXV003", "0")) {
   add("XEXVAHospitalizationExcluded", "Excluding those days of hospitalization due to COVID-19,") }
```

XVCNotInQuarantine wording: Exclude quarantine during the epidemic

```
if (equal("XEXQuarantined", "1"))
    { add("XEXVCNotInQuarantine", ", does not include mandatory quarantine")
}
```

XKChildName generates a list of children with numbers 2-26

```
for (var k = 1; k <= 25; k++)
        { add("XKChildName["+(k+1)+"]", value("ZChildName[k]"))
}
```

XKChildAndS generates a list of children "spouse of XXX" with sequence numbers 27-51

```
for (var j = 1; j <= 25; j++)
        { add("XKChildAndS["+(j+26)+"]", value("XEChildAndS[j]"))
}
```

XEXGChildList1 generates a list of children's names from 1 to 35 based on the answer from EXG003

```
for (var i1 = 1; i1 <=25 ; i1++)
    { add("XEXGChildList1[i1]", value("ZChildName[i1]"))

} for (var i1 = 26; i1 <=35 ; i1++) {
    add("XEXGChildList1[i1]", value("EXG003_1[i1]"))
}
```

XEXGChildList4 generates a list of children's names from 1 to 35 based on the answer from EXG007

```
for (var i1 = 1; i1 <=25 ; i1++)
    { add("XEXGChildList4[i1]", value("ZChildName[i1]"))

} for (var i1 = 26; i1 <=35 ; i1++) {
    add("XEXGChildList4[i1]", value("EXG007_1[i1]"))
}
```

XEXGChildList2 generates a list of children's names from 1 to 35 based on the answer from EXG022

```
for (var i1 = 1; i1 <=25 ; i1++)
    { add("XEXGChildList2[i1]", value("ZChildName[i1]"))

} for (var i1 = 26; i1 <=35 ; i1++) {
    add("XEXGChildList2[i1]", value("EXG022_1[i1]"))
}
```

XEXGChildList5 generates a list of children's names from 1 to 35 based on the answer from EXG026

```
for (var i1 = 1; i1 <=25 ; i1++)
    { add("XEXGChildList5[i1]", value("ZChildName[i1]"))
}
```

Machine Translated by Google


```
for (var i1 = 26; i1 <=35 ; i1++)
      { add("XEXGChildList5[i1]", value("EXG026_1[i1]"))
}
```

XEXGChildList3 generates a list of children's names from 1 to 35 based on the answer from EXG032

```
for (var i1 = 1; i1 <=25 ; i1++)
      { add("XEXGChildList3[i1]", value("ZChildName[i1]"))

} for (var i1 = 26; i1 <=35 ; i1++)
      { add("XEXGChildList3[i1]", value("EXG032_1[i1]"))
}
```

XEXGChildList6 generates a list of children's names from 1 to 35 based on the answer from EXG036

```
for (var i1 = 1; i1 <=25 ; i1++)
      { add("XEXGChildList6[i1]", value("ZChildName[i1]"))

} for (var i1 = 26; i1 <=35 ; i1++)
      { add("XEXGChildList6[i1]", value("EXG036_1[i1]"))
}
```

XEXGTotalValue determines the total value of the estate excluding real estate. If an estate is empty or -1, it is replaced by 0.

```
add("XCK_EXF005", value("EXF005"))
if (empty("XCK_EXF005") || equal("XCK_EXF005", "-1"))
   { add("XCK_EXF005", "0")
}

add("XCK_EXF007", value("EXF007"))
if (empty("XCK_EXF007") || equal("XCK_EXF007", "-1"))
   { add("XCK_EXF007", "0")
}

add("XCK_EXF009", value("EXF009"))
if (empty("XCK_EXF009") || equal("XCK_EXF009", "-1"))
   { add("XCK_EXF009", "0")
}

add("XCK_EXF011", value("EXF011"))
if (empty("XCK_EXF011") || equal("XCK_EXF011", "-1"))
   { add("XCK_EXF011", "0")
}

add("XCK_EXF013", value("EXF013"))
if (empty("XCK_EXF013") || equal("XCK_EXF013", "-1"))
   { add("XCK_EXF013", "0")
}

add("XCK_EXEB006", value("EXEB006"))
if (empty("XCK_EXEB006") || equal("XCK_EXEB006", "-1"))
   { add("XCK_EXEB006", "0")
}

add("XCK_EXG010", value("EXG010"))
if (empty("XCK_EXG010") || equal("XCK_EXG010", "-1"))
   { add("XCK_EXG010", "0")
}

add("XCK_EXG012", value("EXG012"))
if (empty("XCK_EXG012") || equal("XCK_EG012", "-1"))
   { add("XCK_EXG012", "0")
}

add("XCK_EXG014", value("EXG014"))
if (empty("XCK_EXG014") || equal("XCK_EXG014", "-1"))
   { add("XCK_EXG014", "0")
}

add("XCK_EXG016", value("EXG016"))
if (empty("XCK_EXG016") || equal("XCK_EXG016", "-1"))
   { add("XCK_EXG016", "0")
}
```

Machine Translated by Google


```
add("XCK_EXG017", value("EXG017"))
if (empty("XCK_EXG017") || equal("XCK_EXG017", "-1"))
   { add("XCK_EXG017",
"0") }

add("XEXGTotalValue", value("XCK_EXF005")+value("XCK_EXF007")+value("XCK_EXF009")+value("XCK_EXF011")+va ÿ
ÿ     lue("XCK_EXF013")+value("XCK_EXEB006")+ value("XCK_EXG010")+value("XCK_EXG012")+value("XCK_EXG014")
ÿ     +v ÿ alue("XCK_EXG016")+value("XCK_EXG017"))
```

Machine Translated by Google

This page intentionally left blank

Machine Translated by Google

Appendix Function Description

Machine Translated by Google

A. The function in the question

The **Hard** Check function hc(a,b,c) is used to avoid logically impossible records of numeric variables. If the record of the corresponding question does not meet the conditions in hc, the interviewer is required to return to modify the answer. Parameter Description: • a: value range • b: indicates that only integers can be filled in, indicates that decimals can be filled in • c: the symbol when the respondent refuses to answer or does not know the corresponding question, ÿ indicates that such records are not allowed

The **Soft Check** function sc(a,b,c) is used to alert abnormal values of numerical variables. If the record of the corresponding question does not meet the conditions in sc, the interviewer is required to confirm again. The parameters of sc(a,b,c) are exactly the same as those of the hard check function.

The Unfolding **Brackets** function ub(a,b) is used to obtain the range information of a specific amount of money (such as income) when the respondent refuses to answer or is not clear about the specific amount of money. Parameter description: • a: array, indicating the triggering condition of the unfolding bracket, that is, as long as the record of the corresponding question is in the array, the unfolding bracket is triggered. Expand • b: array, indicating the breakpoints of the hierarchical expansion. The mark for refusing to answer or not knowing in the hierarchical expansion questions is "-1".

The **conflict** checking function conflict(a,b,…,c) is used to check whether there are logical errors in the input answers. If the last option c is selected at the same time as any of the previous options, an error will be prompted and the interviewer must reconfirm and update the answer.

Transfer Picture The picture display function transferPic() is used to display pictures.

B. Functions in auxiliary variables

The auxiliary variables in the questionnaire are calculated from the original variables based on preset rules. The construction of these auxiliary variables involves some basic logical control structures, such as if-else-if and for loops, and also includes some custom functions. The current questionnaire involves the following custom functions:

• add(x,y): Set the value of variable x to y • value(x): Return the value of variable x • selected(x,y): Determine whether y is in array x, generally used to determine whether a certain option is selected in the corresponding multiple-choice question If selected, returns true, otherwise returns false

• count(x): Returns the size of array x (i.e., the number of elements), generally used to determine the number of selected options in a multiple-choice question • range(x,min,max,decimal,special): Determines whether variable x meets the set range, min indicates the lower limit of the range, max indicates the upper limit of the range, decimal indicates whether decimals are allowed (true indicates that decimals are allowed, false indicates that decimals are not allowed), special indicates the specially allowed values outside the range (missing indicates no) • greater(x,y,equal): Determines whether variable x is greater than or greater than or equal to y, equal is used to control whether the comparison is greater than or equal to Greater than or equal to (true means the comparison is greater than or equal to, missing means the comparison is greater than)

• equal(x,y): Determines whether variable x is equal to y, and returns true if it is equal. • empty(x): Determines whether variable x is empty or does not exist, and returns true if it is empty or does not exist.
